# Final Results of a Randomized, Placebo-Controlled, Two-Arm, Parallel Clinical Trial of Proxalutamide for Hospitalized COVID-19 Patients: A Multiregional, Joint Analysis of the Proxa-Rescue AndroCoV Trial

**DOI:** 10.7759/cureus.20691

**Published:** 2021-12-25

**Authors:** Flavio A Cadegiani, Ricardo A Zimerman, Daniel N Fonseca, Michael N Correia, Marcio P Muller, Diego Leonardo Bet, Marcio Rafael Slaviero, Ivan Zardo, Paulo Roberto Benites, Renan N Barros, Raysa W Paulain, Dirce C Onety, Karla Cristina P Israel, Carlos Gustavo Wambier, Andy Goren

**Affiliations:** 1 Internal Medicine, Corpometria Institute, Brasilia, BRA; 2 Infectious Disease, Hospital da Brigada Militar de Porto Alegre, Porto Alegre, BRA; 3 Internal Medicine, Samel & Oscar Nicolau Hospitals, Manaus, BRA; 4 Internal Medicine, Hospital Regional José Mendes, Itacoatiara, BRA; 5 Surgery, Hospital Arcanjo São Miguel, Gramado, BRA; 6 Internal Medicine, Hospital Unimed Chapecó, Chapecó, BRA; 7 Physical Medicine and Rehabilitation, Hospital Arcanjo São Miguel, Gramado, BRA; 8 Cardiology, Hospital Unimed Chapecó, Chapecó, BRA; 9 Pulmonary and Critical Care, Hospital Unimed Chapecó, Chapecó, BRA; 10 Internal Medicine, Hospital Municipal Jofre Cohen, Parintins, BRA; 11 Critical Care, Samel & Oscar Nicolau Hospitals, Manaus, BRA; 12 Nephrology, Samel & Oscar Nicolau Hospitals, Manaus, BRA; 13 Dermatology, The Warren Alpert Medical School of Brown University, Providence, USA; 14 Dermatology, Applied Biology Inc., Irvine, USA

**Keywords:** proxalutamide, tmprss2, pandemic, transmembrane protease serine 2, clinical trial, sepsis, antiandrogens, androgen receptor, sars-cov-2, covid-19

## Abstract

Background

The role of androgens on COVID-19 is well established. Proxalutamide is a second-generation, non-steroidal antiandrogen (NSAA) with the highest antiandrogen potency among NSAAs and concurrent regulation of angiotensin-converting enzyme 2 (ACE2) expression and inflammatory response. Proxalutamide has been demonstrated to be effective to prevent hospitalizations in early COVID-19 in randomized clinical trials (RCTs). Conversely, in hospitalized COVID-19 patients, preliminary results from two different arms of an RCT (The Proxa-Rescue AndroCoV Trial) also demonstrated a reduction in all-cause mortality. This study aims to report the final, joint results of the two arms (North arm and South arm) of the Proxa-Rescue AndroCoV trial of the two arms (North and South arms) combined, and to evaluate whether COVID-19 response to proxalutamide was consistent across different regions (Northern Brazil and Southern Brazil).

Materials and methods

Upon randomization, hospitalized COVID-19 patients received either proxalutamide 300mg/day or placebo for 14 days, in addition to usual care, in a proxalutamide:placebo ratio of 1:1 in the North arm and 4:1 in the South arm (ratio was modified due to preliminary report of high drug efficacy). Datasets of the South and North arms were combined, and statistical analysis was performed for the overall study population. Proxalutamide was compared to placebo group for 14-day and 28-day recovery (discharge alive from the hospital) and mortality rates, and overall and post-randomization hospitalization stay. Results of proxalutamide and placebo groups were also compared between the North and South arms. Analysis was also performed stratified by sex and baseline WHO COVID Ordinary Score.

Results

A total of 778 subjects were included (645 from the North, 317 from the proxalutamide group and 328 from the placebo group; 133 from the South arm, 106 from the proxalutamide group and 27 from the placebo group). Recovery rate was 121% higher in proxalutamide than placebo group at day 14 [81.1% vs 36.6%; Recovery ratio (RecR) 2.21; 95% confidence interval (95% CI), 1.92-2.56; p<0.0001], and 81% higher at day 28 (98.1% vs 47.6%; RecR, 1.81; 95% CI, 1.61-2.03; p<0.0001). All-cause mortality rate was 80% lower in proxalutamide than placebo group at Day 14 [8.0% vs 39.2%; Risk ratio (RR), 0.20; 95% CI, 0.14-0.29; p<0.0001], and 78% lower at Day 28 (10.6% vs 48.2%; RR, 0.22; 95% CI 0.16-0.30). Post-randomization time-to-discharge was shorter in proxalutamide [median, 5 days; interquartile range (IQR), 3-8] than placebo group (median, 9 days; IQR, 6-14) (p<0.0001). Results were statistically similar between North and South arms for all measured outcomes. Males and females presented similar results in all outcomes. Patients that did not require oxygen use (scores 3 and 4) did not present statistically significant improvement in recovery and mortality rates, whereas scores 5 and 6 presented significant improvements in all outcomes (p<0.0001 for all).

Conclusion

Proxalutamide increased recovery rate, reduced mortality rate and shortened hospital stay in hospitalized COVID-19 patients. Results were similar between the two different arms, providing further consistency for the efficacy of proxalutamide when used in late-stage COVID-19.

## Introduction

Early in 2020, when the pandemics of the severe acute respiratory syndrome coronavirus 2 (SARS-CoV-2) and its resulting disease, coronavirus disease 2019 (COVID-19), was announced, early reports already indicated that males were an independent risk factor for severe COVID-19 and death, since the overrepresentation of males was not fully justified by the presence of comorbidities, body mass index (BMI), habits or age [1,2].

The observation that males with androgenetic alopecia (AGA) were prevailing in COVID-19 intensive care units (ICUs) allowed different teams of researchers to hypothesize and was further confirmed that male AGA would also be an independent risk factor for severe COVID-19 [3-6]. In addition, anabolic androgenic steroid (AAS) users may be at higher risk for COVID-19 [7]. Hypersensitivity of the androgen receptor (AR) revealed through the AR gene was demonstrated to be a determinant of COVID-19 prognosis [8,9]. Women with hyperandrogenic states, including polycystic ovary syndrome (PCOS), experimented more severe states of COVID-19 [10,11].

Accordingly, chronic users of anti-androgens progressed with milder symptoms and less severe COVID-19 states [12,13], in particular subjects under androgen deprivation therapy (ADT) for castration-resistant prostate cancer [14-17].

Collectively, in both males and females, not only hyperexpression and hyperactivity of androgens are possibly a major predictor of COVID-19, but AR suppression can reduce the severity of COVID-19. Altogether, the observations from these studies reinforce the androgen theory for COVID-19.

The biological plausibility for the androgen theory on COVID-19 is corroborated by the fact that SARS-CoV-2 cell entry largely depends on an endogenous transmembrane protease, serine 2 (TMPRSS2), that primes the virus, a critical step for its entrance through the coupling to the angiotensin-converting enzyme 2 (ACE2) receptors [18], and the inhibition of TMPRSS2 would reduce SARS-CoV-2 infectivity. The only known endogenous regulators of the TMPRSS2 expression are the androgens [19]. The virus dependence on TMPRSS2 varies across different variants [20,21].

From all the consisting observations and strong, concordant mechanistic explanation, we hypothesized that anti-androgens could be a potential target to protect against COVID-19 [22,23].

Dutasteride, a broad 5alpha-reductase inhibitor, demonstrated to reduce viral load and accelerate the recovery process in early COVID-19 [24]. Proxalutamide, a second-generation nonsteroidal androgen receptor antagonist initially developed to be an additional option for ADT in males with castration-resistant prostate cancer, was shown to be more potent than other antiandrogen compounds such as enzalutamide or bicalutamide in inhibiting androgen activity, and presented concurrent activity on ACE-2, as well as anti-inflammatory properties [25].

Proxalutamide was shown to reduce hospitalization rate in both males [26] and females [27], and induced significant reduction in inflammatory and thrombotic markers, which was particularly observed in subjects with initial high ultrasensitive C-reactive (usCRP) and D-dimer levels [28,29]. The fact that proxalutamide may not be restricted to antiviral activity in COVID-19, but also act as a strong anti-inflammatory, anti-thrombotic, and possibly immunomodulatory agent, allowed the hypothesis that proxalutamide could also be effective to COVID-19 in later, more severe stages, during hospitalization.

As expected, proxalutamide demonstrated a reduction of more than 70% in mortality rate and increase of more than 100% in recovery rate at Day 14 in two distinct geographical regions, in a randomized, double-blind, placebo-controlled, multiregional clinical trial with two different arms (Northern Brazil and Southern Brazil) [30-33]. Due to the unexpected efficacy of proxalutamide, further confirmation of the findings was critical. The use of the exact same protocol with the ethical adaptations to a different region could help indicate whether these findings were consistent and increase the statistical power of the analysis while fully respecting ethical issues.

The present study is the joint analysis of the randomized clinical trial (RCT) that tested proxalutamide on patients hospitalized due to COVID-19 conducted in two different regions, as two different arms, aiming to provide the final results for the Proxa-Rescue AndroCoV Trial and to evaluate whether findings were consistent between arms.

## Materials and methods

Design

This is a joint analysis of the arms of a double-blinded, placebo-controlled, two-arm (arm as group population - two groups) parallel RCT conducted in two different regions of Brazil - Northern Brazil (North arm) and Southern Brazil (South arm) (arm as a completely distinct study within the same RCT protocol) - at 11 hospital settings in nine cities. The North arm included the following hospitals and respective cities: Samel Hospital, Oscar Nicolau Hospital and Hospital ProntoCord, in Manaus, Hospital Regional José Mendes, in Itacoatiara, Hospital Regional Jofre Cohen, in Parintins, Hospital de Campanha de Manacapuru, in Manacapuru, Hospital Regional Dr. Hamilton Maia Cidae, in Manicore, and Hospital Raimiunda Francisca Dinelli da Silva, in Maues. All hospitals and cities in the North arm are located in the state of Amazonas (AM), in the Northern region of Brazil. The South arm included the following hospitals and respective cities and states: Hospital Arcanjo São Miguel, in Gramado, state of Rio Grande do Sul (RS), Hospital da Brigada Militar de Porto Alegre (HBMPA), in Porto Alegre, state of Rio Grande do Sul (RS), and Hospital Unimed Chapecó, in Chapeço, state of Santa Catarina (SC). Both states are located in the Southern region of Brazil.

The protocol of the present study was approved by the National Ethics Committee (Comitê de Ética em Pesquisa da Comissão Nacional de Ética em Pesquisa - Ministério da Saúde CEP/CONEP/MS) (approval number 4.513.425; CAAE 41909121.0.0000.5553) in January 27th, 2021, and is registered in clinicaltrials.gov as two different numbers: the North arm (NCT04728802) and the South arm (NCT05126628). Dataset is publicly available in the appendix.

Study population

Patients with known COVID-19 (positive test SARS-CoV-2 real-time reverse transcription polymerase chain reaction (rtPCR) testing; Cobas SARS-CoV-2 rtPCR kit test protocol, Roche, USA) that had progression of the disease and required hospitalization in the past seven days of recruitment and were above 18 years old were considered for the study.

Invasive mechanical ventilation, liver damage, as seen through alanine transferase (ALT) levels above 250 U/L, kidney injury, as seen through creatinine above 2.5 mg/ml or a calculated eGFR below 30 ml/min, known class III or IV congestive heart failure, use of immunosuppressors or antiandrogens, or being pregnant (women), breastfeeding (women), or planning to conceive within 90 days after randomization (both sexes) were excluded. However, as per the majority of the hospital protocols, conceiving within three to six months after moderate-to-severe COVID-19 was discouraged.

Once eligibility criteria were fulfilled, candidates for the present study received detailed explanation about the characteristics of the study, including the definition, concepts and practical examples of the characteristics of a placebo-controlled study, a double-blind study, and randomized clinical trial study, and received explanations about the drug (proxalutamide), including what this drug was primarily used for, the lack of approval in any country at the time of the study, potential risks, uncertainties and rights that participants had at any time of the study, followed by expected effects and potential yet unproven benefits. Agreement from potential candidates was never accepted at the first approach to avoid participation based on the vulnerability of the critical state of the majority of the patients at the time of the study. Candidates kept the consent form for a while (not a specific period of time; from 20 to 30 minutes until hours) to read it thoroughly, and, in case of illiteracy, a non-illiterate family member or a staff from the research team read the full consent form for the candidate. Subjects who agreed to participate signed the written consent form as approved by the National Ethics Committee.

Procedures

Patients were randomized to receive either proxalutamide 300 mg/day plus usual care or a placebo plus usual care for 14 days. The proxalutamide:placebo randomization ratio was 1:1 in the North arm and 4:1 ratio in the South arm. Patients discharged alive from the hospital before 14 days of treatment were instructed to continue treatment and strongly recommended not to interrupt before 14 days without communicating the hospital staff, due to the uncertain effects of the interruption of treatment before 14 days. Therapy compliance in both hospital and at home was actively monitored.

Randomization presented differences between the North and South arms due to logistical difficulties in the Northern region of Brazil, and are detailed elsewhere [30,31].

Usual care varied according to each hospital’s protocol but always included a glucocorticoid (methylprednisolone or dexamethasone), since the vast majority required oxygen use upon hospitalization. Other medications could include omeprazole, anticoagulant (enoxaparin in almost all cases), colchicine, and antibiotic therapy if necessary. During the 28-day study period, additional medications that each patient required were quantified individually.

Baseline characteristics, including age, biological sex and self-identified gender (in case of a non-cis person), presence of type 2 diabetes mellitus (T2DM), hypertension, cardiovascular diseases, chronic obstructive pulmonary disease (COPD), chronic renal disease (CKD) and other coexisting conditions, concomitant medications and baseline clinical severity level were recorded for each patient included in the study.

The COVID-19 8-point ordinal scale [34] was the main driver to classify and detect outcomes, since it classifies according to the clinical severity level, reveals and distinguishes patients that are discharged alive from the hospital, on oxygen use, higher oxygen need, on mechanical ventilation, and that died, according to the score. The scale was used for screening (Day 0), from Day 1 to Day 14, Day 21 and Day 28 (or until discharge or death). The clinical score is defined as: 1. Not hospitalized, no limitations on activities; 2. Also not hospitalized, but on the limitation on activities; 3. Hospitalized, but not or no longer requiring supplemental oxygen or continuous medical care; 4. Hospitalized, not requiring supplemental oxygen, but requiring ongoing medical care, whether related to COVID-19 or not; 5. Hospitalized and requiring oxygen use; 6. Hospitalized and requiring higher oxygen inflow, including high-flow oxygen devices or non-invasive ventilation; 7. Hospitalized, on invasive mechanical ventilation; and 8. Death.

Participants discharged from the hospital alive were actively monitored by local investigators, with post-discharge medical appointments in the same sites where they were hospitalized. Upon discharge from the hospital, patients were instructed to return in case of relapse, worsening, or new symptoms. Hospital readmissions were actively surveilled in all sites and informed to the supervising research team.

Local investigators that recruited and/or followed participants directly were kept blinded until all patients completed the study. The principal investigator was blinded for the North arm until March 10, 2021, and unblinded for the South arm. A daily analysis was performed by the principal investigator, and a weekly analysis by a data safety monitoring board (DSMB) and an interim analysis was performed weekly, unless a period of recruitment was shorter than 14 days (time to receive the first report from the DSMB).

Protocol modification

Due to the high efficacy reported in the first state where the study was conducted, the principal investigator decided to change the active:placebo ratio from 1:1 to 4:1, aiming to reduce the exposure to placebo. As per the protocol approved, we could not continue the study as a single-arm study, since this is not permitted in the country where it was conducted (Brazil). The randomization method was changed from blocks to individualized sets.

Efficacy assessment

The primary outcome to evaluate the efficacy of proxalutamide in patients hospitalized due to COVID-19 was the recovery rate 14 days after randomization, based on scores 1 and 2 at the 8-point COVD-19 ordinal scale, or the proportion of patients discharged from hospital alive before 14 days of treatment.

Secondary outcomes included recovery rate (scores 1 and 2) at Day 28, all-cause mortality rate (score 8) at Day 14 and at Day 28, hospitalization stay (days) and time-to-discharge alive after randomization (days).

The efficacy of the recovery and all-cause mortality rates was measured through the ratio between the rates of the proxalutamide group and the placebo group, and the efficacy of the other two outcomes was determined by the comparison between the groups.

Safety assessment

Treatment-emergent adverse effects (TEAEs) were actively searched. Grades 3 to 5 severe adverse effects (SAEs) measured included death, mechanical ventilation, shock requiring vasopressors, liver damage, kidney injury and disease progression. Grades 1 and 2 adverse effects (AEs) actively questioned were gastrointestinal symptoms (diarrhea, vomiting, nauseas, abdominal pain, and dyspepsia), palpitations and irritability. Other symptoms were spontaneously reported.

Presentation of the results

The primary results of the North arm and the South arm comparing the proxalutamide group with the placebo group of each arm were presented elsewhere [30,31]. In this joint analysis, different comparisons were performed.

Proxalutamide versus placebo

Active groups from the South and North arms and placebo groups from the South and North arms were combined to analyze the efficacy of proxalutamide compared to placebo in an overall, multicentric, multiregional perspective. Baseline characteristics, outcomes and adverse effects (AEs) were compared between active groups combined and placebo groups combined.

Baseline characteristics were compared for age, sex, comorbidities, time from hospitalization to randomization, and medications used during hospitalization. Outcomes were compared for 14-day and 28-day recovery rate, 14-day and 28-day mortality rate, hospitalization length stay and post-randomization time-to-discharge alive from hospital. Outcomes were also compared between subgroups according to sex and baseline clinical score, except for hospitalization overall and post-randomization length stay.

North versus South

Results in the North arm were compared to the results in the South arm. The analysis was performed by comparing proxalutamide group of the North arm with the proxalutamide group of the South arm, and the placebo group of the North arm was compared to the placebo group of the South arm. Comparisons were performed for the same baseline characteristics, outcomes and adverse effects compared between combined active and combined placebo groups. Outcomes were compared overall and according to sex.

Absolute differences between active and placebo groups from each arm were calculated, estimating the number of patients that would have been benefited from the intervention if all participants of that arm received proxalutamide. The number was provided as the number of patients expected to be benefited over the overall number of patients of the respective arm. Absolute differences were then compared between the North and the South arm to evaluate whether the level of efficacy was similar between arms or not, for recovery and mortality rates at Day 14 and Day 28, for overall and sex-specific.

**Figure 1:**
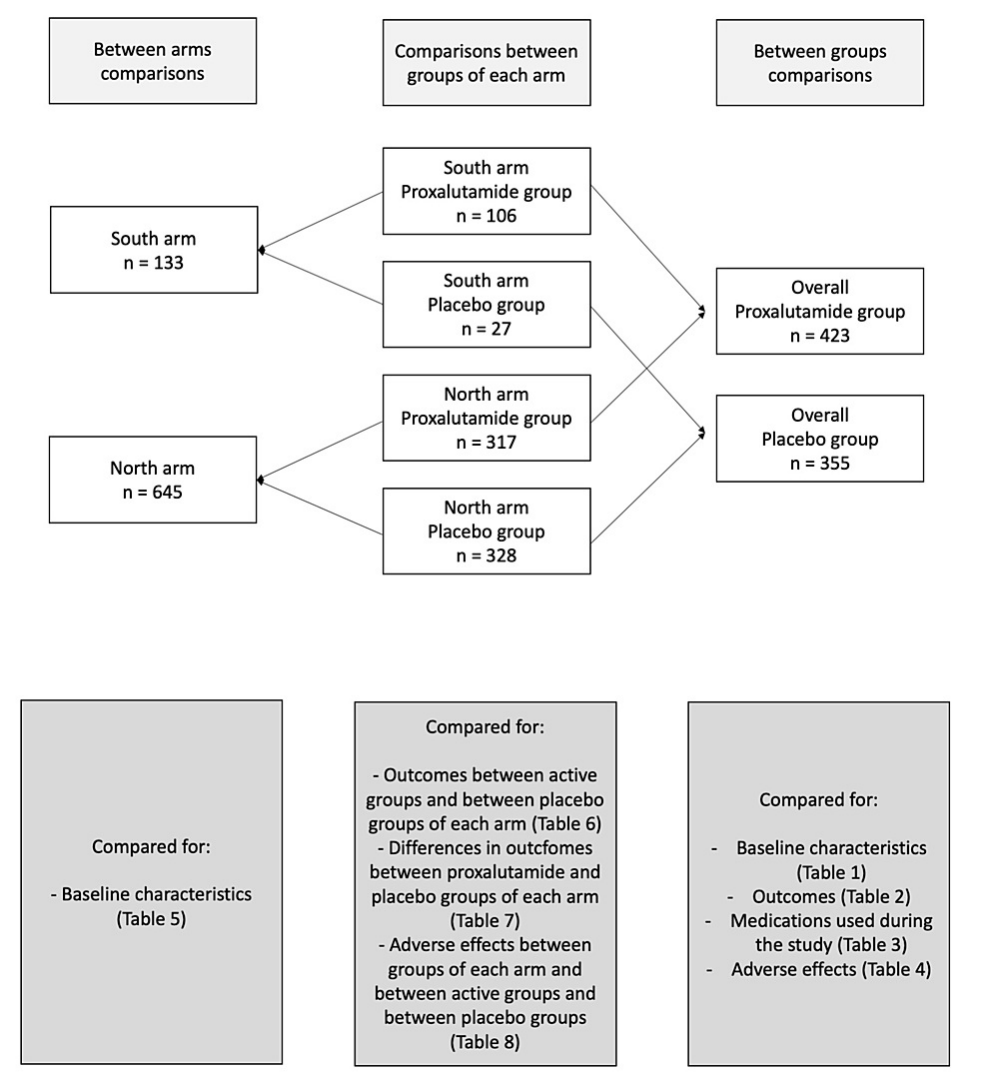
Illustrative description of the comparisons performed in the present analysis.

We did not compare the outcomes and adverse effects of overall participants between the North and the South arms due to the difference between the proxalutamide:placebo ratio, which would lead to confounding differences. Figure 1 illustrates the comparisons that were performed between different groups and arms throughout this analysis.

Statistical analysis

In the North arm, intention-to-treat (ITT) protocol was employed, whereas in the South arm a modified ITT (mITT) was employed. All subjects that remained at least 24 hours after the first dose of proxalutamide or placebo were considered for the mITT. The difference occurred because in the North arm sites were unable to communicate the discontinuation of the drug within less than 24 hours after randomization, which did not allow the employment of the mITT, whereas this communication was instantaneous in the South arm. Indeed, in the North arm, ITT and mITT would not lead to any differences.

To determine the efficacy of proxalutamide compared to usual care, combined proxalutamide groups (i.e., proxalutamide group from the South and North arms together) were compared to combined placebo groups (i.e., placebo group from the South and North arms together). Cox proportional hazard model was employed to calculate the hazard ratio (HR) and 95% confidence interval (95% CI), for 14-Day and 28-Day all-cause mortality rate, and the rate ratio (RR) of the 14-Day and 28-Day recovery rate. The Wilcoxon Rank Sum and Wilcoxon-Mann-Whitney U tests were employed to compare the differences of the ordinal scale scores at Day 14, length of hospitalization stay, and post-randomization time-to-discharge alive from the hospital, and the following baseline characteristics: median age and interval between hospitalization and randomization. Chi-Square Test was employed to compare the other baseline characteristics and TEAEs. XLSTAT 2021.5 (Addinsoft, Paris, France) and Easy Med Stat were used to run the statistical analysis.

To evaluate the level of similarity between the North and South arms, baseline characteristics of each arm were compared using the same statistical tools as used to compare proxalutamide and placebo groups. To assess the consistency between arms, the outcomes of the active and placebo groups from the North arm were compared to the respective groups from the South arm using Cox proportional hazard model and the TEAES were compared using Chi-Square Test.

To assess whether there were differences between the level of efficacy of proxalutamide arms between regions, the estimated number of additional subjects to be benefited from the treatment if all participants were treated with proxalutamide were calculated for all outcomes in the North and in the South arm, and then compared using Cox proportional hazard model.

Similar statistical tools were employed for the comparative analysis of sex- and baseline COVID ordinary score-specific subpopulations for the corresponding outcomes and TEAEs.

## Results

**Figure 2:**
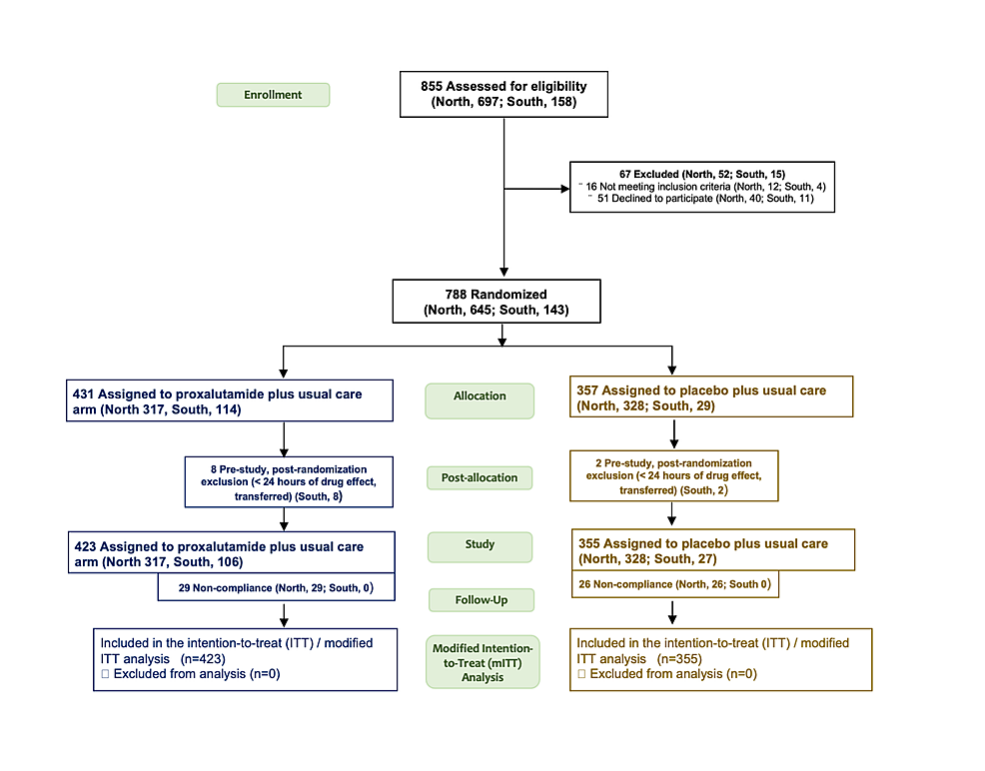
CONSORT Flowchart.

A total of 778 subjects were included in the study, including 645 from the North arm (317 from the proxalutamide group and 328 from the placebo group), and 133 from the South arm (106 from the proxalutamide group and 27 from the placebo group) of the Proxa-Rescue AndroCoV trial. Figure 2 depicts the CONSORT flowchart. No transmen, transwomen, non-binary, or intersex subjects were included.

Proxalutamide versus placebo

Table 1 describes the baseline characteristics of overall, proxalutamide, and placebo groups resulted from the joint analysis of the North and the South arms. Except for the use of medications, none of the characteristics was statistically significantly different between groups. However, participants from the proxalutamide group were marginally older (p=0.062) and had marginally more type 2 diabetes (p=0.055) than the placebo group. None of the groups presented patients with chronic kidney disease (CKD), since this was criteria for exclusion per the protocol.

**Table 1:** Baseline characteristics of the overall, proxalutamide and placebo groups of the North and South arms combined.

Characteristic	Overall N=778	Proxalutamide N=423	Placebo N=355	p-value
Age				
Median – years (IQR)	51 (41-62)	52 (42-62)	51 (39-62)	0.062
> 55 yr – no. (%)	296 (38.0%)	159 (37.6%)	137 (38.6%)	0.77
Sex – no. (%)				0.26
Female	314 (40.4%)	163 (38.5%)	151 (42.5%)	
Male	464 (59.6%)	260 (61.5%)	204 (57.5%)	
Comorbidities				
Body mass index over 30 kg/m^2^ – no. (%)	68 (8.7%)	39 (9.2%)	29 (8.2%)	0.70
Hypertension – no. (%)	215 (27.6%)	124 (29.3%)	91 (25.6%)	0.26
Type 2 diabetes mellitus – no. (%)	97 (12.5%)	55 (13.0%)	42 (11.8%)	0.055
Chronic obstructive pulmonary disorder – no. (%)	18 (2.3%)	9 (2.1%)	9 (2.5%)	0.81
Chronic kidney disease – no. (%)	0	0	0	n/a
Coexisting conditions – no. (%)				
0	507 (65.2%)	267 (63.1%)	240 (67.6%)	0.19
1	157 (20.2%)	92 (21.7%)	65 (18.3%)	0.26
2+	114 (14.6%)	64 (15.1%)	50 (14.1%)	0.68
Median time from hospitalization to randomization (IQR) – days	2 (1-4)	2 (1-4)	2 (1-4)	0.16
Score on the Coronavirus Disease 2019 ordinal scale – no. (%)				
3. Hospitalized, not requiring supplemental oxygen - no longer requires ongoing medical care	2 (0.3%)	0	2 (0.6%)	0.25
4. Hospitalized, not requiring supplemental oxygen, requiring ongoing medical care (COVID-19 related or otherwise)	24 (3.1%)	12 (2.8%)	12 (3.3%)	0.66
5. Hospitalized, requiring supplemental oxygen	252 (31.4%)	136 (32.2%)	116 (32.7%)	0.88
6. Hospitalized, receiving non-invasive ventilation or high flow oxygen devices	500 (64.3%)	275 (65.0%)	225 (63.4%)	0.64

**Figure 3:**
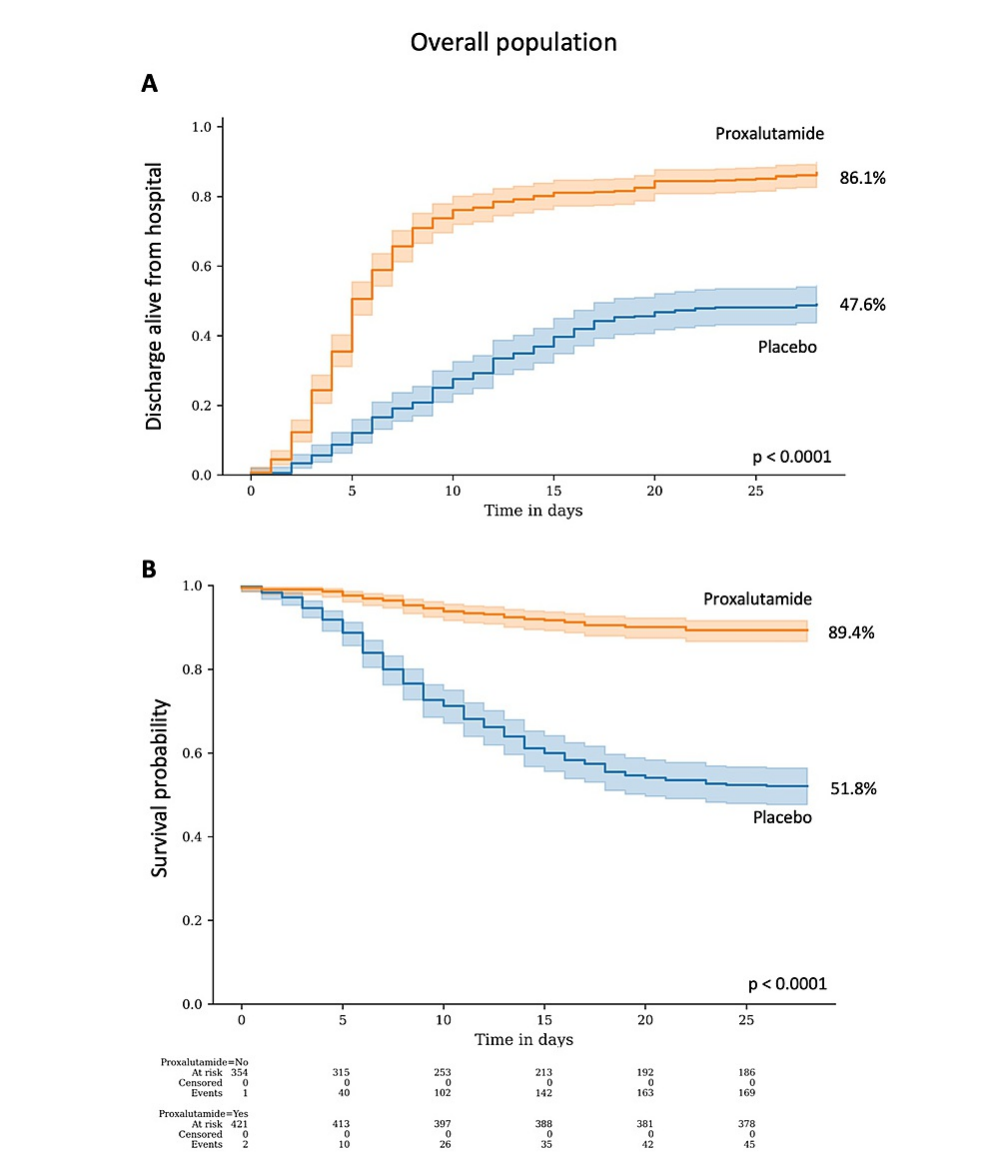
Proxalutamide group compared to placebo group. A. Proportion of patients discharged alive from the hospital; B. Kaplan-Meier survival probability.

Table 2 depicts the results of the outcomes in the combined overall, combined proxalutamide, and combined placebo groups. Figure 3 details the proportion of patients discharged alive from the hospital and the Kaplan-Meier survival estimate.

**Table 2:** Outcomes in overall study population, stratified to sex and baseline score. IQR = Interquartile range; 95% CI = 95% confidence interval; sc. = score; NNT = number needed to treat; n/a = Non-applicable

Characteristic	Overall N=778 (females = 314) (males = 464) (sc. 3/4 = 26) (sc. 5 = 253) (sc. 6 = 499)	Proxalutamide N=423 (females = 163) (males = 260) (sc. 3/4 = 12) (sc. 5 = 137) (sc. 6 = 274)	Placebo N=355 (females = 151) (males = 204) (sc. 3/4 = 14) (sc. 5 = 116) (sc. 6 = 225)	Risk ratio (95% CI) NNT | p-value
Day 14 Scores – median (IQR)	2 (1-7)	1 (1-2)	6 (2-8)	p < 0.0001
Recovery rate over 14 days, no. (%)	473 (60.8%)	343 (81.1%)	130 (36.6%)	2.21 (1.92 – 2.56) NNT = 2.2 | p < 0.0001
Females	185 (58.9%)	132 (81.0%)	53 (35.1%)	2.31 (1.83 – 2.91) p<0.0001 | NNT=2.2
Males	288 (62.1%)	211 (81.2%)	77 (37.7%)	2.15 (1.79 – 2.59) p<0.0001 | NNT=2.3
Baseline Scores 3 and 4	22 (84.6%)	10 (83.3%)	12 (85.7%)	0.97 (0.70 – 1.35) p=0.87 | NNT=n/a
Baseline Score 5	183 (72.3%)	123 (89.8%)	60 (51.7%)	1.73 (1.44 – 2.09) p<0.0001 | NNT=2.6
Baseline Score 6	268 (53.7%)	210 (76.6%)	58 (25.8%)	2.97 (2.36 – 3.75) p<0.0001 | NNT=2.0
Recovery rate over 28 days no. (%)	533 (68.5%)	364 (86.1%)	169 (47.6%)	1.81 (1.61 – 2.03) NNT = 2.6 | p < 0.0001
Females	215 (68.5%)	142 (87.1%)	73 (48.3%)	1.80 (1.51 – 2.15) p<0.0001 | NNT=2.6
Males	318 (68.5%)	222 (85.4%)	96 (47.1%)	1.81 (1.56 – 2.12) p<0.0001 | NNT=2.6
Baseline Scores 3 and 4	23 (88.5%)	11 (91.7%)	12 (85.7%)	1.07 (0.81 – 1.41) p=0.63 | NNT=16.8
Baseline Score 5	203 (80.2%)	128 (93.4%)	75 (64.7%)	1.45 (1.25 – 1.67) p<0.0001 | NNT=3.5
Baseline Score 6	307 (61.5%)	225 (82.1%)	82 (36.4%)	2.25 (1.88 – 2.70) p<0.0001 | NNT=2.2
All-cause mortality rate over 14 days, no. (%)	172 (22.1%)	34 (8.0%)	138 (38.9%)	0.21 (0.15 – 0.30) NNT = 3.2 | p < 0.0001
Females	66 (21.0%)	11 (6.7%)	55 (36.4%)	0.18 (0.10 – 0.34) p<0.0001 | NNT=3.4
Males	106 (22.8%)	23 (8.8%)	83 (40.7%)	0.21 (0.14 – 0.33) p<0.0001 | NNT=3.1
Baseline Scores 3 and 4	1 (3.8%)	0 (0.0%)	1 (7.1%)	0.38 (0.02 – 8.65) p=1.00 | NNT = 16.3
Baseline Score 5	38 (15.0%)	5 (3.6%)	33 (28.4%)	0.13 (0.05 – 0.32) p<0.0001 | NNT=4.0
Baseline Score 6	133 (26.7%)	29 (10.6%)	104 (46.2%)	0.23 (0.16 – 0.33) p<0.0001 | NNT=2.8
All-cause mortality rate over 28 days, no. (%)	216 (27.8%)	45 (10.6%)	171 (48.2%)	0.22 (0.16 – 0.30) NNT = 2.7 | p < 0.0001
Females	87 (27.7%)	14 (8.6%)	73 (48.3%)	0.18 (0.10 – 0.30) p<0.0001 | NNT=2.5
Males	129 (27.8%)	31 (11.9%)	98 (48.0%)	0.25 (0.17 – 0.36) p<0.0001 | NNT=2.7
Baseline Scores 3 and 4	2 (7.7%)	0 (0.0%)	2 (14.3%)	0.23 (0.01 – 4.38) p=0.48 | NNT=7.8
Baseline Score 5	45 (17.8%)	6 (4.4%)	39 (33.6%)	0.13 (0.06 – 0.30) p<0.0001 | NNT=3.4
Baseline Score 6	169 (33.9%)	34 (14.2%)	130 (57.8%)	0.21 (0.15 – 0.30) p<0.0001 | NNT=2.2
Median hospitalization days (IQR)	10 (6-16)	8 (6-13)	12 (8-18)	p < 0.0001
Post-randomization time-to-discharge alive, median days (IQR)	7 (4-12)	5 (3-8)	9 (6-14)	p < 0.0001

Recovery rate over 14 days after randomization, as the primary outcome of this study, was 121% higher in the proxalutamide group (81.1%) than in the placebo group (36.6%) [Recovery ratio (RecR) 2.21; 95% confidence interval (95% CI), 1.92-2.56]. At Day 28, recovery rate was 81% higher in the proxalutamide group (86.1%) than in the placebo group (47.6%) (Rec, 1.81; 95% CI, 1.61-2.03). Median clinical score at Day 14 after randomization was significantly lower in the proxalutamide group [1; interquartile range (IQR), 1-2] than in the placebo group (6; IQR, 2-8) (p < 0.0001).

All-cause mortality rate at Day 14 was 879% lower in the proxalutamide group (8.0%) compared to the placebo group (38.9%) [Risk ratio (RR), 0.21; 95% CI, 0.15-0.30]. At Day 28, all-cause mortality ratio was 78% lower in the proxalutamide group (10.6%) than in the placebo group (48.2%) (RR, 0.22; 95% CI 0.16-0.30).

Hospitalization stay was shorter in the proxalutamide group (median, 8 days; IQR, 6-13) than in the placebo group (median, 12 days; IQR, 8-18) (p<0.0001). Conversely, post-randomization time-to-discharge alive from the hospital was shorter in the proxalutamide group (median, 5 days; IQR, 3-8) than in the placebo group (median, 9 days; IQR, 6-14) (p<0.0001).

**Figure 4:**
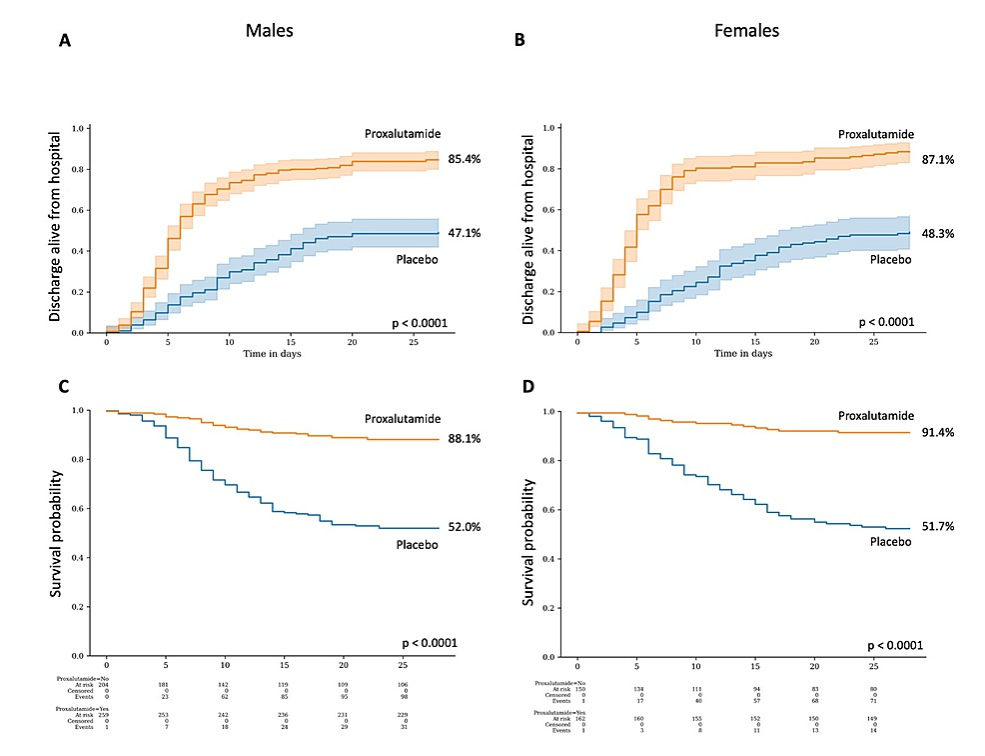
Proxalutamide group compared to placebo group stratified by sex. A. Proportion of patients discharged alive from the hospital in males; B. Proportion of patients discharged alive from the hospital in females; C. Kaplan-Meier survival probability in males; D. Kaplan-Meier survival probability in females.

Figure 4 illustrates the proportion of patients discharged alive in males (A) and in females (B), and Kaplan-Meier survival estimate of males (C) and females (D), when evaluated according to sex, recovery rate at Day 14 between males and females (115% and 131% higher recovery rate in the proxalutamide group, respectively), as well as at Day 28 (81% and 80%, respectively) (p<0.0001 between proxalutamide and placebo groups for all). All-cause mortality rates in males were similar to females at Day 14 (79% and 82% improvement, respectively) and at Day 28 (75% and 82% improvement, respectively) (p<0.0001 between proxalutamide and placebo groups for all).

**Figure 5:**
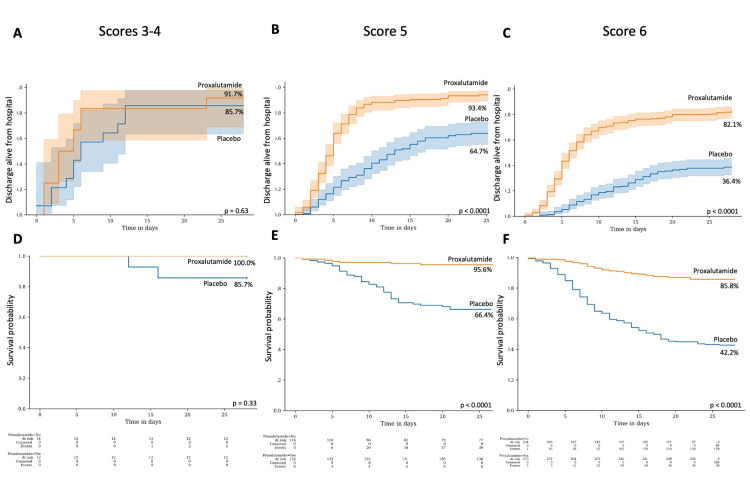
Proxalutamide group compared to placebo group stratified by baseline WHO COVID-19 ordinary score. A. Proportion of patients discharged alive from the hospital in subjects with baseline scores 3 and 4; B. Proportion of patients discharged alive from the hospital in subjects with baseline score 5; C. Proportion of patients discharged alive from the hospital in subjects with baseline score 6; D. Kaplan-Meier survival probability hospital in subjects with baseline scores 3 and 4; E. Kaplan-Meier survival probability hospital in subjects with baseline score 5; F. Kaplan-Meier survival probability hospital in subjects with baseline score 6.

Figure 5 shows the proportion of patients discharged from hospital alive (A, B and C) and the Kaplan-Meier survival estimate (D, E and F) according to the WHO COVID-19 Ordinary Clinical scores 3 and 4, score 5, and score 6, respectively, According to baseline clinical score, recovery rates at Days 14 and 28 were similar between proxalutamide and placebo groups at scores 3 and 4 (83.3% vs 85.7% at Day 14, p=0.87; 91.7% vs 85.7% at Day 28, p=0.63, respectively), while significantly higher in score 5 (89.8% vs 51.7% at Day 14 and 93.4% vs 64.7% at Day 28, p<0.0001 for both) and in score 6 (76.6% vs 25.8% at Day 14 and 82.1% vs 36.4% at Day 28, p<0.0001 for both). Improvement rates were relatively more remarkable among subjects at score 6, of 197% at Day 14 and 115% at Day 28. Mortality rates at Days 14 and 28 were similar between proxalutamide and placebo groups among subjects that were at scores 3 and 4 upon randomization (0% vs 7.1% at Day 14, p=0.63; 0% vs 14.3% at Day 28, p=0.33, respectively), whereas lower in proxalutamide group than in placebo group in score 5 (3.6% vs 28.4% at Day 14 and 4.4% vs 33.6% at Day 28, p<0.0001 for both) and in score 6 (10.6% vs 46.7% at Day 14 and 14.2% vs 57.8% at Day 28, p<0.0001 for both).

Table 3 describes the number and percentage of subjects that required the use of concurrent medications during the period of the study. All antibiotics, including cephalosporins (ceftriaxone, cefepime, cefuroxime), macrolides (azithromycin, clarithromycin), vancomycin, meropenem, and piperacillin/tazobactam, were used in a larger percentage of participants from the placebo group than from the proxalutamide group (p<0.0001 for all). Colchicine was also used in a larger percentage of patients from the placebo group (p<0.0001).

**Table 3:** Medications used during the period of the study.

Concomitant medications – no. (%)	Overall N=778	Proxalutamide N=423	Placebo N=355	p-value
Cephalosporins (Cefuroxime, Ceftriaxone, Cefepime)	659 (84.7%)	363 (85.8%)	340 (95.8%)	<0.0001
Colchicine	576 (74.0%)	255 (60.3%)	321 (90.4%)	<0.0001
Macrolides (azithromycin, clarithromycin)	694 (89.2%)	356 (84.2%)	338 (95.2%)	<0.0001
Glucocorticosteroids (dexamethasone, methylprednisolone)	778 (100.0%)	423 (100.0%)	355 (100.0%)	1.00
Enoxaparin	767 (98.6%)	414 (97.9%)	355 (100.0%)	0.003
Omeprazole	778 (100.0%)	423 (100.0%)	355 (100.0%)	1.00
Vancomycin	51/636 (8.0%)	3/307 (1.0%)	48/329 (14.6%)	<0.0001
Meropenem	97/636 (15.3%)	16/307 (5.2%)	81/329 (24.6%)	<0.0001
Piperacillin/Tazobactam	148/636 (23.3%)	24/307 (7.8%)	124/329 (37.7%)	<0.0001

Table 4 describes the treatment-emergent adverse effects (TEAEs). Proxalutamide group has a significantly lower percentage of subjects with at least one TEAE (p<0.0001), Grade 5 adverse effect (AE) (death) at Days 14 and 28 (p<0.0001 for both), and Grades 3 and 4 AEs, including shock requiring vasopressors (p<0.0001), mechanical ventilation at Day 14 (p<0.0001), renal failure (p=0.0008) and liver injury (p=0.003), while marginally lower in mechanical ventilation at Day 28 (p=0.068). All severe AEs (SAEs) were statistically lower in both males and females, except for liver damage in males (p=0.094) and mechanical ventilation at Day 14 (p=0.27 for males and p=0.14 for females). Among Grades 1 and 2 AEs, diarrhea was significantly more present in the proxalutamide group than in the placebo group (p<0.0001 for overall, p=0.0009 for females and p<0.0001 for males), while irritability and spontaneous erection were marginally higher among subjects in the proxalutamide group (p=0.056 and p=0.091, respectively).

**Table 4:** Adverse effects in overall and sex-stratified populations. TEAE = Treatment emergent adverse effect; n/a = non-applicable; ALT = Alanine transferase

Characteristic	Overall N=778 (females = 314) (males = 464)	Proxalutamide N=423 (females = 163) (males = 260)	Placebo N=355 (females = 151) (males = 204)	p-value
Number of subjects with 1 or more TEAE	387 (49.7%)	147 (34.7%)	240 (67.6%)	<0.0001
Grade 5 – n (%)				
Death, Day 14	172 (22.1%)	34 (8.0%)	138 (38.9%)	<0.0001
Females	66 (21.0%)	11 (6.7%)	55 (36.4%)	<0.0001
Males	107 (22.8%)	23 (8.8%)	83 (40.7%)	<0.0001
Death, Day 28	216 (27.8%)	40 (9.5%)	171 (48.2%)	<0.0001
Females	87 (27.7%)	10 (6.1%)	73 (48.3%)	<0.0001
Males	129 (27.8%)	30 (11.5%)	98 (48.0%)	<0.0001
Grades 4 or 3 – n (%)				
Shock, requiring vasopressors	227 (29.2%)	49 (11.6%)	178 (50.1%)	<0.0001
Mechanical ventilation, Day 14	47 (6.0%)	7 (1.6%)	40 (11.3%)	<0.0001
Females	23 (7.3%)	3 (1.8%)	20 (13.2%)	0.001
Males	24 (5.2%)	4 (1.5%)	20 (9.8%)	0.0006
Mechanical ventilation, Day 28	7 (0.9%)	1 (0.2%)	6 (1.7%)	0.068
Females	2 (0.6%)	0 (0.0%)	2 (1.3%)	0.27
Males	5 (1.1%)	1 (0.4%)	4 (2.0%)	0.14
Disease progression	235 (30.2%)	53 (12.5%)	182 (51.3%)	<0.0001
Females	93 (29.6%)	15 (9.2%)	78 (51.7%)	<0.0001
Males	142 (30.6%)	38 (14.6%)	104 (51.0%)	<0.0001
Renal failure (creatinine increase > 100%)	29 (3.7%)	6 (1.4%)	23 (6.5%)	0.0002
Females	12 (3.8%)	1 (0.6%)	11 (7.3%)	0.002
Males	17 (3.7%)	5 (1.9%)	12 (5.9%)	0.043
Liver damage (ALT > 250 U/L or >100% increase)	30 (3.9%)	8 (1.9%)	22 (6.2%)	0.002
Females	11 (3.5%)	1 (0.6%)	10 (6.6%)	0.004
Males	19 (4.1%)	7 (2.7%)	12 (5.9%)	0.10
Grades 2 or 1 – n (%)				
Diarrhea	89 (11.4%)	77 (18.2%)	12 (3.4%)	<0.0001
Females	36 (11.5%)	29 (17.8%)	7 (4.6%)	0.0003
Males	53 (11.4%)	48 (18.5%)	5 (2.5%)	<0.0001
Abdominal pain	7 (0.9%)	5 (1.2%)	2 (0.6%)	0.46
Females	1 (0.3%)	1 (0.6%)	0 (0.0%)	1.00
Males	6 (1.3%)	4 (1.5%)	2 (1.0%)	0.70
Irritability	9 (1.2%)	9 (2.1%)	0 (0.0%)	0.005
Females	1 (0.3%)	1 (0.6%)	0 (0.0%)	1.00
Males	8 (1.7%)	8 (3.1%)	0 (0.0%)	0.01
Spontaneous erection (males)	7 (1.5%)	7 (2.7%)	0 (0.0%)	0.02
Vomiting, dyspepsia, or palpitations	0 (0.0%)	0 (0.0%)	0 (0.0%)	n/a

North versus South

Table 5 compared the baseline characteristics of the North arm of the Proxa-Rescue Trial and the South arm of the same trial. Participants of the South arm were older (median age, 55 y/o vs 50 y/o in the North arm, p=0.0004; 50.4% above 55 y/o versus 36.9% in the North arm, p=0.003), had more males (73.7% vs 56.7% in the North arm, p=0.001), fewer previously healthy (no comorbidities) subjects (55.6% vs 66.8% in the North arm, p=0.026), lower time from hospitalization to randomization (p<0.0001), and lower percentage of participants in severe state (Score 6) at baseline (53.4% vs 66.5% in the North arm, p=0.01).

**Table 5:** Baseline characteristics in the North arm versus the South arm. IQR = Interquartile range; n/a = non-applicable

Characteristic	South N=133	North N=645	p-value
Age			
Median – years (IQR)	55 (46-63)	50 (40-61)	<0.0001
> 55 yr – no. (%)	67 (50.4%)	238 (36.9%)	0.003
Sex – no. (%)			0.001
Female	35 (26.3%)	279 (43.3%)	
Male	98 (73.7%)	366 (56.7%)	
Comorbidities			
Body mass index over 30 kg/m^2^ – no. (%)	15 (11.3%)	53 (8.2%)	0.24
Hypertension – no. (%)	40 (30.1%)	175 (27.1%)	0.52
Type 2 diabetes mellitus – no. (%)	18 (13.5%)	79 (12.2%)	0.27
Chronic obstructive pulmonary disorder – no. (%)	2 (1.5%)	16 (2.5%)	0.75
Chronic kidney disease – no. (%)	0 (0.0%)	0 (0.0%)	n/a
Coexisting conditions – no. (%)			
0	74 (55.6%)	431 (66.8%)	0.026
1	34 (25.6%)	124 (19.2%)	0.091
2+	25 (18.8%)	90 (14.0%)	0.15
Median time from hospitalization to randomization (IQR) – days	1.0 (1.0-3.0)	2.0 (1.0-4.0)	<0.0001
Score on the Coronavirus Disease 2019 ordinal scale – no. (%)			
4. Hospitalized, not requiring supplemental oxygen, requiring ongoing medical care (COVID-19 related or otherwise)	6 (4.5%)	20 (3.1%)	0.41
5. Hospitalized, requiring supplemental oxygen	56 (42.1%)	196 (30.4%)	0.006
6. Hospitalized, receiving non-invasive ventilation or high flow oxygen devices	71 (53.4%)	429 (66.5%)	0.01
Concomitant medications – no. (%)			
Cephalosporins (Cefuroxime, Ceftriaxone, Cefepime)	118 (88.7%)	638 (98.9%)	0.0005
Colchicine	69 (51.9%)	407 (63.1%)	0.027
Macrolides (azithromycin, clarithromycin)	61 (45.9%)	631 (97.8%)	<0.0001
Glucocorticosteroids (dexamethasone, methylprednisolone)	133 (100.0%)	645 (100.0%)	1.00
Enoxaparin	122 (91.7%)	645 (100.0%)	0.0009
Omeprazole	133 (100.0%)	645 (100.0%)	1.00

Participants in the South arm used less antibiotics, including cephalosporins (88.7% vs 98.9% in the North arm, p=0.0005) and macrolides (45.9% vs 97.8% in the North arm, p<0.0001). Patients in the South arm also used less enoxaparin (91.7% vs 100% in the North arm, p=0.0009) and colchicine (51.9% vs 63.1% in the North arm, p=0.027). All patients from both arms used glucocorticoids and omeprazole.

**Figure 6:**
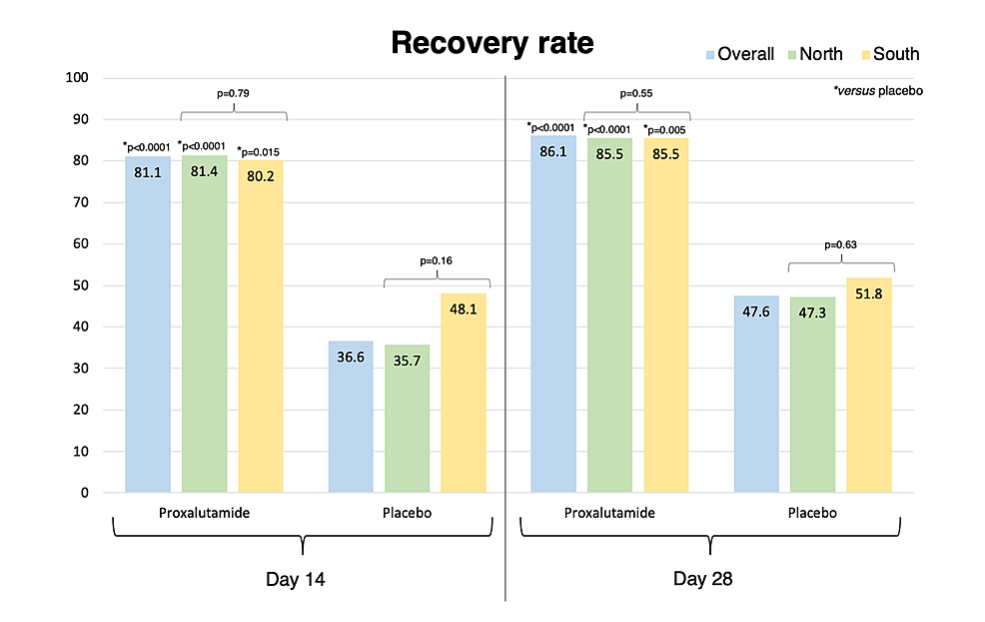
Recovery rates at Day 14 and Day 28 in proxalutamide and in placebo group in overall population (North and South arms combined), in the North arm, and in the South arm.

**Figure 7:**
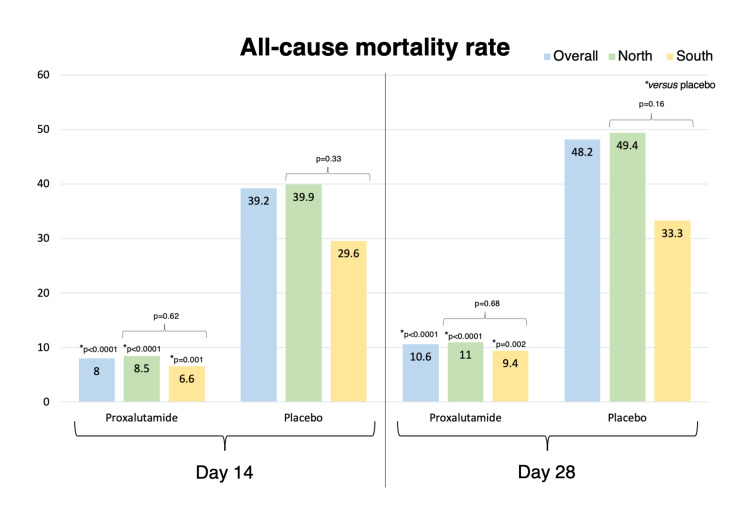
Mortality rates at Day 14 and Day 28 in proxalutamide and placebo group in overall population (North and South arms combined), in the North arm, and in the South arm.

Table 6 compares results between proxalutamide groups and between placebo groups from the South and the North arm. Figure 6 and Figure 7 illustrate, respectively, recovery rates and mortality rates in the North arm, South arm, and overall population. Recovery rate at Days 14 and 28, and mortality rates at Days 14 and 28 were statistically similar between proxalutamide groups and between placebo groups. Hospitalization stay was slightly longer in the active group in the North compared to the South (p=0.05) while was lower in the placebo group in the North compared to the South (p=0.045). Post-randomization length of hospital stay was significantly shorter in the placebo group of the North arm (median, 10 days; IQR, 6-15) than the placebo group of the South arm (median, 12 days; IQR, 9-15) (p=0.005).

**Table 6:** Outcomes in active and placebo groups in the North compared to the South arm. IQR = Interquartile range

Characteristic	South Active N=106 Male (n=76) Female (n=30) (sc. 3/4 = 5) (sc. 5 = 43) (sc. 6 = 58)	North Active N=317 Male (n=184) Female (n=133) (sc. 3/4 = 7) (sc. 5 = 93) (sc. 6 = 217)	South Placebo N=27 Male (n=22) Female (n=5) (sc. 3/4 = 1) (sc. 5 = 13) (sc. 6 = 13)	North Placebo N=328 Male (n=182) Female (n=146) (sc. 3/4 = 13) (sc. 5 = 103) (sc. 6 = 212)	Active between regions (P-value)	Placebo between regions (P-value)
Day 14 WHO COVID-19 Clinical Score – median (IQR)	1 (1-2)	1 (1-2)	4 (2-8)	7 (2-8)	0.81	0.27
Day 18 WHO COVID-19 Clinical Score – median (IQR)	1 (1-1)	1 (1-1)	2 (1-8)	7 (2-8)	0.60	0.23
Recovery rate over 14 days, no. (%)	85 (80.2%)	258 (81.4%)	13 (48.1%)	117 (35.7%)	0.79	0.16
Females	24 (80.0%)	109 (82.0%)	1 (20.0%)	51 (34.9%)	0.81	0.54
Males	63 (82.9%)	149 (81.0%)	9 (40.9%)	66 (36.3%)	0.71	0.66
Baseline Scores 3 and 4	3 (60.0%)	7 (100.0%)	1 (100%)	11 (84.6%)	0.16	0.16
Baseline Score 5	41 (95.3%)	82 (88.2%)	9 (69.2%)	51 (49.5%)	0.12	0.11
Baseline Score 6	41 (70.7%)	169 (77.9%)	3 (23.1%)	55 (25.9%)	0.27	0.82
Recovery rate over 28 days no. (%)	93 (87.7%)	271 (85.5%)	14 (51.8%)	155 (47.3%)	0.55	0.63
Females	27 (90.0%)	117 (88.0%)	2 (40.0%)	71 (48.6%)	0.74	0.72
Males	68 (89.5%)	154 (83.7%)	14 (63.6%)	84 (46.2%)	0.19	0.074
Baseline Scores 3 and 4	4 (80.0%)	7 (100.0%)	1 (100%)	11 (84.6%)	0.32	0.16
Baseline Score 5	43 (100.0%)	86 (92.5%)	9 (69.2%)	66 (64.1%)	0.008	0.39
Baseline Score 6	47 (81.0%)	178 (82.0%)	4 (30.8%)	78 (36.8%)	0.81	0.67
All-cause mortality rate over 14 days, no. (%)	7 (6.6%)	27 (8.5%)	8 (29.6%)	130 (39.6%)	0.68	0.34
Females	0 (0.0%)	11 (8.3%)	2 (40.0%)	53 (36.3%)	0.22	1.00
Males	7 (9.2%)	16 (8.7%)	6 (27.3%)	77 (42.3%)	1.00	0.52
Baseline Scores 3 and 4	0 (0.0%)	0 (0.0%)	0 (0.0%)	1 (7.7%)	1.00	1.00
Baseline Score 5	1 (2.3%)	4 (4.3%)	2 (15.4%)	31 (30.1%)	1.00	0.34
Baseline Score 6	6 (10.3%)	23 (10.6%)	6 (46.2%)	98 (46.2%)	1.00	1.00
All-cause mortality rate over 28 days, no. (%)	10 (9.4%)	35 (11.0%)	9 (33.3%)	162 (49.4%)	0.72	0.11
Females	2 (6.7%)	12 (9.0%)	2 (40.0%)	71 (48.6%)	1.00	1.00
Males	8 (10.5%)	23 (12.5%)	7 (31.8%)	91 (50.0%)	0.83	0.12
Baseline Scores 3 and 4	0 (0.0%)	0 (0.0%)	0 (0.0%)	2 (15.4%)	1.00	1.00
Baseline Score 5	1 (2.3%)	5 (5.4%)	2 (15.4%)	37 (35.9%)	0.66	0.21
Baseline Score 6	9 (15.5%)	30 (13.8%)	7 (53.8%)	123 (58.0%)	0.83	0.78
Median hospitalization days (IQR)	8 (5-10)	8 (6-13)	14 (12-18)	12 (8-18)	0.07	0.12
Post-randomization to alive hospital discharge, median days (IQR)	5 (3-8)	5 (3-8)	12 (9-16)	10 (6-14)	1.00	0.025

Table 7 describes the differences of each outcome between the active and placebo groups, translated as estimated number of patients that would have changed the outcome if all participants were from the same group, in the South and in the North arm. The difference in the recovery rate at Day 14 between proxalutamide and placebo arms was significantly higher in the North than in the South arm (p=0.009), while was similar at Day 28 (p=0.66). However, the difference in recovery rate at Day 28 in males was higher in the North than in the South arm (p=0.039). The difference in all-cause mortality between proxalutamide and placebo groups at Day 14 was marginally higher in the North than in the South arm (p=0.073) and significantly higher at Day 28 (p=0.004). Among females, there were no differences between the North and South arm (p=0.12 at Day 14 and p=0.34 at Day 28), whereas males had significantly more benefit of survival in the North arm compared to the South arm at both Days 14 (p=0.004) and 28 (p=0.006).

**Table 7:** Differences between active and placebo groups in the South arm and in the North arm. 95%CI = 95% confidence interval

Difference (expected difference in terms of no. of patients / overall no. of patients)	Difference between Active and Placebo Arms (South)	Difference between Active and Placebo Arms (North)	South Risk ratio (95% CI)	North Risk ratio (95% CI)	Difference between responses in the North vs South (P-value)
Recovery rate over 14 days, no. (%)	42.61/133	294.83/645	1.67 (1.11 – 2.49)	2.28 (1.95 – 2.66)	0.009
Females	21.00/35	131.13/279	4.00 (0.69 – 23.3)	2.35 (1.85 – 2.97)	0.11
Males	41.14/98	163.67/366	2.03 (1.21 – 3.88)	2.23 (1.82 – 2.74)	0.60
Baseline Scores 3 and 4	2.40/6	3.22/20	0.60 (0.29 – 1.23)	1.22 (0.93 – 1.61)	0.31
Baseline Score 5	14.62/56	73.52/197	1.41 (0.98 – 2.03)	1.78 (1.45 – 2.19)	0.18
Baseline Score 6	33.80/71	223.84/429	3.01 (1.10 – 8.24)	3.00 (2.37 – 3.81)	0.50
Recovery rate over 28 days no. (%)	47.73/133	246.58/645	1.69 (1.17 – 2.45)	1.81 (1.60 – 2.05)	0.66
Females	17.5/35	109.76/279	2.25 (0.76 – 6.63)	1.81 (1.51 – 2.16)	0.27
Males	25.31/98	137.43/366	1.41 (1.02 – 1.94)	1.81 (1.53 – 2.15)	0.039
Baseline Scores 3 and 4	1.20/6	3.18/20	0.80 (0.52 – 1.24)	1.22 (0.93 – 1.61)	0.82
Baseline Score 5	15.93/56	53.98/197	1.44 (1.01 – 2.08)	1.44 (1.23 – 1.69)	0.86
Baseline Score 6	35.68/71	195.25/429	2.24 (1.86 – 2.70)	2.32 (1.85 – 2.68)	0.40
All-cause mortality rate over 14 days, no. (%)	30.63/133	195.56/645	0.22 (0.09 – 0.56)	0.17 (0.11 – 0.27)	0.073
Females	14.00/35	78.20/279	0.04 (0.00 – 0.71)	0.14 (0.07 – 0.31)	0.12
Males	17.70/98	123,02/66	0.34 (0.13 – 0.9)	0.19 (0.11 – 0.32)	0.006
Baseline Scores 3 and 4	0.00/6	1.54/20	0.33 (0.01 – 11.9)	0.58 (0.03 – 12.7)	0.98
Baseline Score 5	7.31/56	50.92/197	0.15 (0.02 – 1.57)	0.14 (0.05 – 0.38)	0.089
Baseline Score 6	25.42/71	152.85/429	0.22 (0.08 – 0.57)	0.23 (0.15 – 0.34)	0.90
All-cause mortality rate over 28 days, no. (%)	31.79/133	247.03/645	0.28 (0.13 – 0.63)	0.22 (0.16 – 0.31)	0.004
Females	11.67/35	110.51/279	0.17 (0.03 – 0.93)	0.18 (0.11 – 0.33)	0.54
Males	20.86/98	137.25/366	0.33 (0.13 – 0.81)	0.25 (0.17 – 0.38)	0.006
Baseline Scores 3 and 4	0.00/6	3.08/20	0.33 (0.01 – 11.9)	0.30 (0.02 – 5.45)	0.56
Baseline Score 5	7.31/56	48.82/197	0.15 (0.02 – 1.57)	0.15 (0.06 – 0.36)	0.13
Baseline Score 6	27.21/71	188.88/429	0.28 (0.13 – 0.62)	0.24 (0.17 – 0.34)	0.71

As shown in Table 8, there were no significant differences between proxalutamide groups and between placebo groups in SAEs (grades 3 to 5), except for a marginally increased chance of disease progression in females of the placebo group from the South arm (80.0%) compared to the North arm (50.7%) (p=0.055). Diarrhea was more commonly present among participants of the proxalutamide group from the South arm (24.5%) than in the North arm (16.1%) (p=0.048). Irritability and spontaneous erection were more commonly observed among overall and males of the proxalutamide group from the South arm (4.7% and 6.6%, respectively) than from the North arm (1.3% and 1.6%, respectively (p=0.046 and p=0.052, respectively).

**Table 8:** Adverse effects in proxalutamide and placebo groups in the South arm and in the North arm. TEAE = Treatment emergent adverse effect; n/a = non-applicable; ALT = Alanine transferase

Adverse effect	South Active N=106 Male (n=76) Female (n=30)	North Active N=317 Male (n=184) Female (n=133)	South Placebo N=27 Male (n=22) Female (n=5)	North Placebo N=328 Male (n=182) Female (n=146)	South between active versus placebo (p-value)	North between active versus placebo (p-value)	Active between regions (p-value)	Placebo between regions (p-value)
Number of subjects with 1 or more TEAE	38 (35.8%)	109 (34.4%)	15 (55.5%)	225 (68.6%)	0.042	<0.001	0.78	0.23
Grade 5 – n (%)								
Death, Day 14	7 (6.6%)	27 (8.5%)	8 (29.6%)	130 (39.6%)	0.001	<0.001	0.68	0.34
Females	7 (7.9%)	11 (8.3%)	6 (27.3%)	53 (36.3%)	0.03	<0.001	0.22	1.00
Males	0 (0.0%)	16 (8.7%)	2 (40.0%)	77 (42.3%)	0.028	<0.001	1.00	0.52
Death, Day 28	10 (9.4%)	35 (11.0%)	9 (33.3%)	162 (49.4%)	0.002	<0.001	0.72	0.11
Females	8 (10.5%)	12 (9.0%)	7 (31.8%)	71 (48.6%)	0.016	<0.001	1.00	1.00
Males	2 (6.7%)	23 (12.5%)	2 (40.0%)	91 (50.0%)	0.041	<0.001	0.83	0.12
Grades 4 or 3 – n (%)								
Shock, requiring vasopressors	12 (11.3%)	37 (11.7%)	10 (37.0%)	168 (51.2%)	0.001	<0.001	0.92	0.21
Mechanical ventilation, Day 14	3 (2.8%)	4 (1.3%)	4 (14.8%)	37 (11.3%)	0.024	0.038	0.29	0.58
Females	2 (6.7%)	1 (0.8%)	1 (20.0%)	20 (13.7%)	0.42	0.089	0.071	0.83
Males	1 (1.3%)	3 (1.6%)	3 (13.6%)	17 (9.3%)	0.038	0.28	0.85	0.52
Mechanical ventilation, Day 28	0	5 (1.6%)	2 (7.4%)	41 (12.5%)	0.055	<0.001	0.37	0.71
Females	0	1 (0.8%)	0	22 (15.1%)	n/a	<0.001	0.82	0.66
Males	0	4 (2.2%)	2 (9.1%)	19 (10.4%)	0.066	0.001	0.37	0.85
Disease progression	17 (16.0%)	36 (11.4%)	14 (51.9%)	168 (51.2%)	0.0001	<0.001	0.20	0.95
Females	3 (10.0%)	12 (9.0%)	4 (80.0%)	74 (50.7%)	0.0009	<0.001	0.87	0.055
Males	14 (18.4%)	24 (13.0%)	10 (45.5%)	94 (51.6%)	0.007	<0.001	0.26	0.60
Renal failure (creatinine increase > 100%)	1 (0.9%)	5 (1.6%)	2 (7.4%)	21 (6.4%)	0.087	0.29	1.00	0.69
Females	0 (0.0%)	1 (0.8%)	0 (0.0%)	11 (7.5%)	n/a	0.33	1.00	1.00
Males	1 (1.3%)	4 (2.2%)	2 (9.1%)	10 (5.5%)	0.11	0.58	1.00	0.62
Liver damage (ALT > 250 U/L or >100% increase)	4 (3.8%)	4 (1.3%)	3 (11.1%)	19 (5.8%)	0.14	0.32	0.11	0.23
Females	1 (3.3%)	0 (0.0%)	0 (0.0%)	10 (6.8%)	0.73	0.37	0.18	1.00
Males	3 (3.9%)	4 (2.2%)	3 (13.6%)	9 (4.9%)	0.11	0.65	0.42	0.20
Grades 2 or 1 – n (%)								
Diarrhea	26 (24.5%)	51 (16.1%)	1 (3.7%)	11 (3.4%)	0.058	0.005	0.059	1.00
Females	7 (23.3%)	22 (16.5%)	0 (0.0%)	7 (4.8%)	0.44	0.091	0.43	1.00
Males	19 (25.0%)	29 (15.8%)	1 (4.5%)	4 (2.2%)	0.087	0.025	0.11	0.44
Abdominal pain	2 (1.9%)	3 (0.9%)	1 (3.7%)	1 (0.3%)	0.58	0.89	0.60	0.15
Females	0 (0.0%)	1 (0.8%)	0 (0.0%)	0 (0.0%)	n/a	0.91	1.00	n/a
Males	2 (2.6%)	2 (1.1%)	1 (4.5%)	1 (0.5%)	0.65	0.93	0.58	0.20
Irritability	5 (4.7%)	4 (1.3%)	0 (0.0%)	0 (0.0%)	0.47	0.78	0.048	n/a
Females	0 (0.0%)	1 (0.8%)	0 (0.0%)	0 (0.0%)	n/a	0.91	1.00	n/a
Males	5 (6.6%)	3 (1.6%)	0 (0.0%)	0 (0.0%)	0.41	0.79	0.049	n/a
Spontaneous erection (Males)	3 (3.9%)	4 (2.2%)	0 (0.0%)	0 (0.0%)	0.62	0.73	0.42	n/a
Vomiting, dyspepsia, or palpitations	0 (0.0%)	0 (0.0%)	0 (0.0%)	0 (0.0%)	n/a	n/a	n/a	n/a

## Discussion

This final, joint analysis of the Proxa-Rescue Trial including both South and North arms with proxalutamide for hospitalized COVID-19 patients demonstrated that proxalutamide is effective for COVID-19 in later stages of the disease and that benefits of proxalutamide for this population were consistent across different regions with distinct demographical characteristics.

In the South as in the North: successful reproduction of the findings from the Proxa-Rescue AndroCoV trial

The fact that this RCT demonstrated a large efficacy of proxalutamide in patients hospitalized due to COVID-19 in Northern Brazil, we had to address the question of whether these findings would be reproducible in a population with distinct characteristics in a different region.

The important improvements in clinical outcomes [30], including reductions in mortality rate and hospital length stay and increase in recovery speed rate, observed in the first study on hospitalized patients, were replicated in the Proxa-South Rescue Trial [31].

The recovery rate ratio was similar at Day 14, of 2.28 (95%CI, 1.95 - 2.66) in the North and 2.22 (95%CI, 1.34 - 3.65) in the South. At Day 28, the recovery rate ratio was slightly lower in the South (1.51; 95%CI, 1.10 - 2.08) than in the North (1.81; 95%CI, 1.60 - 2.05). The speed of recovery rate at Day 14, the primary objective of these RCTs, showed that proxalutamide led to an increase of approximately 120% to 130% in speed recovery, quite similar between groups.

The all-cause mortality rate ratio between the proxalutamide arm and the placebo arm at Day 14 was similar between studies: 0.22 (95%CI, 0.09 - 0.56) in the South arm and 0.21 (95%CI, 0.14 - 0.32) in the North arm. At Day 28, all-cause mortality ratio rate was slightly higher in the South arm (0.28; 95%CI, 0.13 - 0.63) than in the North arm (0.22; 95%CI 0.16 - 0.31). The reduction of all-cause mortality at both Days 14 and 28 was between 78% and 72%, almost identical between the studies in the North and in the South.

Overall and post-randomization hospital stay until discharge in the placebo group was shorter in the North arm (Overall hospital stay: median, 12 days; 95%CI, 9 - 16; post-randomization hospital stay: median: 10 days; 95%CI, 6 - 14) than in the South arm (Overall hospital stay: median, 14 days; 95%CI, 12 - 18; post-randomization hospital stay: median: 12 days; 95%CI, 8 - 18) (p = 0.12 and 0.025, respectively). This is possibly resulted from the increased need to discharge patients from the hospitals in the North arm, due to the collapsed situation in hospitals, which was worse in Northern than Southern Brazil.

The sole non-severe TEAE more commonly present among patients from the proxalutamide group was diarrhea, which corresponds to the same sole TEAE reported in the study conducted in the North.

In the North arm, patients were younger, had fewer males and had higher proportion of subjects without comorbidities than in the South arm. This is in accordance with the regional demographic between regions in Brazil [35]. In the case of higher prevalence of male sex in the South arm, approximately 45% of the participants were originally from a military hospital, where the vast majority of patients are males. Conversely, there were approximately 20% more patients in severe state (score 6) the in North arm than in the South arm (p<0.001). While patients in Southern Brazil had a higher risk of dying from COVID-19, patients in Northern Brazil presented more severe COVID-19 states, possibly due to the collapse in the overall health system in the state of Amazonas during the trial, which hampered non-severe COVID-19 patients from being hospitalized, even fulfilling criteria for hospitalization.

The higher percentage of participants that used antibiotics in the North arm compared to the South arm may reflect the higher proportion of patients in the placebo group in this arm (1:1) than in the South arm (4:1), and the higher percentage of severe patients in the North arm compared to the South.

Recovery and mortality rates presented better results in the North than in the South arm: recovery rate 128% versus 67% higher at Day 14 (p=0.009) and 81% versus 51% at Day 28 (non-significantly; p=0.66), and all-cause mortality rate 83% versus 78% lower at Day 14 (marginally significant; p=0.073) and 78% versus 72% at Day 28 (p=0.004). The difference in the size of efficacy, which shows a more prominent response with proxalutamide in the North arm, can be fully explained by the higher proportion of patients in score 6 in the North (approximately two thirds) than in the South arm (approximately half of participants), since when stratified by baseline score, all differences vanish.

As mentioned, compared to Northern Brazil, hospitals in Southern Brazil were slightly less, although overwhelmingly occupied. As a result, criteria for hospitalization were adapted to encompass patients that needed medical assistance most in both regions, but patients at a higher severity were to be hospitalized in the North than in the South, which explains the differences in the proportion of patients in score 6.

Differences in mortality rate in the placebo group between Northern and Southern Brazil were non-significant and reflected regional differences observed in Brazil [34,35]. In both studies, the all-cause mortality rate in the placebo groups was smaller or similar to the COVID-19 in-hospital mortality rate in the respective regions [34] - of approximately 50% in the North arm and 35% in the South arm [35].

Overall, results were exceptionally similar between the populations in the North and in the South, despite the slight demographic differences, indicating a high consistency of the findings when validated externally within the study. Of note, in both cases, P.1 (gamma) variant was the cause of COVID-19 in virtually all patients, and standard of care was in general similar between all hospitals.

The fact that comparisons between active groups and between placebo groups in all major outcomes showed similar responses between regions reinforces the efficacy of proxalutamide in terms of recovery and mortality rates.

Overall results

When analyzed for overall participants from the proxalutamide group were marginally older than participants from the placebo group because, as mentioned, in the South, where the randomization ratio was 4:1, patients were older than patients in the North. Conversely, older patients tend to present more comorbidities, and T2DM was marginally more present in the proxalutamide group. This is a conservative bias that may underestimate the efficacy of proxalutamide.

Disease progression occurred approximately 4.1 times more frequently in the placebo group than in the proxalutamide group, while progression to shock requiring vasopressors was 4.4 times more frequent in the placebo group (p<0.0001 for both). Accordingly, the proportion of patients in the placebo group with renal injury and liver damage was 4.5 and 3.2 times higher than patients in the proxalutamide group (p=0.0008 and p=0.003, respectively). The remarkable reduction in disease progression and liver and kidney injuries reinforces the efficacy of proxalutamide to interrupt the COVID-19 disease course during the second and third phases of the disease. The resulted reduction in mortality rate likely reflects not only the improvement of COVID-19 per se, but also the numerous secondary complications caused by the progression of the disease.

Reduction in hospitalization stay was 33.3% (p<0.0001) with proxalutamide, particularly when proxalutamide is initiated earlier in hospitalization, since post-randomization time-to-discharge alive from the hospital was almost 50% lower in the proxalutamide group than in the placebo group (p<0.0001). This is particularly important during outbreaks and collapses of hospitals, when earlier discharge may allow undertreated patients to be hospitalized.

**Figure 8:**
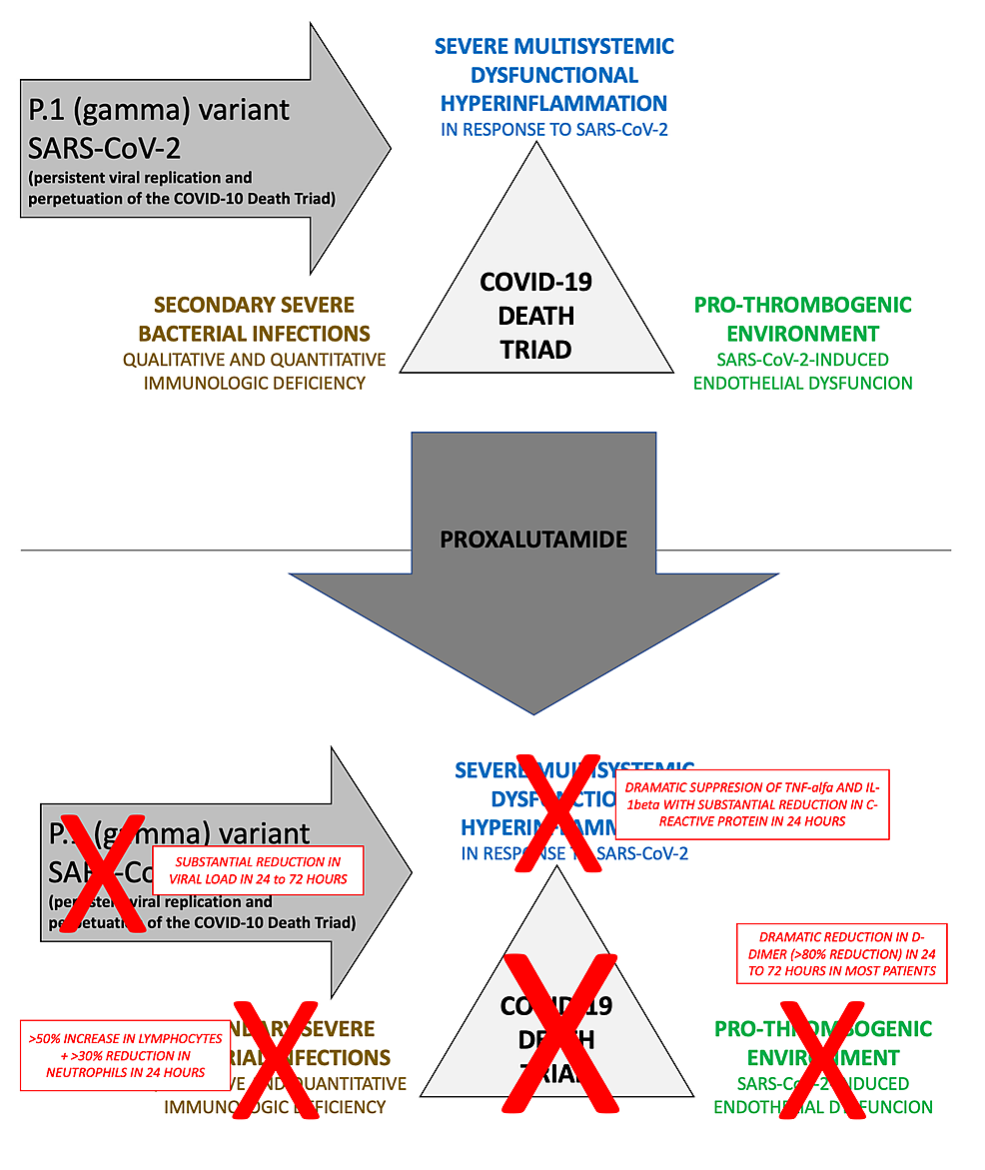
Proposed mechanisms for proxalutamide against late-stage COVID-19.

The larger proportion of patients from the placebo arm that used different classes of antibiotics reflect the progression of the disease, with deterioration of the pulmonary status and occurrence of secondary bacterial infections, that occurred more commonly in the placebo group. As reinforced by the lower use of antibiotics, proxalutamide may have prevented the occurrence of secondary bacterial infection, commonly seen in severe COVID-19. Proxalutamide also demonstrated theoretical reduction of the COVID-19-triggered endothelial dysfunction, observed through substantial decrease in D-dimer levels. To which extent anti-inflammatory, anti-thrombotic and immunomodulatory effects of proxalutamide were directly mediated or indirectly induced by reduction in viral load is not clarified. The prevailing SARS-CoV-2 variant during the course of the trials was the P.1 (gamma), which, unlike other variants, seemed to present persistent viral activity after seven to 14 days of the disease, as observed through the persistent, unexplained and refractory increase of inflammatory and thrombotic markers during hospitalization [36-39]. Figure 8 summarizes the critical mechanisms in late-stage COVID-19 that lead to death, and the proposed mechanisms of protection conferred by proxalutamide.

The unexpected spontaneous prolonged erection among males in the proxalutamide group may have occurred due to a short-term rise in testosterone levels, as seen in our previous study in male outpatients [29].

In females as in males: an unexpected balance in the results

While it would be expected that males would present a more substantial improvement in clinical outcomes due to the higher circulating testosterone and dihydrotestosterone (DHT) levels compared to women, reduction in overall mortality rate and increase in recovery rate with proxalutamide were unexpectedly observed both in males and females. Responses not only occurred in a non-gender specific manner but were more marked in women in both recovery rate ratio at Day 14 (4.0 vs 2.5 in men) and at Day 28 (2.2 vs 1.4 in men) and mortality risk ratio at Day 14 (96% reduction vs 66% reduction in men) and at Day 28 (83% reduction vs 67% reduction in men). This finding is consistent with the findings in the North arm, where both males and females responded similarly to proxalutamide, when compared to the placebo arm.

The unexpected response in women may be justified by the fact that although females have lower androgen circulating levels, they tend to be more sensitive to androgens due to potential higher sensitivity of the androgen receptor (AR) and inherently upregulation of the AR, leading to increased density of AR in the cytoplasm. The hyperresponsiveness to androgens in a manner that slightly increased testosterone levels may be sufficient to become an independent risk factor for severe COVID-19 [40]. Consequently, antagonism of AR or blockage of more active androgen hormones may lead to more prominent responses.

Some of the differences between females of the placebo groups can be justified by the small number of participants in the group from the South arm. This peculiarity should be considered in comparisons that encompass female placebo groups.

The level of COVID-19 severity as a predictor of clinical response to proxalutamide

The improvement of recovery rate at Days 14 and 28 was higher in score 6 (196% and 114%, respectively) than score 5 (75% and 46%, respectively) or scores 3 and 4 (-3% and 7%, respectively). However, reduction in mortality rate at Days 14 and 28 was slightly higher in moderate (Score 5) patients (87% for both) than score 6 (77% and 79%, respectively) or score 4 (62% and 77%, respectively).

The importance of the analysis according to the baseline clinical score is to predict subjects that should present better responses. From this analysis, apparently the patients that are most benefited from proxalutamide are those that required oxygen supplement but still not needing higher oxygen flow or additional non-invasive respiratory devices, i.e., in score 5, and in any case when oxygen is needed (scores 5 and 6) compared to cases when oxygen is not needed (scores 3 and 4).

The P.1 (Gamma) SARS-CoV-2 variant and its implications in the efficacy of proxalutamide

The P.1 (Gamma) variant of SARS-CoV-2 is a variant of concern (VOC) that, although not officially recognized, is a strong candidate to be considered the first variant of high consequence (VOHC) [36], since it has been demonstrated to lead to more severe clinical disease, increased hospitalization and increased lethality, with an up to 4-time higher risk of in-hospital death compared to the B.1.617.2/AY lineages (Delta) variant and to present increased transmissibility and reduction in the efficacy and response of the therapies against COVID-19 [37,38]. Since vaccination rates were virtually null by the time of the peak of P.1 variant, the efficacy of the vaccines to prevent infection or attenuate clinical disease for the P.1 variant is unclear.

To our knowledge, proxalutamide was the first drug tested in hospitalized patients due to COVID-19 in the P.1 (Gamma) variant, demonstrated not by the fact that the P.1 variant was present in more than 90% of tested subjects in the regions where the RCTs were conducted during the period of the studies, but essentially because the prevalence of P.1 was 100% of patients that participated in the RCTs and underwent SARS-CoV-2 genotyping presented the P.1 (Gamma) variant [30,39]. Moreover, in the city of Gramado, in Southern Brazil, we discovered the first cases of the P.1.2 subvariant, which could also interfere in the clinical course and response to proxalutamide, in addition to the changes that could have been caused by the P.1 variant, compared to RCTs conducted in prior variants [39].

The P.1 variant of the SARS-CoV-2 is highly dependent on TMPRSS2 for its cell entry, and induces dramatic dysfunctional inflammatory and immunologic responses as well as a highly pro-thrombogenic endothelial dysfunction. While a reduction in the efficacy of proxalutamide could be expected due to the P.1 variant's typical resistance to usual care for COVID-19, since all the three major aspects of the P.1 variant (high dependence on TMPRSS2 for cell entry, dysfunctional exacerbated inflammatory and immunologic reactions, and severe endothelial dysfunction) seem to be specially targeted by an anti-androgen [41,42], the high efficacy of proxalutamide in the P.1 variant could also be justified by these peculiarities of the variant present in the RCTs. However, enzalutamide, a high-potent drug from the same drug class as proxalutamide although with weaker antiandrogen activity, demonstrated to dramatically reduce SARS-CoV-2 cell entry in the lung by downregulating TMPRS22, in a non-P.1 SARS-CoV-2 variant [43].

Scientific aspects of studies on COVID-19

Some noteworthy particularities of the research on COVID-19 must be considered for the advance of the quality of the research on COVID0-19. First, the importance of employing all-cause mortality rate, not “COVID-19 related” mortality rate, as a hard outcome. The importance of this difference relies on the fact that mortality in COVID-19 is largely represented by indirect effects of COVID-19. In addition, whether deaths occurred due to COVID-19 or to a secondary complication is less critical than whether deaths occurred or not.

Second, the crisis of reproducibility in science, largely discussed in the scientific community, is particularly present in COVID-19, since mutations in the SARS-CoV-2 lead to changes in the efficacy of direct or indirect antiviral agents, as well as drugs that target the host cell. Changes in the efficacy of direct antivirals are easy to comprehend since these agents depend on specific portions of the virus to act. Conversely, mutations may also lead to changes in the level of dependency of SARS-CoV-2 on each of the steps for its cell entry, which may affect the ability of drugs that hamper one or more steps of the process of SARS-CoV-2 cell entry to prevent its successful entry. For instance, certain SARS-Cov-2 variants have a stronger dependency on the priming process that is enabled by TMPRSS2, while others tend to have their direct cell entry (not mediated by ACE2 facilitated) [36,41,42].

Due to the meaningful changes in the response to drugs according to the variant, we claim that studies on COVID-19 to test the efficacy of drugs should be considered as variant-dependent, i.e., the observed efficacy of a certain molecule for COVID-19 should be extrapolated for other variants with a cautious and lower degree of certainty, in a similar manner how vaccines for COVID-19 are analyzed.

Third, in COVID-19 studies, self-identified gender, rather than biological sex, should be considered to classify a participant of a research, in case the person is under hormonal therapy to adjust for the self-identified gender. The adjustment for the self-identified gender when under hormonal treatment is based on the fact that COVID-19 has androgen- and sex-specific risks, as observed in higher risk in males than in females [1], higher risk in women with hyperandrogenism than women without hyperandrogenic states [10,11], higher risk when on androgen therapy [7], lower risk when in anti-androgen therapy [12-17], lower in estrogen or progesterone therapy [22,44-46], and, in the case of transgenders, female-to-males (fTm), that are self-identified as males while biologically females, also termed as trans men, have higher risk than male-to-females (mTf), that are self-identified as females while biologically males, also termed as trans women [47]. This means that cis and transmen are clustered together, and in a different risk profile than cis and trans women, showing that self-identified gender, rather than biological sex, should be considered to classify within trials on COVID-19.

Forth, interference of politics in science and research on COVID-19 has negatively influenced the inherent impartiality required to evaluate and judge studies and manuscripts. In this matter, competing interests may exert indirect pressure for the acceptance or rejection of specific types of research, according to the results. Although this is speculative, inequalities in the level of severity of the review process in several scientific journals have become overwhelming. Due to the unclear interests that have increasingly influenced the scientific methodology and publication, we encourage all scientific community to be fully transparent by declaring all direct and indirect conflicts of interests, not only restricted to the current conflicts, but also extended to the previous conflicts and potential future conflicts, that could influence, at any extent, the results and analysis for both positive and negative responses, as well as decisions and evaluations when acting as reviewers or editors. We also claim that conflicts and competing interests to be more emphasized and visible within the manuscripts and publications.

The Proxa AndroCoV and Proxa-Rescue AndroCoV trials and the currently ongoing Phase 3 trials for COVID-19

In common, all RCTs with proxalutamide for COVID-19, including the RCT in outpatient settings with males, females, and in hospitalized patients for both males and females, were conducted as a sort of ‘add-on’ therapy, since the standard of care in both outpatients and hospitalized patients, and in both Northern and Southern Brazil in hospitalized patients, included drugs with efficacy demonstrated to be at least marginally effective, if not effective, including nitazoxanide, ivermectin, antibiotics, enoxaparin, glucocorticoids and other drug classes [48-51].

COVID-19 results from a complex pathophysiology caused by the SARS-CoV-2. Like other viruses, SARS-CoV-2 has multiple sites of action in order to cause the resulting disease, COVID-19. Consequently, once the pathophysiology is comprehended, it should not be expected that monotherapy would lead to important improvements in clinical outcomes, unless a drug has multiple actions or is used in a narrow window of opportunity, in the case of direct antivirals. And, in the last case, mutations in the virus may likely result in changes in the efficacy of direct antivirals.

Conversely, the combination of drugs that act in different sites may lead to a synergistic effect against the SARS-CoV-2, enhancing the efficacy, when compared to each drug alone. The same occurred to other viruses, including the ‘drug cocktail’ for the HIV [52,53], hepatitis B [54], and hepatitis C [55]. Indeed, the typical poor responses to viruses observed throughout history may be a consequence of the lack of optimized treatment regimens.

Whether proxalutamide exerted synergistic effects to the existing treatments drugs - i.e., proxalutamide was not only effective, but also enhanced the efficacy of the other drugs administered - or if proxalutamide worked alone is uncertain, since not only proxalutamide, but also no other antiandrogens have been tested against COVID-19 alone to date.

Unlike the AndroCoV trials, the Phase 3 trials currently ongoing (NCT05009732, NCT04870606 and NCT04869228) are testing proxalutamide in different populations without the rationale of the combination of different drugs as being a potential critical aspect for the efficacy of the treatment against COVID-19.

A second major difference between the AndroCoV Trials, specially the Proxa-Rescue AndroCoV Trial, is that proxalutamide may have been more effective for hospitalized COVID-19 patients due to the P.1 (gamma) variant, than what it would be for other variants. The key difference is that in the P.1 (Gamma) variant viral replication may persist during the second and third stages of the disease [36-39], demonstrated by a common yet unexpected increase in usCRP levels despite the use of high-dose glucocorticoid treatment regimens, not explained by bacterial or fungal infections, and also demonstrated by a persistent increase of D-dimer despite the use of potent anticoagulants, both of which indicating direct viral activity persisting throughout all COVID-19 stages [29,31].

Because of the marked differences in the pathophysiology, pathogenicity, and clinical course between SARS-CoV-2 variants, which is particularly overwhelming in the case of the P.1 (Gamma) variant, for which proxalutamide was tested in the Proxa-Rescue AndroCoV trial, whether proxalutamide will be as effective for COVID-19 caused other variants of the SARS-CoV-2 is unknown. In addition, the currently ongoing trials are not precise replications of the design of the AndroCoV Trials, in the essential aspect of the accompanying drug therapy. Due to the lack of similarity between the AndroCoV and the currently ongoing trials and the differences in the SARS-CoV-2 variants in which proxalutamide is being tested, distinct results in terms of drug efficacy are not unexpected.

Specific aspects of studies on anti-androgens for COVID-19

The role of androgens on COVID-19 is well established from multiple epidemiological observations [1-17,22], clinical studies [23-33], and strong and definitive biological plausibility [18-20, 40-43]. Even ADT for castration-resistant prostate cancer, which has been found to independently increase the risk of global mortality in case of any secondary illness, when compared to men not under ADT [56], demonstrated not only to have an exception when the disease is COVID-19, but also to provide relative protection from severe COVID-19 and death [14-17]. The harmful effects of androgens and protective roles of antiandrogens for COVID-19 have been questioned since low testosterone has been identified as a predictor of poor prognosis among males hospitalized due to COVID-19 [57]. However, the causality relationship between testosterone and disease severity may have been confounded, since in severe states, irrespective of the underlying disease, testosterone is dramatically reduced through multiple hypothalamic mechanisms, since the intense inflammatory response blunts gonadotropin releasing hormone (GnRH) pulses, until the need of an anti-anabolic, pro-catabolic state, prevailing the corticotropic over the gonadotropic axis, when under severe, acute conditions. As a result, low testosterone is more likely a marker of severity, rather than a causer [58].

Differences between first and second-generation NSAAs, or even between second-generation NSAAs are clinically demonstrated through the fact that failure of treatment of castration-resistant prostate cancer with an NSAA is overcome with another NSAA [25,59-61].

Proxalutamide has unique characteristics when compared to other second-generation NSAAs. It is likely the most potent, between five to ten times more potent than other NSAAs, has the ability to suppress the genetic expression of the AR gene, has important concurrent actions on the regulation of angiotensin-converting enzyme-2 (ACE2) expression, which is the gate and key regulator of SARS-CoV-2 entry in cells, and exerts important anti-inflammatory effects with dramatic blockage of tumor necrosis factor-alpha (TNF-alpha) and interleukin 1-beta (IL-1beta) [25,59,60].

Possibly, a beneficial proportion between the different levels of potency of proxalutamide when acting as an anti-androgen, ACE-2 regulator, and as an anti-inflammatory may justify its efficacy for COVID-19 throughout different stages of the disease. For instance, a strong anti-androgen without concurrent regulation of the inflammatory response and ACE-2 expression may cause more harm than good at specific stages of COVID-19. In addition, the minimum concentration to be effective as a regulator of the inflammatory response and ACE-2 modulator may be distinct from those used for castration-resistant prostate cancer. Finally, because of the specificities of each NSAA, we consider responses to a certain NSAA for COVID-19 to be considered as a partial drug-class effect. We encourage that conclusions regarding anti-androgens for COVID-19 should not be extrapolated for other NSAAs, in particular when results are negative, due to the numerous factors that may influence the efficacy of NSAA for COVID-19. Finally, researchers should avoid recommending against studies of an entire drug class in the case of anti-androgens, based on the lack of positive results of a single molecule, due to the multiple specificities and peculiarities of each anti-androgen, as described above.

Another critical aspect that must be emphasized when testing anti-androgens for COVID-19 is the treatment duration. In the case of proxalutamide, the risk of COVID-19 relapse and worsening with early interruption of proxalutamide was first identified when tested for early COVID-19 in outpatient settings [27-29]. Initially, the protocol was a three-day therapy of 200mg proxalutamide per day. However, two weeks after the beginning of the RCT on outpatients, the unblinded principal investigator noticed, exclusively in participants of the proxalutamide group, a remarkably faster recovery process in the first three days followed by sudden relapse and progression of the disease after discontinuing proxalutamide. This led us to increase the treatment duration from three to seven days, since its safety profile was well-established for chronic use, which was approved by the National Ethics Committee. After changing to seven days of therapy, relapses were no longer reported.

**Figure 9:**
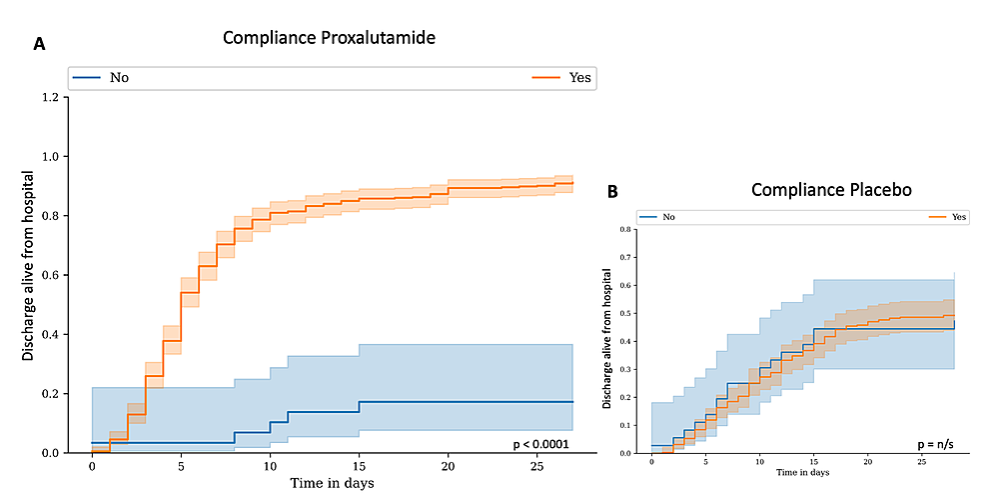
Proportion of patients discharged alive from the hospital according to compliance to treatment in proxalutamide (A) and in placebo (B) group.

Conversely, in the Proxa-Rescue AndroCoV trial, we detected a very high mortality rate (approximately 80% mortality rate) among participants that did not comply with the 14-day treatment, significantly higher than the placebo arm [32], while treatment completers had a mortality rate lower than 15%. Oppositely, compliance in the placebo group did not determine the survival rate, of approximately 50%, whether participants complied or not. Figure 9 demonstrates the differences between proxalutamide and placebo groups depending on the compliance to treatment.

The sudden loss of antiandrogen and anti-inflammatory protection may explain the dramatic relapse and progression of COVID-19. It is critical that the treatment duration of anti-androgens for COVID-19 should be strictly followed, and we highly recommend against short-duration treatments for COVID-19, of less than seven days for early, non-hospitalized COVID-19, and less than 14 days for hospitalized COVID-19.

Limitations

The Proxa-Rescue AndroCoV RCT was conducted in moderate-to-severe COVID-19 patients infected by the P.1 (Gamma) variant, when hospitals were collapsed in both Northern and Southern Brazil during the period of the study. Despite the demonstrated efficacy that was externally validated within the same study, it is unclear whether this can be reproduced under normal circumstances and in less pathogenic SARS-CoV-2 variants. The small number of subjects in the placebo group of the South arm may also have limited statistical analyses. For these reasons, we consider it indispensable that further RCTs in distinct environments should be conducted to confirm the present findings, with expected differences in terms of the level of efficacy.

## Conclusions

Proxalutamide avoided the progression of the disease in patients at later and more severe states of COVID-19, leading to a remarkable reduction in mortality rate, particularly in patients that required oxygen (scores 5 and 6 of the WHO COVID-19 Ordinary Score). The level of efficacy was similar between the North and South arms for different outcomes, which reinforces the consistency of the findings. Whether the level of efficacy of proxalutamide for hospitalized COVID-19 patients will remain in other SARS-CoV-2 variants is unknown. To our knowledge, this is the only study amongst studies that demonstrated an inter-region external validation within the same RCT.

## Appendices

**Table 9:** Study dataset.

Proxalutamide	North (0) or South (1)	Date of hospitalization	Randomization and treatment start	Interval (days) between hospitalization and randomization	Death (1) at Day 14	Death (1) at Day 28	Overall hospital length of stay	Post-randomization hospital length of stay	Age	Sex - Female (0), Male (1), Non-binary or intersex (2)	Day 0 (baseline)	Day 3	Day 7	Day 14	Day 28	Type 2 diabetes	Hypertension	Obesity	Pulmonary	Number of existing conditions	Enoxaparin 40mg SC twice daily throughout hospitalization	Rivaroxaban/Apixaban	Colchicine 0.5mg caps oral intake twice daily throughout hospitalization	Ascorbic acid (vitamin C) 500mg IV shot once daily throughout hospitalization	Zinc 20mg liquid once daily throughout hospitalization	Vitamin D 10,000 IU 01 cap. daily throughout hospitalization	Omeprazole 40mg IV once daily throughout hospitalization	Ceftriaxone 1g IV twice daily for 07 days	Azithromycin 500mg 1 tablet daily for 5 days	Clarithromycin 500mg IV once daily during hospitalization	Cefepime	Tazocin	Cefuroxime	Vancomycin	Meropenem	Methylprednisolone 62.5mg SC twice daily throughout hospitalization	Dexamethasone 6mg IV once daily during hospitalization	Prednisone	Bromhexine	Budesonide	Ivermectin	N-acetyl-cysteine	Hydroxychloroquine	Renal failure (creatinine increase > 100% compared to baseline)	Hepatitis (ALT > 150 U/L or > 100% increase compared to baseline)		Diarrhea	Abdominal pain	Vomiting	Headache	Depression	Irritability	Palpitations	Leg pain	Shortness of breath	Spontaneous erection
0	0	10-Jan-2021	1-Feb-2021	22	0	0	52	30	48	0	6	7	7	7	6	1	1	1	0	3	1	0	1	1	1	1	1	1	0	1	0	0	0	0	0	0	1	0	0	0	0	0	0	1	0	0	0	0	0	0	0	0	0	0	0	0
0	0	26-Jan-2021	2-Feb-2021	7	0	0	41	34	36	1	6	6	6	7	7	0	0	0	0	0	1	0	1	1	1	1	1	1	1	0	0	0	0	0	0	1	0	0	0	0	0	0	0	0	0	0	0	0	0	0	0	0	0	0	0	0
0	0	29-Jan-2021	2-Feb-2021	4	0	0	6	2	27	0	5	2	2	1	1	0	0	0	0	0	1	0	1	1	1	1	1	1	0	1	0	0	0	0	0	0	1	0	0	0	0	0	0	0	0	0	0	0	0	0	0	0	0	0	0	0
0	0	29-Jan-2021	2-Feb-2021	4	0	0	16	11	34	0	6	5	5	1	1	0	0	0	0	0	1	0	1	1	1	1	1	1	1	0	0	0	0	0	0	1	0	0	0	0	0	0	0	0	0	0	0	0	0	0	0	0	0	0	0	0
0	0	1-Feb-2021	2-Feb-2021	1	0	0	16	14	25	0	6	5	5	2	2	0	0	0	0	0	1	0	1	1	1	1	1	1	1	0	0	0	0	0	0	0	1	0	0	0	0	0	0	0	0	0	0	0	0	0	0	0	0	0	0	0
0	0	1-Feb-2021	2-Feb-2021	1	1	1	11	9	55	1	5	6	7	8	8	1	1	0	0	2	1	0	1	1	1	1	1	1	1	0	0	0	0	0	0	1	0	0	0	0	0	0	0	0	0	0	0	0	0	0	0	0	0	0	0	0
0	0	1-Feb-2021	2-Feb-2021	1	0	0	30	20	44	1	5	6	6	6	2	0	1	0	0	1	1	0	1	1	1	1	1	1	1	1	0	0	0	0	0	0	1	0	0	0	0	0	0	0	0	0	0	0	0	0	0	0	0	0	0	0
1	0	31-Jan-2021	2-Feb-2021	2	0	0	6	4	48	0	6	2	1	1	1	0	0	0	0	0	1	0	1	1	1	1	1	1	1	0	0	0	0	0	0	1	0	0	0	0	0	0	0	0	0	0	0	0	0	0	0	0	0	0	0	0
1	0	2-Feb-2021	2-Feb-2021	0	0	0	6	6	46	1	6	3	2	1	1	1	1	0	0	2	1	0	1	1	1	1	1	1	1	0	0	0	0	0	0	1	0	0	0	0	0	0	0	1	0	0	1	0	0	0	0	0	0	0	0	0
1	0	1-Feb-2021	2-Feb-2021	1	0	0	13	12	50	1	6	5	5	2	1	0	0	0	0	0	1	0	1	1	1	1	1	1	1	0	0	0	0	0	0	1	0	0	0	0	0	0	0	0	0	0	0	0	0	0	0	0	0	0	0	0
1	0	31-Jan-2021	2-Feb-2021	2	0	0	9	7	41	1	5	4	2	1	1	0	1	1	0	2	1	0	1	1	1	1	1	1	0	0	0	0	0	0	0	1	0	0	0	0	0	0	0	0	0	0	0	0	0	0	0	0	0	0	0	0
1	0	31-Jan-2021	2-Feb-2021	2	0	0	9	7	60	1	6	4	2	1	1	0	0	0	0	0	1	0	1	1	1	1	1	1	0	1	0	0	0	0	0	1	0	0	0	0	0	0	0	0	0	0	1	0	0	0	0	0	0	0	0	0
0	0	29-Jan-2021	3-Feb-2021	5	0	0	10	5	34	0	6	4	2	2	2	0	0	0	0	0	1	0	1	1	1	1	1	1	1	0	0	0	0	0	0	1	0	0	0	0	0	0	0	0	0	0	0	0	0	0	0	0	0	0	0	0
0	0	29-Jan-2021	3-Feb-2021	5	0	0	10	6	38	1	5	5	2	2	2	0	0	0	0	0	1	0	1	1	1	1	1	1	1	0	0	0	0	0	0	1	0	0	0	0	0	0	0	0	0	0	0	0	0	0	0	0	0	0	0	0
0	0	1-Feb-2021	3-Feb-2021	2	1	1	9	7	62	0	5	6	8	8	8	0	0	0	0	0	1	0	1	1	1	1	1	1	0	1	0	0	0	0	0	0	1	0	0	0	0	0	0	0	0	0	0	0	0	0	0	0	0	0	0	0
0	0	1-Feb-2021	3-Feb-2021	2	0	0	10	7	37	0	6	6	2	2	2	0	0	0	0	0	1	0	1	1	1	1	1	0	0	0	0	0	0	0	0	1	0	0	0	0	0	0	0	0	0	0	0	0	0	0	0	0	0	0	0	0
0	0	1-Feb-2021	3-Feb-2021	2	0	0	3	2	31	1	3	2	2	2	2	0	0	0	0	0	1	0	1	1	1	1	1	1	1	0	0	0	0	0	0	1	0	0	0	0	0	0	0	0	0	0	1	0	0	0	0	0	0	0	0	0
0	0	1-Feb-2021	3-Feb-2021	2	0	0	5	3	52	1	5	3	2	2	2	0	0	0	0	0	1	0	1	1	1	1	1	1	0	1	0	0	0	0	0	0	1	0	0	0	0	0	0	0	0	0	0	0	0	0	0	0	0	0	0	0
0	0	2-Feb-2021	3-Feb-2021	1	1	1	6	4	55	0	6	6	8	8	8	0	1	0	1	2	1	0	1	1	1	1	1	1	1	0	0	0	0	0	0	1	0	0	0	0	0	0	0	0	0	0	0	0	0	0	0	0	0	0	0	0
0	0	2-Feb-2021	3-Feb-2021	1	0	1	62	60	47	0	6	7	7	7	8	1	0	0	0	1	1	0	1	1	1	1	1	1	0	1	0	0	0	0	0	1	0	0	0	0	0	0	0	1	1	0	0	0	0	0	0	0	0	0	0	0
0	0	2-Feb-2021	3-Feb-2021	1	1	1	10	9	41	0	6	6	7	8	8	0	0	0	0	0	1	0	1	1	1	1	1	1	0	1	0	0	0	0	0	1	0	0	0	0	0	0	0	0	0	0	0	0	0	0	0	0	0	0	0	0
0	0	2-Feb-2021	3-Feb-2021	1	1	1	15	14	55	1	6	7	7	8	8	0	1	0	0	1	1	0	1	1	1	1	1	1	1	0	0	0	0	0	0	1	0	0	0	0	0	0	0	0	0	0	0	0	0	0	0	0	0	0	0	0
0	0	2-Feb-2021	3-Feb-2021	1	0	0	20	18	28	1	6	7	7	7	7	0	0	0	0	0	1	0	1	1	1	1	1	1	1	0	0	0	0	0	0	1	0	0	0	0	0	0	0	0	0	0	0	0	0	0	0	0	0	0	0	0
0	0	2-Feb-2021	3-Feb-2021	1	0	0	25	20	47	1	6	6	5	2	2	0	0	0	0	0	1	0	1	1	1	1	1	1	1	0	0	0	0	0	0	1	0	0	0	0	0	0	0	0	0	0	1	0	0	0	0	0	0	0	0	0
1	0	31-Jan-2021	3-Feb-2021	3	0	0	9	6	53	1	5	3	1	1	1	0	0	0	0	0	1	0	1	1	1	1	1	1	1	0	0	0	0	0	0	1	0	0	0	0	0	0	0	0	0	0	0	0	0	0	0	0	0	0	0	0
1	0	31-Jan-2021	3-Feb-2021	3	0	0	7	4	52	0	6	2	1	1	1	0	0	0	0	0	1	0	1	1	1	1	1	1	1	0	0	0	0	0	0	1	0	0	0	0	0	0	0	0	0	0	0	0	0	0	0	0	0	0	0	0
1	0	1-Feb-2021	3-Feb-2021	2	0	0	3	1	49	0	5	1	1	1	1	0	1	0	0	1	1	0	1	1	1	1	1	1	1	0	0	0	0	0	0	1	0	0	0	0	0	0	0	0	0	0	0	0	0	0	0	0	0	0	0	0
1	0	2-Feb-2021	3-Feb-2021	1	0	0	6	5	61	0	6	3	1	1	1	0	1	0	0	1	1	0	1	1	1	1	1	1	0	0	0	0	0	0	0	1	0	0	0	0	0	0	0	0	0	0	0	0	0	0	0	0	0	0	0	0
1	0	1-Feb-2021	3-Feb-2021	2	0	0	7	5	67	0	5	4	1	1	1	0	1	0	0	1	1	0	1	1	1	1	1	1	1	0	0	0	0	0	0	0	1	0	0	0	0	0	0	0	0	0	0	0	0	0	0	0	0	0	0	0
1	0	1-Feb-2021	3-Feb-2021	2	0	0	7	5	42	0	6	3	2	1	1	0	0	0	0	0	1	0	1	1	1	1	1	1	1	0	0	0	0	0	0	1	0	0	0	0	0	0	0	0	0	0	1	0	0	0	0	0	0	0	0	0
1	0	1-Feb-2021	3-Feb-2021	2	0	0	12	10	39	1	6	5	4	1	1	0	0	0	0	0	1	0	1	1	1	1	1	1	1	0	0	0	0	0	0	0	1	0	0	0	0	0	0	0	0	0	0	0	0	0	0	0	0	0	0	0
1	0	2-Feb-2021	3-Feb-2021	1	0	0	5	4	37	1	6	3	1	1	1	1	0	0	0	1	1	0	1	1	1	1	1	1	1	0	0	0	0	0	0	1	0	0	0	0	0	0	0	1	0	0	0	0	0	0	0	0	0	0	0	1
1	0	31-Jan-2021	3-Feb-2021	3	0	0	8	5	40	1	6	3	1	1	1	0	1	1	0	2	1	0	1	1	1	1	1	1	1	0	0	0	0	0	0	1	0	0	0	0	0	0	0	0	0	0	0	0	0	0	0	0	0	0	0	0
1	0	3-Feb-2021	3-Feb-2021	0	0	0	6	6	53	1	6	4	2	1	1	0	1	0	0	1	1	0	1	1	1	1	1	1	0	0	0	0	0	0	0	0	1	0	0	0	0	0	0	0	0	0	0	0	0	0	0	0	0	0	0	0
1	0	1-Feb-2021	3-Feb-2021	2	0	0	10	8	44	1	6	5	3	1	1	0	0	0	0	0	1	0	1	1	1	1	1	1	1	0	0	0	0	0	0	1	0	0	0	0	0	0	0	0	0	0	1	0	0	0	0	0	0	0	0	0
1	0	1-Feb-2021	3-Feb-2021	2	0	0	9	7	54	1	6	5	2	1	1	0	0	0	0	0	1	0	1	1	1	1	1	1	1	0	0	0	0	0	0	1	0	0	0	0	0	0	0	0	0	0	0	0	0	0	0	1	0	0	0	0
1	0	25-Jan-2021	3-Feb-2021	9	0	0	17	8	71	1	6	5	3	2	1	1	1	0	0	2	1	0	1	1	1	1	1	1	0	1	0	0	0	0	0	1	0	0	0	0	0	0	0	0	0	0	0	0	0	0	0	0	0	0	0	0
0	0	21-Jan-2021	4-Feb-2021	14	0	0	46	32	39	1	6	6	6	6	5	1	1	1	0	3	1	0	1	1	1	1	1	1	1	0	0	0	0	0	0	1	0	0	0	0	0	0	0	0	0	0	0	0	0	0	0	0	0	0	0	0
0	0	30-Jan-2021	4-Feb-2021	5	1	1	8	3	70	0	6	8	8	8	8	0	0	0	0	0	1	0	1	1	1	1	1	1	1	0	0	0	0	0	0	1	0	0	0	0	0	0	0	1	0	0	0	0	0	0	0	0	0	0	0	0
0	0	31-Jan-2021	4-Feb-2021	4	1	1	8	4	35	0	6	7	8	8	8	0	0	0	0	0	1	0	1	1	1	1	1	1	1	0	0	0	0	0	0	1	0	0	0	0	0	0	0	0	0	0	0	0	0	0	0	0	0	0	0	0
0	0	1-Feb-2021	4-Feb-2021	3	0	0	27	20	60	0	5	6	6	4	2	0	0	0	0	0	1	0	1	1	1	1	1	1	0	1	0	0	0	0	0	0	1	0	0	0	0	0	0	0	0	0	0	0	0	0	0	0	0	0	0	0
0	0	1-Feb-2021	4-Feb-2021	3	0	0	16	13	37	1	5	5	5	2	2	0	1	1	0	2	1	0	1	1	1	1	1	1	1	0	0	0	0	0	0	1	0	0	0	0	0	0	0	0	0	0	0	0	0	0	0	0	0	0	0	0
0	0	1-Feb-2021	4-Feb-2021	3	0	0	33	32	32	1	6	6	7	7	6	1	0	1	0	2	1	0	1	1	1	1	1	1	2	1	0	0	0	0	0	1	0	0	0	0	0	0	0	0	0	0	0	0	0	0	0	0	0	0	0	0
0	0	1-Feb-2021	4-Feb-2021	3	0	0	17	10	41	1	6	5	4	2	1	0	0	0	0	0	1	0	1	1	1	1	1	1	1	0	0	0	0	0	0	1	0	0	0	0	0	0	0	0	0	0	0	0	0	0	0	0	0	0	0	0
0	0	2-Feb-2021	4-Feb-2021	2	0	1	20	16	68	0	6	6	7	7	8	0	0	0	0	0	1	0	1	1	1	1	1	1	0	1	0	0	0	0	0	1	1	0	0	0	0	0	0	0	0	0	0	0	0	0	0	0	0	0	0	0
0	0	2-Feb-2021	4-Feb-2021	2	0	0	10	7	19	1	5	5	4	2	2	0	0	0	0	0	1	0	1	1	1	1	1	1	1	0	0	0	0	0	0	1	0	0	0	0	0	0	0	0	0	0	0	0	0	0	0	0	0	0	0	0
0	0	2-Feb-2021	4-Feb-2021	2	0	1	19	18	71	1	6	7	7	7	8	0	0	0	0	0	1	0	1	1	1	1	1	1	1	0	0	0	0	0	0	1	0	0	0	0	0	0	0	0	0	0	0	0	0	0	0	0	0	0	0	0
0	0	2-Feb-2021	4-Feb-2021	2	0	0	13	6	34	1	6	6	5	2	1	0	0	0	0	0	1	0	1	1	1	1	1	1	0	1	0	0	0	0	0	1	0	0	0	0	0	0	0	1	0	0	0	0	0	0	0	0	0	0	0	0
0	0	3-Feb-2021	4-Feb-2021	1	0	0	11	9	48	1	5	5	5	2	2	1	1	0	0	2	1	0	1	1	1	1	1	1	1	0	0	0	0	0	0	1	0	0	0	0	0	0	0	0	0	0	0	0	0	0	0	0	0	0	0	0
0	0	3-Feb-2021	4-Feb-2021	1	0	1	22	19	41	1	6	6	6	7	8	1	1	0	0	2	1	0	1	1	1	1	1	1	0	1	0	0	0	0	0	1	0	0	0	0	0	0	0	0	0	0	0	0	0	0	0	0	0	0	0	0
0	0	3-Feb-2021	4-Feb-2021	1	1	1	18	14	38	1	6	7	7	8	8	0	0	0	0	0	1	0	1	1	1	1	1	1	1	0	0	0	0	0	0	1	0	0	0	0	0	0	0	0	0	0	0	0	0	0	0	0	0	0	0	0
0	0	3-Feb-2021	4-Feb-2021	1	1	1	12	8	58	1	6	7	8	8	8	0	1	1	0	2	1	0	1	1	1	1	1	1	0	1	0	0	0	0	0	1	0	0	0	0	0	0	0	0	0	0	0	0	0	0	0	0	0	0	0	0
0	0	4-Feb-2021	4-Feb-2021	0	0	1	18	18	53	1	6	6	7	7	8	0	1	0	0	1	1	0	1	1	1	1	1	1	1	0	0	0	0	0	0	0	1	0	0	0	0	0	0	0	0	0	0	0	0	0	0	0	0	0	0	0
1	0	2-Feb-2021	4-Feb-2021	2	0	0	11	9	69	1	6	4	3	2	2	0	0	0	1	1	1	0	1	1	1	1	1	0	0	0	0	0	0	0	0	1	0	0	0	0	0	0	0	0	0	0	0	0	0	0	0	0	0	0	0	0
1	0	4-Feb-2021	4-Feb-2021	0	0	0	7	7	36	1	6	4	2	1	1	0	0	0	0	0	1	0	1	1	1	1	1	0	0	0	0	0	0	0	0	1	0	0	0	0	0	0	0	0	0	0	0	0	0	0	0	0	0	0	0	0
1	0	2-Feb-2021	4-Feb-2021	2	0	0	17	15	41	0	6	6	5	4	2	0	1	1	1	3	1	0	1	1	1	1	1	1	1	0	0	0	0	0	0	1	0	0	0	0	0	0	0	0	0	0	0	0	0	0	0	0	0	0	0	0
0	0	1-Feb-2021	5-Feb-2021	4	0	1	15	11	57	0	6	7	7	8	8	1	1	0	0	2	1	0	1	1	1	1	1	1	0	1	0	0	0	0	0	1	0	0	0	0	0	0	0	0	0	0	0	0	0	0	0	0	0	0	0	0
0	0	2-Feb-2021	5-Feb-2021	3	0	0	24	19	36	0	6	6	5	4	2	0	0	0	0	0	1	0	1	1	1	1	1	1	1	0	0	0	0	0	0	0	1	0	0	0	0	0	0	0	0	0	0	0	0	0	0	0	0	0	0	0
0	0	2-Feb-2021	5-Feb-2021	3	1	1	13	12	53	1	5	6	7	8	8	0	0	0	0	0	1	0	1	1	1	1	1	1	0	1	0	0	0	0	0	0	1	0	0	0	0	0	0	0	0	0	0	0	0	0	0	0	0	0	0	0
0	0	3-Feb-2021	5-Feb-2021	2	1	1	7	6	71	0	6	7	8	8	8	0	0	0	0	0	1	0	1	1	1	1	1	1	0	1	0	0	0	0	0	1	0	0	0	0	0	0	0	0	0	0	0	0	0	0	0	0	0	0	0	0
0	0	3-Feb-2021	5-Feb-2021	2	1	1	19	13	35	1	5	6	5	8	8	0	1	0	0	1	1	0	1	1	1	1	1	1	1	0	0	0	0	0	0	0	1	0	0	0	0	0	0	0	0	0	0	0	0	0	0	0	0	0	0	0
0	0	3-Feb-2021	5-Feb-2021	2	1	1	8	6	84	1	6	6	8	8	8	1	0	1	0	2	1	0	1	1	1	1	1	1	0	1	0	0	0	0	0	1	0	0	0	0	0	0	0	0	0	0	0	0	0	0	0	0	0	0	0	0
0	0	4-Feb-2021	5-Feb-2021	1	0	0	10	6	37	0	5	4	2	1	1	1	0	0	0	1	1	0	1	1	1	1	1	1	0	1	0	0	0	0	0	1	0	0	0	0	0	0	0	1	1	0	0	0	0	0	0	0	0	0	0	0
0	0	4-Feb-2021	5-Feb-2021	1	0	1	24	17	37	0	6	6	7	7	8	0	0	0	0	0	1	0	1	1	1	1	1	0	0	0	0	0	0	0	0	1	0	0	0	0	0	0	0	0	0	0	0	0	0	0	0	0	0	0	0	0
0	0	4-Feb-2021	5-Feb-2021	1	0	0	17	16	46	1	5	5	4	3	2	0	1	0	0	1	1	0	1	1	1	1	1	1	1	0	0	0	0	0	0	1	0	0	0	0	0	0	0	0	0	0	0	0	0	0	0	0	0	0	0	0
0	0	4-Feb-2021	5-Feb-2021	1	0	0	15	14	70	1	6	5	5	2	1	0	0	0	0	0	1	0	1	1	1	1	1	1	0	1	0	0	0	0	0	1	1	0	0	0	0	0	0	0	0	0	0	0	0	0	0	0	0	0	0	0
1	0	4-Feb-2021	5-Feb-2021	1	0	0	14	13	58	1	5	4	3	1	1	1	1	1	0	3	1	0	1	1	1	1	1	1	1	0	0	0	0	0	0	1	0	0	0	0	0	0	0	0	0	0	0	0	0	0	0	0	0	0	0	0
1	0	3-Feb-2021	5-Feb-2021	2	0	0	9	7	57	1	6	3	2	1	1	1	1	1	0	3	1	0	1	1	1	1	1	1	0	1	0	0	0	0	0	1	1	0	0	0	0	0	0	0	0	0	0	0	0	0	0	0	0	0	0	0
1	0	4-Feb-2021	5-Feb-2021	1	0	0	8	7	60	0	6	4	2	1	1	0	0	0	0	0	1	0	1	1	1	1	1	1	1	0	0	0	0	0	0	1	0	0	0	0	0	0	0	0	0	0	1	0	0	0	0	0	0	0	0	0
1	0	3-Feb-2021	5-Feb-2021	2	0	0	16	14	62	1	6	4	4	2	1	0	1	0	1	2	1	0	1	1	1	1	1	1	0	1	0	0	0	0	0	1	0	0	0	0	0	0	0	0	0	0	0	0	0	0	0	0	0	0	0	0
1	0	4-Feb-2021	5-Feb-2021	1	0	0	5	4	51	0	5	2	1	1	1	0	0	0	0	0	1	0	1	1	1	1	1	1	1	0	0	0	0	0	0	1	0	0	0	0	0	0	0	0	0	0	0	0	0	0	0	0	0	0	0	0
1	0	3-Feb-2021	5-Feb-2021	2	0	1	19	17	62	1	6	6	6	6	8	0	0	0	0	0	1	0	1	1	1	1	1	1	1	0	0	0	0	0	0	1	0	0	0	0	0	0	0	0	0	0	0	0	0	0	0	0	0	0	0	0
1	0	2-Feb-2021	5-Feb-2021	3	0	0	5	2	37	1	5	1	1	1	1	0	0	0	0	0	1	0	1	1	1	1	1	1	1	0	0	0	0	0	0	1	0	0	0	0	0	0	0	0	0	0	0	0	0	0	0	0	0	0	0	0
1	0	2-Feb-2021	5-Feb-2021	3	0	0	9	6	38	0	6	5	2	1	1	1	0	0	0	1	1	0	1	1	1	1	1	1	0	1	0	0	0	0	0	1	0	0	0	0	0	0	0	0	0	0	1	0	0	0	0	0	0	0	0	0
1	0	2-Feb-2021	5-Feb-2021	3	0	1	25	22	49	1	6	6	6	7	8	0	1	1	1	3	1	0	1	1	1	1	1	1	1	0	0	0	0	0	0	1	1	0	0	0	0	0	0	0	0	0	0	0	0	0	0	0	0	0	0	0
0	0	1-Feb-2021	6-Feb-2021	5	0	0	12	9	41	1	5	5	3	2	2	0	0	0	0	0	1	0	1	1	1	1	1	1	0	1	0	0	0	0	0	1	0	0	0	0	0	0	0	0	0	0	0	0	0	0	0	0	0	0	0	0
0	0	2-Feb-2021	6-Feb-2021	4	0	1	28	16	76	1	6	6	7	7	8	0	1	0	0	1	1	0	1	1	1	1	1	1	0	1	0	0	0	0	0	1	0	0	0	0	0	0	0	1	0	0	0	0	0	0	0	0	0	0	0	0
0	0	3-Feb-2021	6-Feb-2021	3	0	0	43	33	53	0	6	6	6	7	7	1	0	0	0	1	1	0	1	1	1	1	1	1	1	0	0	0	0	0	0	1	0	0	0	0	0	0	0	0	0	0	0	0	0	0	0	0	0	0	0	0
0	0	4-Feb-2021	6-Feb-2021	2	1	1	8	6	73	0	5	7	8	8	8	0	0	0	0	0	1	0	1	1	1	1	1	1	0	1	0	0	0	0	0	1	1	0	0	0	0	0	0	0	0	0	0	0	0	0	0	0	0	0	0	0
0	0	4-Feb-2021	6-Feb-2021	2	0	1	29	17	69	1	5	6	6	7	8	0	0	0	0	0	1	0	1	1	1	1	1	1	1	0	0	0	0	0	0	1	0	0	0	0	0	0	0	0	0	0	0	0	0	0	0	0	0	0	0	0
0	0	4-Feb-2021	6-Feb-2021	2	1	1	9	7	67	1	6	7	8	8	8	1	0	0	0	1	1	0	1	1	1	1	1	1	1	0	0	0	0	0	0	1	0	0	0	0	0	0	0	0	0	0	0	0	0	0	0	0	0	0	0	0
0	0	5-Feb-2021	6-Feb-2021	1	0	0	22	12	51	0	6	6	5	2	2	0	0	0	0	0	1	0	1	1	1	1	1	1	0	1	0	0	0	0	0	1	0	0	0	0	0	0	0	0	0	0	0	0	0	0	0	0	0	0	0	0
0	0	5-Feb-2021	6-Feb-2021	1	0	1	22	17	23	0	6	7	7	7	8	0	1	0	0	1	1	0	1	1	1	1	1	1	1	0	0	0	0	0	0	1	0	0	0	0	0	0	0	0	0	0	0	0	0	0	0	0	0	0	0	0
0	0	5-Feb-2021	6-Feb-2021	1	0	0	7	6	37	1	5	4	2	1	1	0	0	0	0	0	1	0	1	1	1	1	1	1	1	0	0	0	0	0	0	1	0	0	0	0	0	0	0	0	0	0	0	0	0	0	0	0	0	0	0	0
0	0	5-Feb-2021	6-Feb-2021	1	0	0	21	16	52	1	6	7	7	5	2	0	0	0	1	1	1	0	1	1	1	1	1	1	1	0	0	0	0	0	0	1	0	0	0	0	0	0	0	0	0	0	0	0	0	0	0	0	0	0	0	0
1	0	2-Feb-2021	6-Feb-2021	4	0	0	6	2	36	0	6	1	1	1	1	0	0	0	0	0	1	0	1	1	1	1	1	1	1	0	0	0	0	0	0	0	1	0	0	0	0	0	0	0	0	0	0	0	0	0	0	1	0	0	0	0
1	0	5-Feb-2021	6-Feb-2021	1	0	0	8	7	40	0	6	3	2	1	1	0	0	0	0	0	1	0	1	1	1	1	1	1	0	1	0	0	0	0	0	0	1	0	0	0	0	0	0	0	0	0	1	1	0	0	0	0	0	0	0	0
1	0	4-Feb-2021	6-Feb-2021	2	0	0	10	8	69	0	6	4	2	1	1	1	1	0	0	2	1	0	1	1	1	1	1	1	0	1	0	0	0	0	0	0	1	0	0	0	0	0	0	0	0	0	0	0	0	0	0	0	0	0	0	0
1	0	5-Feb-2021	6-Feb-2021	1	0	0	6	5	33	1	5	2	1	1	1	0	0	0	0	0	1	0	1	1	1	1	1	1	0	1	0	0	0	0	0	0	1	0	0	0	0	0	0	0	0	0	0	0	0	0	0	0	0	0	0	0
1	0	6-Feb-2021	6-Feb-2021	0	0	0	4	4	39	1	6	3	1	1	1	0	0	1	0	1	1	0	1	1	1	1	1	1	0	1	0	0	0	0	0	1	0	0	0	0	0	0	0	0	0	0	0	0	0	0	0	0	0	0	0	0
1	0	4-Feb-2021	6-Feb-2021	2	0	0	6	4	42	0	6	2	1	1	1	0	0	0	0	0	1	0	1	1	1	1	1	1	0	1	0	0	0	0	0	0	1	0	0	0	0	0	0	0	0	0	0	0	0	0	0	0	0	0	0	0
1	0	2-Feb-2021	6-Feb-2021	4	0	0	7	3	51	1	6	2	1	1	1	0	0	0	0	0	1	0	1	1	1	1	1	1	0	1	0	0	0	0	0	0	1	0	0	0	0	0	0	0	0	0	0	0	0	0	0	0	0	0	0	0
1	0	5-Feb-2021	6-Feb-2021	1	0	0	8	7	66	0	6	3	2	1	1	0	1	0	0	1	1	0	1	1	1	1	1	1	0	1	0	0	0	0	0	1	0	0	0	0	0	0	0	0	0	0	1	0	0	0	0	0	0	0	0	0
1	0	5-Feb-2021	6-Feb-2021	1	0	0	7	6	39	1	6	3	1	1	1	0	0	0	0	0	1	0	1	1	1	1	1	1	1	0	0	0	0	0	0	1	0	0	0	0	0	0	0	0	0	0	0	0	0	0	0	0	0	0	0	0
1	0	5-Feb-2021	6-Feb-2021	1	0	0	21	20	52	1	6	5	5	4	2	0	1	0	0	1	1	0	1	1	1	1	1	1	1	0	0	0	0	0	0	1	0	0	0	0	0	0	0	0	0	0	0	0	0	0	0	1	0	0	0	0
1	0	4-Feb-2021	6-Feb-2021	2	0	0	8	6	52	1	5	3	1	1	1	0	0	0	0	0	1	0	1	1	1	1	1	1	1	0	0	0	0	0	0	1	0	0	0	0	0	0	0	0	0	0	0	0	0	0	0	0	0	0	0	0
1	0	5-Feb-2021	6-Feb-2021	1	0	0	10	9	59	0	5	3	3	2	1	0	0	0	0	0	1	0	1	1	1	1	1	1	0	0	0	0	0	0	0	0	0	0	0	0	0	0	0	0	0	0	1	0	0	0	0	0	0	0	0	0
1	0	4-Feb-2021	6-Feb-2021	2	0	0	13	11	68	1	6	5	5	2	1	0	0	0	0	0	1	0	1	1	1	1	1	1	1	0	0	0	0	0	0	1	0	0	0	0	0	0	0	0	0	0	1	0	0	0	0	0	0	0	0	0
0	0	2-Feb-2021	7-Feb-2021	5	0	0	18	17	49	1	6	6	6	5	2	0	0	0	0	0	1	0	1	1	1	1	1	1	1	0	0	0	0	0	0	1	0	0	0	0	0	0	0	0	0	0	0	0	0	0	0	0	0	0	0	0
0	0	2-Feb-2021	7-Feb-2021	5	0	0	17	14	44	1	6	5	4	2	1	0	0	0	0	0	1	0	1	1	1	1	1	1	1	0	0	0	0	0	0	1	0	0	0	0	0	0	0	0	0	0	0	0	0	0	0	0	0	0	0	0
0	0	3-Feb-2021	7-Feb-2021	4	1	1	4	3	65	0	6	8	8	8	8	0	0	0	0	0	1	0	1	1	1	1	1	1	1	0	0	0	0	0	0	1	0	0	0	0	0	0	0	0	1	0	0	0	0	0	0	0	0	0	0	0
0	0	3-Feb-2021	7-Feb-2021	4	0	0	50	43	67	1	6	6	7	7	7	0	1	0	0	1	1	0	1	1	1	1	1	1	1	0	0	0	0	0	0	1	0	0	0	0	0	0	0	0	0	0	0	0	0	0	0	0	0	0	0	0
0	0	5-Feb-2021	7-Feb-2021	2	0	0	18	12	54	0	6	6	5	2	2	0	1	0	0	1	1	0	1	1	1	1	1	1	1	0	0	0	0	0	0	1	0	0	0	0	0	0	0	0	0	0	0	0	0	0	0	0	0	0	0	0
0	0	5-Feb-2021	7-Feb-2021	2	0	0	23	17	56	0	6	6	6	5	2	0	0	0	0	0	1	0	1	1	1	1	1	1	0	1	0	0	0	0	0	1	1	0	0	0	0	0	0	0	0	0	0	0	0	0	0	0	0	0	0	0
0	0	5-Feb-2021	7-Feb-2021	2	0	0	18	16	33	1	6	5	5	4	2	0	0	0	0	0	1	0	1	1	1	1	1	1	1	0	0	0	0	0	0	0	1	0	0	0	0	0	0	0	0	0	0	0	0	0	0	0	0	0	0	0
0	0	6-Feb-2021	7-Feb-2021	1	0	1	20	16	57	0	6	5	4	7	8	0	0	0	0	0	1	0	1	1	1	1	1	1	1	0	0	0	0	0	0	1	0	0	0	0	0	0	0	0	0	0	0	0	0	0	0	0	0	0	0	0
0	0	6-Feb-2021	7-Feb-2021	1	1	1	7	5	69	1	6	7	8	8	8	0	0	0	0	0	1	0	1	1	1	1	1	1	0	1	0	0	0	0	0	1	0	0	0	0	0	0	0	0	0	0	0	0	0	0	0	0	0	0	0	0
0	0	6-Feb-2021	7-Feb-2021	1	0	0	21	15	44	1	6	5	4	3	2	0	1	1	0	2	1	0	1	1	1	1	1	1	1	0	0	0	0	0	0	1	0	0	0	0	0	0	0	0	0	0	0	0	0	0	0	0	0	0	0	0
1	0	6-Feb-2021	7-Feb-2021	1	0	0	4	3	32	0	5	2	1	1	1	0	0	0	0	0	1	0	1	1	1	1	1	1	0	1	0	0	0	0	0	1	0	0	0	0	0	0	0	0	0	0	1	0	0	0	0	0	0	0	0	0
1	0	5-Feb-2021	7-Feb-2021	2	0	0	3	1	49	1	4	1	1	1	1	0	0	0	0	0	1	0	1	1	1	1	1	1	1	0	0	0	0	0	0	1	0	0	0	0	0	0	0	0	0	0	0	0	0	0	0	0	0	0	0	0
1	0	21-Jan-2021	7-Feb-2021	17	1	1	25	8	58	1	6	7	7	8	8	0	1	0	1	2	1	0	1	1	1	1	1	1	0	0	0	0	0	0	0	1	1	0	0	0	0	0	0	1	0	0	1	0	0	0	0	0	0	0	0	0
0	0	24-Jan-2021	8-Feb-2021	15	0	0	28	12	41	0	4	6	4	1	1	0	0	0	0	0	1	0	1	1	1	1	1	1	0	1	0	0	0	0	0	1	0	0	0	0	0	0	0	0	0	0	0	0	0	0	0	0	0	0	0	0
0	0	1-Feb-2021	8-Feb-2021	7	0	1	29	24	56	0	6	7	7	8	8	0	0	0	0	0	1	0	1	1	1	1	1	1	0	0	0	0	0	0	0	1	0	0	0	0	0	0	0	0	0	0	0	0	0	0	0	0	0	0	0	0
0	0	1-Feb-2021	8-Feb-2021	7	0	0	30	27	89	1	6	6	6	5	3	0	0	0	0	0	1	0	1	1	1	1	1	1	0	1	0	0	0	0	0	0	1	0	0	0	0	0	0	0	0	0	0	0	0	0	0	0	0	0	0	0
0	0	3-Feb-2021	8-Feb-2021	5	1	1	9	7	57	1	5	7	8	8	8	0	0	1	1	2	1	0	1	1	1	1	1	1	1	0	0	0	0	0	0	1	0	0	0	0	0	0	0	0	0	0	0	0	0	0	0	0	0	0	0	0
0	0	4-Feb-2021	8-Feb-2021	4	1	1	10	9	47	0	6	6	7	8	8	0	0	0	0	0	1	0	1	1	1	1	1	1	0	1	0	0	0	0	0	1	0	0	0	0	0	0	0	0	0	0	1	0	0	0	0	0	0	0	0	0
0	0	6-Feb-2021	8-Feb-2021	2	1	1	11	9	78	0	5	6	6	8	8	1	1	1	0	3	1	0	1	1	1	1	1	1	1	0	0	0	0	0	0	1	0	0	0	0	0	0	0	0	0	0	0	0	0	0	0	0	0	0	0	0
0	0	6-Feb-2021	8-Feb-2021	2	0	0	12	9	43	0	6	6	4	2	2	0	0	0	0	0	1	0	1	1	1	1	1	1	0	1	0	0	0	0	0	1	1	0	0	0	0	0	0	0	0	0	0	0	0	0	0	0	0	0	0	0
0	0	6-Feb-2021	8-Feb-2021	2	0	0	23	22	34	0	6	6	5	4	2	0	0	0	0	0	1	0	1	1	1	1	1	1	0	1	0	0	0	0	0	1	0	0	0	0	0	0	0	0	0	0	0	0	0	0	0	0	0	0	0	0
0	0	6-Feb-2021	8-Feb-2021	2	0	0	12	9	45	0	6	5	4	2	2	0	0	0	0	0	1	0	1	1	1	1	1	1	1	0	0	0	0	0	0	1	0	0	0	0	0	0	0	0	0	0	0	0	0	0	0	0	0	0	0	0
0	0	7-Feb-2021	8-Feb-2021	1	1	1	10	9	50	0	6	7	7	8	8	0	0	0	0	0	1	0	1	1	1	1	1	1	1	0	0	0	0	0	0	1	0	0	0	0	0	0	0	0	0	0	0	0	0	0	0	0	0	0	0	0
0	0	7-Feb-2021	8-Feb-2021	1	1	1	8	5	78	1	5	6	8	8	8	0	0	0	0	0	1	0	1	1	1	1	1	1	0	1	0	0	0	0	0	1	0	0	0	0	0	0	0	1	0	0	0	0	0	0	0	0	0	0	0	0
0	0	7-Feb-2021	8-Feb-2021	1	1	1	8	5	57	1	5	6	8	8	8	0	0	0	0	0	1	0	1	1	1	1	1	1	1	0	0	0	0	0	0	1	0	0	0	0	0	0	0	0	0	0	0	0	0	0	0	0	0	0	0	0
0	0	7-Feb-2021	8-Feb-2021	1	0	0	19	17	44	1	5	6	5	4	1	0	0	0	1	1	1	0	1	1	1	1	1	1	0	1	0	0	0	0	0	1	0	0	0	0	0	0	0	0	0	0	0	0	0	0	0	0	0	0	0	0
0	0	7-Feb-2021	8-Feb-2021	1	1	1	4	3	47	1	6	8	8	8	8	0	1	0	0	1	1	0	1	1	1	1	1	1	1	0	0	0	0	0	0	0	1	0	0	0	0	0	0	0	0	0	0	0	0	0	0	0	0	0	0	0
0	0	7-Feb-2021	8-Feb-2021	1	1	1	11	6	52	1	6	6	8	8	8	0	0	0	0	0	1	0	1	1	1	1	1	1	1	0	0	0	0	0	0	1	0	0	0	0	0	0	0	0	0	0	0	0	0	0	0	0	0	0	0	0
1	0	6-Feb-2021	8-Feb-2021	2	0	0	7	5	46	0	6	4	2	1	1	0	1	0	0	1	1	0	1	1	1	1	1	1	0	1	0	0	0	0	0	1	0	0	0	0	0	0	0	0	0	0	0	0	0	0	0	0	0	0	0	0
0	0	24-Jan-2021	9-Feb-2021	16	0	0	42	27	34	0	6	6	6	6	5	0	1	0	0	1	1	0	1	1	1	1	1	1	1	0	0	0	0	0	0	1	0	0	0	0	0	0	0	0	0	0	0	0	0	0	0	0	0	0	0	0
0	0	2-Feb-2021	9-Feb-2021	7	0	0	11	9	30	1	4	4	4	2	1	0	0	0	0	0	1	0	1	1	1	1	1	1	0	1	0	0	0	0	0	1	1	0	0	0	0	0	0	0	0	0	0	0	0	0	0	0	0	0	0	0
0	0	2-Feb-2021	9-Feb-2021	7	0	0	24	16	46	1	5	6	6	4	2	0	0	0	0	0	1	0	1	1	1	1	1	1	1	0	0	0	0	0	0	0	1	0	0	0	0	0	0	0	0	0	0	0	0	0	0	0	0	0	0	0
0	0	3-Feb-2021	9-Feb-2021	6	1	1	30	12	49	0	6	6	6	8	8	0	0	0	0	0	1	0	1	1	1	1	1	1	1	0	0	0	0	0	0	1	0	0	0	0	0	0	0	0	0	0	0	0	0	0	0	0	0	0	0	0
0	0	4-Feb-2021	9-Feb-2021	5	0	0	17	11	57	0	6	5	5	2	1	0	0	0	0	0	1	0	1	1	1	1	1	1	1	0	0	0	0	0	0	1	0	0	0	0	0	0	0	0	0	0	0	0	0	0	0	0	0	0	0	0
0	0	5-Feb-2021	9-Feb-2021	4	1	1	15	14	43	0	6	6	7	8	8	0	0	0	0	0	1	0	1	1	1	1	1	1	1	0	0	0	0	0	0	0	1	0	0	0	0	0	0	0	0	0	0	0	0	0	0	0	0	0	0	0
0	0	6-Feb-2021	9-Feb-2021	3	1	1	7	6	87	0	6	7	8	8	8	0	0	1	1	2	1	0	1	1	1	1	1	1	1	0	0	0	0	0	0	1	0	0	0	0	0	0	0	0	0	0	0	0	0	0	0	0	0	0	0	0
0	0	6-Feb-2021	9-Feb-2021	3	1	1	12	7	62	1	6	6	8	8	8	0	0	0	0	0	1	0	1	1	1	1	1	1	0	1	0	0	0	0	0	0	1	0	0	0	0	0	0	0	0	0	0	0	0	0	0	0	0	0	0	0
0	0	7-Feb-2021	9-Feb-2021	2	1	1	5	4	65	1	5	7	8	8	8	0	0	0	0	0	1	0	1	1	1	1	1	1	0	1	0	0	0	0	0	0	1	0	0	0	0	0	0	0	0	0	0	0	0	0	0	0	0	0	0	0
0	0	7-Feb-2021	9-Feb-2021	2	0	0	22	15	45	1	6	6	6	5	2	0	0	0	0	0	1	0	1	1	1	1	1	1	1	0	0	0	0	0	0	1	0	0	0	0	0	0	0	0	0	0	0	0	0	0	0	0	0	0	0	0
0	0	7-Feb-2021	9-Feb-2021	2	0	0	24	20	48	1	6	6	6	5	2	0	0	0	0	0	1	0	1	1	1	1	1	1	1	0	0	0	0	0	0	1	0	0	0	0	0	0	0	0	0	0	0	0	0	0	0	0	0	0	0	0
0	0	8-Feb-2021	9-Feb-2021	1	0	0	16	15	65	0	5	5	4	3	1	0	0	0	0	0	1	0	1	1	1	1	1	1	0	1	0	0	0	0	0	0	1	0	0	0	0	0	0	0	0	0	0	0	0	0	0	0	0	0	0	0
0	0	8-Feb-2021	9-Feb-2021	1	0	0	16	14	38	0	6	5	4	2	2	0	0	0	0	0	1	0	1	1	1	1	1	1	0	1	0	0	0	0	0	1	0	0	0	0	0	0	0	0	0	0	0	0	0	0	0	0	0	0	0	0
0	0	8-Feb-2021	9-Feb-2021	1	1	1	4	0	41	0	6	8	8	8	8	0	1	0	0	1	1	0	1	1	1	1	1	1	1	0	0	0	0	0	0	1	0	0	0	0	0	0	0	0	0	0	0	0	0	0	0	0	0	0	0	0
0	0	8-Feb-2021	9-Feb-2021	1	1	1	15	11	43	1	6	7	7	8	8	0	0	0	0	0	1	0	1	1	1	1	1	1	0	1	0	0	0	0	0	0	1	0	0	0	0	0	0	0	0	0	0	0	0	0	0	0	0	0	0	0
0	0	9-Feb-2021	9-Feb-2021	0	0	0	15	12	41	1	3	5	4	2	1	0	0	0	0	0	1	0	1	1	1	1	1	1	1	0	0	0	0	0	0	1	0	0	0	0	0	0	0	0	0	0	0	0	0	0	0	0	0	0	0	0
1	0	6-Feb-2021	9-Feb-2021	3	0	0	38	35	61	1	6	6	5	7	7	1	1	1	0	3	1	0	1	1	1	1	1	1	1	0	0	0	0	0	0	1	0	0	0	0	0	0	0	0	1	0	0	0	0	0	0	0	0	0	0	0
1	0	8-Feb-2021	9-Feb-2021	1	0	0	9	8	42	0	5	3	1	1	1	0	0	0	0	0	1	0	1	1	1	1	1	1	1	0	0	0	0	0	0	1	0	0	0	0	0	0	0	0	0	0	0	0	0	0	0	0	0	0	0	0
1	0	27-Jan-2021	9-Feb-2021	13	0	0	18	5	64	1	5	3	1	1	1	1	1	1	0	3	1	0	1	1	1	1	1	1	0	1	0	0	0	0	0	1	0	0	0	0	0	0	0	0	0	0	0	0	0	0	0	0	0	0	0	0
1	0	6-Feb-2021	9-Feb-2021	3	0	0	9	6	85	1	6	6	2	1	1	0	0	0	0	0	1	0	1	1	1	1	1	1	0	1	0	0	0	0	0	1	0	0	0	0	0	0	0	0	0	0	1	0	0	0	0	0	0	0	0	0
1	0	2-Feb-2021	9-Feb-2021	7	0	0	15	8	40	0	5	4	2	1	1	0	0	0	0	0	1	0	1	1	1	1	1	1	0	1	0	0	0	0	0	1	0	0	0	0	0	0	0	0	0	0	0	0	0	0	0	0	0	0	0	0
1	0	20-Jan-2021	9-Feb-2021	20	0	0	37	17	41	1	6	5	5	3	2	0	1	1	0	2	1	0	1	1	1	1	1	1	0	1	0	0	0	0	0	1	1	0	0	0	0	0	0	0	0	0	1	0	0	0	0	0	0	0	0	0
1	0	6-Feb-2021	9-Feb-2021	3	0	0	8	5	55	1	6	3	2	1	1	0	0	0	0	0	1	0	1	1	1	1	1	1	0	1	0	0	0	0	0	1	0	0	0	0	0	0	0	0	0	0	0	0	0	0	0	0	0	0	0	0
1	0	9-Feb-2021	9-Feb-2021	0	0	0	4	4	68	0	6	3	1	1	1	1	0	0	0	1	1	0	1	1	1	1	1	1	0	1	0	0	0	0	0	1	0	0	0	0	0	0	0	0	0	0	0	0	0	0	0	0	0	0	0	0
1	0	9-Feb-2021	9-Feb-2021	0	0	0	6	6	40	1	6	3	1	1	1	0	0	0	0	0	1	0	1	1	1	1	1	1	0	1	0	0	0	0	0	1	0	0	0	0	0	0	0	0	0	0	0	0	0	0	0	0	0	0	0	0
1	0	9-Feb-2021	9-Feb-2021	0	0	0	5	5	41	0	6	4	2	2	1	0	0	0	0	0	1	0	1	1	1	1	1	1	0	1	0	0	0	0	0	1	0	0	0	0	0	0	0	0	0	0	0	0	0	0	0	0	0	0	0	0
1	0	5-Feb-2021	9-Feb-2021	4	0	0	9	5	49	1	5	4	1	1	1	0	0	0	0	0	1	0	1	1	1	1	1	1	0	1	0	0	0	0	0	1	0	0	0	0	0	0	0	0	0	0	1	0	0	0	0	0	0	0	0	0
1	0	2-Feb-2021	9-Feb-2021	7	0	0	12	5	70	1	4	3	2	1	1	0	0	0	0	0	1	0	1	1	1	1	1	1	0	1	0	0	0	0	0	1	0	0	0	0	0	0	0	0	0	0	0	0	0	0	0	0	0	0	0	0
1	0	28-Jan-2021	9-Feb-2021	12	0	0	17	5	77	0	6	4	2	1	1	0	1	0	0	0	1	0	1	1	1	1	1	1	0	1	0	0	0	0	0	1	0	0	0	0	0	0	0	0	0	0	0	0	0	0	0	0	0	0	0	0
1	0	7-Feb-2021	9-Feb-2021	2	1	1	6	4	84	0	6	6	8	8	8	1	1	0	1	3	1	0	1	1	1	1	1	1	0	1	0	0	0	0	0	0	1	0	0	0	0	0	0	0	0	0	1	0	0	0	0	0	0	0	0	0
1	0	6-Feb-2021	9-Feb-2021	3	0	0	8	5	33	0	5	4	2	2	1	0	0	0	0	0	1	0	1	1	1	1	1	1	0	1	0	0	0	0	0	1	0	0	0	0	0	0	0	0	0	0	0	0	0	0	0	0	0	0	0	0
1	0	8-Feb-2021	9-Feb-2021	1	0	0	6	5	52	0	6	3	1	1	1	0	0	0	0	0	1	0	1	1	1	1	1	1	0	1	0	0	0	0	0	1	0	0	0	0	0	0	0	0	0	0	0	0	0	0	0	0	0	0	0	0
1	0	9-Feb-2021	9-Feb-2021	0	0	0	8	8	32	0	6	5	2	1	1	0	0	0	0	0	1	0	1	1	1	1	1	1	0	1	0	0	0	0	0	1	0	0	0	0	0	0	0	0	0	0	0	0	0	0	0	0	0	0	0	0
1	0	9-Feb-2021	9-Feb-2021	0	0	0	5	5	36	1	5	3	1	1	1	0	0	0	0	0	1	0	1	1	1	1	1	1	0	1	0	0	0	0	0	1	0	0	0	0	0	0	0	0	0	0	0	0	0	0	0	0	0	0	0	0
1	0	1-Feb-2021	9-Feb-2021	8	0	0	13	5	46	1	6	3	1	1	1	0	0	0	0	0	1	0	1	1	1	1	1	1	0	1	0	0	0	0	0	0	1	0	0	0	0	0	0	0	0	0	0	0	0	0	0	0	0	0	0	0
0	0	18-Jan-2021	10-Feb-2021	23	0	1	46	20	59	0	5	6	7	7	8	0	0	0	0	0	1	0	1	1	1	1	1	1	1	0	0	0	0	0	0	1	0	0	0	0	0	0	0	0	0	0	0	0	0	0	0	0	0	0	0	0
0	0	2-Feb-2021	10-Feb-2021	8	1	1	6	5	75	1	6	5	8	8	8	0	0	0	0	0	1	0	1	1	1	1	1	1	1	0	0	0	0	0	0	0	1	0	0	0	0	0	0	0	0	0	0	0	0	0	0	0	0	0	0	0
0	0	3-Feb-2021	10-Feb-2021	7	0	1	21	16	52	0	5	6	7	7	8	0	1	0	0	1	1	0	0	1	1	1	1	1	0	1	0	0	0	0	0	0	1	0	0	0	0	0	0	1	0	0	0	0	0	0	0	0	0	0	0	0
0	0	4-Feb-2021	10-Feb-2021	6	1	1	18	9	75	1	6	7	7	8	8	0	0	0	0	0	1	0	1	1	1	1	1	1	1	0	0	0	0	0	0	1	0	0	0	0	0	0	0	0	0	0	0	0	0	0	0	0	0	0	0	0
0	0	5-Feb-2021	10-Feb-2021	5	1	1	11	6	55	0	5	6	8	8	8	0	0	0	0	0	1	0	0	1	1	1	1	1	0	1	0	0	0	0	0	0	1	0	0	0	0	0	0	0	0	0	0	0	0	0	0	0	0	0	0	0
0	0	5-Feb-2021	10-Feb-2021	5	1	1	15	13	60	0	5	6	7	8	8	0	1	0	0	1	1	0	1	1	1	1	1	1	0	1	0	0	0	0	0	1	0	0	0	0	0	0	0	0	1	0	0	0	0	0	0	0	0	0	0	0
0	0	5-Feb-2021	10-Feb-2021	5	1	1	8	6	57	1	5	6	8	8	8	0	0	0	0	0	1	0	1	1	1	1	1	1	1	0	0	0	0	0	0	1	0	0	0	0	0	0	0	0	1	0	0	0	0	0	0	0	0	0	0	0
0	0	5-Feb-2021	10-Feb-2021	5	1	1	6	1	63	1	6	8	8	8	8	0	1	0	0	1	1	0	0	1	1	1	1	1	1	0	0	0	0	0	0	0	1	0	0	0	0	0	0	0	0	0	0	0	0	0	0	0	0	0	0	0
0	0	6-Feb-2021	10-Feb-2021	4	1	1	12	10	70	0	5	6	7	8	8	0	0	0	0	0	1	0	1	1	1	1	1	1	1	0	0	0	0	0	0	1	0	0	0	0	0	0	0	0	0	0	0	0	0	0	0	0	0	0	0	0
0	0	6-Feb-2021	10-Feb-2021	4	0	0	19	12	67	1	5	6	5	2	2	0	0	0	0	0	1	0	1	1	1	1	1	1	1	0	0	0	0	0	0	1	0	0	0	0	0	0	0	0	0	0	0	0	0	0	0	0	0	0	0	0
0	0	7-Feb-2021	10-Feb-2021	3	1	1	5	2	73	0	5	8	8	8	8	1	1	0	0	2	1	0	0	1	1	1	1	1	1	0	0	0	0	0	0	0	1	0	0	0	0	0	0	0	0	0	0	0	0	0	0	0	0	0	0	0
0	0	7-Feb-2021	10-Feb-2021	3	0	0	16	15	38	0	5	5	5	3	2	0	1	0	0	1	1	0	0	1	1	1	1	1	1	0	0	0	0	0	0	0	1	0	0	0	0	0	0	0	0	0	0	0	0	0	0	0	0	0	0	0
0	0	7-Feb-2021	10-Feb-2021	3	0	1	5	1	72	0	6	5	5	7	8	0	1	0	0	1	1	0	0	1	1	1	1	1	1	0	0	0	0	0	0	0	1	0	0	0	0	0	0	0	0	0	0	0	0	0	0	0	0	0	0	0
0	0	7-Feb-2021	10-Feb-2021	3	0	0	4	2	27	0	6	2	2	1	1	0	1	0	0	1	1	0	1	1	1	1	1	1	1	0	0	0	0	0	0	1	0	0	0	0	0	0	0	0	0	0	0	0	0	0	0	0	0	0	0	0
0	0	7-Feb-2021	10-Feb-2021	3	0	0	28	12	56	0	6	6	5	2	2	0	0	0	0	0	1	0	1	1	1	1	1	1	1	0	0	0	0	0	0	1	0	0	0	0	0	0	0	0	0	0	0	0	0	0	0	0	0	0	0	0
0	0	8-Feb-2021	10-Feb-2021	2	0	0	3	2	39	0	5	2	2	1	1	0	0	0	0	0	1	0	1	1	1	1	1	1	0	1	0	0	0	0	0	0	1	0	0	0	0	0	0	0	0	0	0	0	0	0	0	0	0	0	0	0
0	0	8-Feb-2021	10-Feb-2021	2	1	1	16	14	37	1	6	5	6	8	8	0	0	0	0	0	1	0	1	1	1	1	1	1	1	0	0	0	0	0	0	0	1	0	0	0	0	0	0	0	0	0	0	0	0	0	0	0	0	0	0	0
0	0	8-Feb-2021	10-Feb-2021	2	1	1	14	7	67	1	6	6	8	8	8	0	0	0	0	0	1	0	1	1	1	1	1	1	0	1	0	0	0	0	0	0	1	0	0	0	0	0	0	0	0	0	0	0	0	0	0	0	0	0	0	0
0	0	8-Feb-2021	10-Feb-2021	2	1	1	6	5	67	1	6	7	8	8	8	0	0	0	0	0	1	0	1	1	1	1	1	1	1	0	0	0	0	0	0	1	0	0	0	0	0	0	0	0	1	0	0	0	0	0	0	0	0	0	0	0
0	0	9-Feb-2021	10-Feb-2021	1	0	0	9	5	57	0	4	4	1	1	1	0	0	0	0	0	1	0	0	1	1	1	1	1	0	1	0	0	0	0	0	0	1	0	0	0	0	0	0	0	0	0	0	0	0	0	0	0	0	0	0	0
0	0	9-Feb-2021	10-Feb-2021	1	0	0	25	22	36	0	6	6	5	4	2	0	0	0	0	0	1	0	1	1	1	1	1	1	0	1	0	0	0	0	0	1	0	0	0	0	0	0	0	0	0	0	0	0	0	0	0	0	0	0	0	0
0	0	9-Feb-2021	10-Feb-2021	1	0	0	10	9	39	1	6	6	5	2	2	0	1	1	0	2	1	0	1	1	1	1	1	1	1	0	0	0	0	0	0	0	1	0	0	0	0	0	0	0	0	0	0	0	0	0	0	0	0	0	0	0
1	0	23-Jan-2021	10-Feb-2021	18	0	0	30	12	64	1	6	6	5	4	1	0	1	0	0	1	1	0	1	1	1	1	1	1	1	0	0	0	0	0	0	1	1	0	0	0	0	0	0	0	0	0	0	0	0	0	0	0	0	0	0	0
1	0	8-Feb-2021	10-Feb-2021	2	0	0	12	10	69	1	6	4	3	1	1	0	0	0	0	0	1	0	1	1	1	1	1	1	0	1	0	0	0	0	0	1	0	0	0	0	0	0	0	0	0	0	0	0	0	0	0	0	0	0	0	0
1	0	10-Feb-2021	10-Feb-2021	0	0	0	9	9	35	1	6	5	4	2	1	0	1	0	0	1	1	0	1	1	1	1	1	1	1	0	0	0	0	0	0	1	0	0	0	0	0	0	0	0	0	0	0	0	0	0	0	0	0	0	0	0
1	0	6-Feb-2021	10-Feb-2021	4	0	0	18	14	60	1	6	6	4	2	1	0	1	0	0	1	1	0	0	1	1	1	1	1	0	1	0	0	0	0	0	0	1	0	0	0	0	0	0	0	0	0	0	0	0	0	0	0	0	0	0	0
1	0	5-Feb-2021	10-Feb-2021	5	0	0	30	25	64	0	6	6	7	5	2	0	0	0	0	0	1	0	0	1	1	1	1	1	0	1	0	0	0	0	0	0	1	0	0	0	0	0	0	0	0	0	0	0	0	0	0	0	0	0	0	0
1	0	7-Feb-2021	10-Feb-2021	3	0	0	18	15	60	0	5	4	4	3	1	0	0	0	0	0	1	0	1	1	1	1	1	1	0	1	0	0	0	0	0	1	0	0	0	0	0	0	0	0	0	0	1	0	0	0	0	0	0	0	0	0
1	0	5-Feb-2021	10-Feb-2021	5	0	0	9	4	35	1	5	3	2	2	1	0	0	0	0	0	1	0	1	1	1	1	1	1	0	1	0	0	0	0	0	1	0	0	0	0	0	0	0	0	0	0	0	0	0	0	0	0	0	0	0	0
1	0	5-Feb-2021	10-Feb-2021	5	0	0	9	4	44	1	6	3	2	1	1	0	0	0	0	0	1	0	1	1	1	1	1	1	0	1	0	0	0	0	0	1	0	0	0	0	0	0	0	0	0	0	0	0	0	0	0	0	0	0	0	0
1	0	10-Feb-2021	10-Feb-2021	0	0	0	45	45	51	1	6	5	5	5	5	0	0	0	0	0	1	0	1	1	1	1	1	1	0	1	0	0	0	0	0	1	0	0	0	0	0	0	0	0	0	0	0	0	0	0	0	0	0	0	0	0
1	0	6-Feb-2021	10-Feb-2021	4	0	0	32	28	71	1	6	6	6	5	5	0	1	0	0	1	1	0	1	1	1	1	1	1	0	1	0	0	0	0	0	1	1	0	0	0	0	0	0	0	0	0	0	0	0	0	0	0	0	0	0	0
1	0	6-Feb-2021	10-Feb-2021	4	0	0	6	2	33	0	5	2	1	1	1	0	0	0	0	0	1	0	1	1	1	1	1	1	0	1	0	0	0	0	0	1	0	0	0	0	0	0	0	0	0	0	0	0	0	0	0	0	0	0	0	0
1	0	9-Feb-2021	10-Feb-2021	1	0	0	5	4	33	1	6	4	1	1	1	0	0	0	0	0	1	0	1	1	1	1	1	1	0	1	0	0	0	0	0	0	1	0	0	0	0	0	0	0	0	0	0	0	0	0	0	0	0	0	0	0
1	0	10-Feb-2021	10-Feb-2021	0	0	0	4	4	43	1	5	3	1	1	1	0	0	0	0	0	1	0	1	1	1	1	1	1	0	1	0	0	0	0	0	1	0	0	0	0	0	0	0	0	0	0	1	1	0	0	0	0	0	0	0	0
1	0	29-Jan-2021	10-Feb-2021	12	0	0	32	20	53	0	5	5	4	4	2	0	0	0	0	0	1	0	1	1	1	1	1	1	0	1	0	0	0	0	0	1	0	0	0	0	0	0	0	0	0	0	0	0	0	0	0	0	0	0	0	0
1	0	9-Feb-2021	10-Feb-2021	1	0	0	6	5	35	0	5	3	1	1	1	0	0	0	0	0	1	0	1	1	1	1	1	1	0	1	0	0	0	0	0	1	0	0	0	0	0	0	0	0	0	0	0	0	0	0	0	0	0	0	0	0
1	0	2-Feb-2021	10-Feb-2021	8	1	1	13	5	74	1	6	6	8	8	8	1	1	0	0	2	1	0	1	1	1	1	1	1	0	1	0	0	0	0	0	1	1	0	0	0	0	0	0	0	0	0	1	0	0	0	0	0	0	0	0	0
1	0	1-Feb-2021	10-Feb-2021	9	0	0	29	20	27	1	6	6	4	3	1	0	0	0	0	0	1	0	1	1	1	1	1	1	0	1	0	0	0	0	0	1	0	0	0	0	0	0	0	0	0	0	0	0	0	0	0	0	0	0	0	0
1	0	10-Feb-2021	10-Feb-2021	0	0	0	6	6	36	0	5	3	2	1	1	0	0	0	0	0	1	0	1	1	1	1	1	1	0	1	0	0	0	0	0	1	0	0	0	0	0	0	0	0	0	0	0	0	0	0	0	0	0	0	0	0
1	0	1-Feb-2021	10-Feb-2021	9	0	0	35	26	42	0	6	6	6	5	5	0	0	0	0	0	1	0	1	1	1	1	1	1	0	1	0	0	0	0	0	1	1	0	0	0	0	0	0	0	0	0	1	0	0	0	0	0	0	0	0	0
1	0	1-Feb-2021	10-Feb-2021	9	0	0	14	5	55	0	5	3	1	1	1	0	1	0	0	1	1	0	1	1	1	1	1	1	0	1	0	0	0	0	0	1	0	0	0	0	0	0	0	0	0	0	0	0	0	0	0	0	0	0	0	0
1	0	1-Feb-2021	10-Feb-2021	9	0	0	36	27	47	0	6	5	5	4	3	0	0	1	0	1	1	0	1	1	1	1	1	1	0	1	0	0	0	0	0	1	0	0	0	0	0	0	0	0	0	0	0	0	0	0	0	0	0	0	0	0
1	0	8-Feb-2021	10-Feb-2021	2	0	0	4	2	49	1	5	2	1	1	1	0	0	0	0	0	1	0	1	1	1	1	1	1	0	1	0	0	0	0	0	1	0	0	0	0	0	0	0	0	0	0	1	0	0	0	0	0	0	0	0	0
1	0	10-Feb-2021	10-Feb-2021	0	0	0	5	5	45	1	5	3	2	2	1	0	0	0	0	0	1	0	1	1	1	1	1	1	0	1	0	0	0	0	0	1	0	0	0	0	0	0	0	0	0	0	0	0	0	0	0	0	0	0	0	0
1	0	10-Feb-2021	10-Feb-2021	0	0	0	1	1	34	1	4	1	1	1	1	0	0	0	0	0	1	0	1	1	1	1	1	1	0	1	0	0	0	0	0	1	0	0	0	0	0	0	0	0	0	0	0	0	0	0	0	0	0	0	0	0
1	0	3-Feb-2021	10-Feb-2021	7	0	1	23	16	72	0	6	4	5	7	8	0	0	0	0	0	1	0	1	1	1	1	1	1	0	1	0	0	0	0	0	1	0	0	0	0	0	0	0	0	0	0	1	0	0	0	0	0	0	0	0	0
1	0	9-Feb-2021	10-Feb-2021	1	0	0	7	6	52	1	6	5	1	1	1	0	0	0	0	0	1	0	0	1	1	1	1	1	1	0	0	0	0	0	0	0	1	0	0	0	0	0	0	0	0	0	0	0	0	0	0	0	0	0	0	0
1	0	9-Feb-2021	10-Feb-2021	1	0	1	23	22	86	1	6	5	5	5	8	1	1	0	0	2	1	0	1	1	1	1	1	1	0	1	0	0	0	0	0	1	0	0	0	0	0	0	0	0	0	0	0	0	0	0	0	0	0	0	0	0
1	0	10-Feb-2021	10-Feb-2021	0	0	1	19	19	62	1	6	5	3	6	8	0	1	0	0	1	1	0	1	1	1	1	1	1	0	1	0	0	0	0	0	1	1	0	0	0	0	0	0	0	0	0	0	0	0	0	0	0	0	0	0	0
0	0	1-Feb-2021	11-Feb-2021	10	0	0	33	32	50	1	6	6	6	5	4	0	1	0	0	1	1	0	1	1	1	1	1	1	0	1	0	0	0	0	0	1	0	0	0	0	0	0	0	1	1	0	0	0	0	0	0	0	0	0	0	0
0	0	4-Feb-2021	11-Feb-2021	7	1	1	8	6	52	0	5	7	8	8	8	0	0	0	0	0	1	0	0	1	1	1	1	1	0	1	0	0	0	0	0	0	1	0	0	0	0	0	0	0	0	0	0	0	0	0	0	0	0	0	0	0
0	0	5-Feb-2021	11-Feb-2021	6	0	0	22	21	51	0	6	5	5	4	2	0	0	0	0	0	1	0	1	1	1	1	1	1	1	0	0	0	0	0	0	1	0	0	0	0	0	0	0	0	0	0	0	0	0	0	0	0	0	0	0	0
0	0	5-Feb-2021	11-Feb-2021	6	1	1	13	6	44	0	6	6	8	8	8	1	1	1	0	3	1	0	1	1	1	1	1	1	0	1	0	0	0	0	0	1	0	0	0	0	0	0	0	0	0	0	0	0	0	0	0	0	0	0	0	0
0	0	5-Feb-2021	11-Feb-2021	6	1	1	7	1	48	1	6	8	8	8	8	0	0	0	0	0	1	0	0	1	1	1	1	1	0	1	0	0	0	0	0	0	1	0	0	0	0	0	0	0	0	0	1	0	0	0	0	0	0	0	0	0
0	0	6-Feb-2021	11-Feb-2021	5	0	1	22	18	50	1	6	6	6	7	8	0	1	0	0	1	1	0	1	1	1	1	1	1	1	0	0	0	0	0	0	1	0	0	0	0	0	0	0	0	0	0	1	0	0	0	0	0	0	0	0	0
0	0	7-Feb-2021	11-Feb-2021	4	1	1	11	8	75	1	5	6	6	8	8	1	1	0	0	2	1	0	1	1	1	1	1	1	0	1	0	0	0	0	0	1	0	0	0	0	0	0	0	0	0	0	0	0	0	0	0	0	0	0	0	0
0	0	7-Feb-2021	11-Feb-2021	4	1	1	9	8	59	1	6	6	7	8	8	1	1	0	0	2	1	0	1	1	1	1	1	1	0	1	0	0	0	0	0	0	1	0	0	0	0	0	0	0	0	0	0	0	0	0	0	0	0	0	0	0
0	0	8-Feb-2021	11-Feb-2021	3	1	1	15	8	65	0	6	6	8	8	8	0	0	0	0	0	1	0	1	1	1	1	1	1	0	1	0	0	0	0	0	0	1	0	0	0	0	0	0	0	0	0	0	0	0	0	0	0	0	0	0	0
0	0	8-Feb-2021	11-Feb-2021	3	0	1	27	15	18	0	6	5	5	7	8	0	0	0	0	0	1	0	1	1	1	1	1	1	1	0	0	0	0	0	0	1	0	0	0	0	0	0	0	0	0	0	0	0	0	0	0	0	0	0	0	0
0	0	9-Feb-2021	11-Feb-2021	2	0	0	6	6	41	0	6	4	2	1	1	0	0	0	0	0	1	0	1	1	1	1	1	1	1	0	0	0	0	0	0	1	0	0	0	0	0	0	0	0	0	0	0	0	0	0	0	0	0	0	0	0
0	0	9-Feb-2021	11-Feb-2021	2	0	0	6	5	61	1	6	6	2	2	1	0	0	0	0	0	1	0	1	1	1	1	1	1	1	0	0	0	0	0	0	1	0	0	0	0	0	0	0	0	0	0	0	0	0	0	0	0	0	0	0	0
0	0	10-Feb-2021	11-Feb-2021	1	0	1	24	23	52	0	6	6	7	7	8	1	1	0	0	2	1	0	1	1	1	1	1	1	0	1	0	0	0	0	0	1	0	0	0	0	0	0	0	0	0	0	0	0	0	0	0	0	0	0	0	0
0	0	10-Feb-2021	11-Feb-2021	1	0	0	7	5	64	1	5	4	2	1	1	0	0	0	0	0	1	0	1	1	1	1	1	1	1	0	0	0	0	0	0	1	0	0	0	0	0	0	0	1	0	0	0	0	0	0	0	0	0	0	0	0
0	0	11-Feb-2021	11-Feb-2021	0	1	1	7	5	65	1	6	7	8	8	8	1	1	1	0	3	1	0	1	1	1	1	1	1	1	0	0	0	0	0	0	1	0	0	0	0	0	0	0	0	0	0	0	0	0	0	0	0	0	0	0	0
0	0	11-Feb-2021	11-Feb-2021	0	0	1	26	18	65	1	6	7	7	7	8	0	0	0	0	0	1	0	1	1	1	1	1	1	0	1	0	0	0	0	0	0	1	0	0	0	0	0	0	0	0	0	0	0	0	0	0	0	0	0	0	0
1	0	8-Feb-2021	11-Feb-2021	3	0	0	9	6	43	1	6	5	2	1	1	0	0	0	0	0	1	0	1	1	1	1	1	1	0	1	0	0	0	0	0	0	1	0	0	0	0	0	0	0	0	0	0	0	0	0	0	0	0	0	0	0
1	0	8-Feb-2021	11-Feb-2021	3	0	0	9	6	67	0	5	3	2	1	1	1	1	0	0	2	1	0	1	1	1	1	1	1	0	1	0	0	0	0	0	1	0	0	0	0	0	0	0	0	0	0	0	0	0	0	0	0	0	0	0	0
1	0	8-Feb-2021	11-Feb-2021	3	0	0	6	3	68	0	6	2	1	1	1	0	0	0	0	0	1	0	0	1	1	1	1	1	1	0	0	0	0	0	0	0	1	0	0	0	0	0	0	0	0	0	0	0	0	0	0	0	0	0	0	0
1	0	9-Feb-2021	11-Feb-2021	2	1	1	2	0	73	1	6	8	8	8	8	0	0	0	0	0	1	0	0	1	1	1	1	1	1	0	0	0	0	0	0	0	1	0	0	0	0	0	0	0	0	0	0	0	0	0	0	0	0	0	0	0
0	0	5-Feb-2021	12-Feb-2021	7	1	1	5	4	68	1	6	7	8	8	8	0	1	0	0	1	1	0	1	1	1	1	1	1	1	0	0	0	0	0	0	0	1	0	0	0	0	0	0	0	0	0	0	0	0	0	0	0	0	0	0	0
0	0	6-Feb-2021	12-Feb-2021	6	0	0	16	14	67	1	6	6	6	2	2	0	0	0	0	0	1	0	1	1	1	1	1	1	0	1	0	0	0	0	0	1	0	0	0	0	0	0	0	0	0	0	0	0	0	0	0	0	0	0	0	0
0	0	7-Feb-2021	12-Feb-2021	5	0	0	17	15	40	1	6	5	4	4	1	0	0	0	0	0	1	0	1	1	1	1	1	1	1	0	0	0	0	0	0	1	0	0	0	0	0	0	0	1	1	0	0	0	0	0	0	0	0	0	0	0
0	0	8-Feb-2021	12-Feb-2021	4	0	1	18	16	39	0	4	6	7	7	8	0	0	0	0	0	1	0	0	1	1	1	1	1	0	1	0	0	0	0	0	0	1	0	0	0	0	0	0	1	1	0	0	0	0	0	0	0	0	0	0	0
0	0	8-Feb-2021	12-Feb-2021	4	0	0	11	9	24	1	6	5	3	2	1	0	0	0	0	0	1	0	1	1	1	1	1	1	1	0	0	0	0	0	0	1	1	0	0	0	0	0	0	1	1	0	0	0	0	0	0	0	0	0	0	0
0	0	9-Feb-2021	12-Feb-2021	3	0	0	9	6	31	0	6	4	2	2	1	0	0	0	0	0	1	0	1	1	1	1	1	1	0	1	0	0	0	0	0	1	0	0	0	0	0	0	0	0	0	0	0	0	0	0	0	0	0	0	0	0
0	0	9-Feb-2021	12-Feb-2021	3	0	0	7	4	26	1	4	5	2	2	2	0	0	0	0	0	1	0	1	1	1	1	1	1	1	0	0	0	0	0	0	1	0	0	0	0	0	0	0	0	0	0	0	0	0	0	0	0	0	0	0	0
0	0	9-Feb-2021	12-Feb-2021	3	0	0	14	12	43	1	5	5	4	2	1	0	0	0	0	0	1	0	1	1	1	1	1	1	0	1	0	0	0	0	0	1	0	0	0	0	0	0	0	0	0	0	0	0	0	0	0	0	0	0	0	0
0	0	9-Feb-2021	12-Feb-2021	3	0	0	8	7	59	1	5	4	2	2	2	0	0	0	0	0	1	0	1	1	1	1	1	1	1	0	0	0	0	0	0	1	0	0	0	0	0	0	0	0	0	0	0	0	0	0	0	0	0	0	0	0
0	0	10-Feb-2021	12-Feb-2021	2	1	1	8	3	29	1	6	8	8	8	8	1	0	0	0	1	1	0	1	1	1	1	1	1	0	1	0	0	0	0	0	1	0	0	0	0	0	0	0	0	0	0	0	0	0	0	0	0	0	0	0	0
0	0	10-Feb-2021	12-Feb-2021	2	0	0	8	6	47	1	6	4	1	1	1	0	0	0	0	0	1	0	1	1	1	1	1	1	0	1	0	0	0	0	0	1	0	0	0	0	0	0	0	0	0	0	0	0	0	0	0	0	0	0	0	0
0	0	11-Feb-2021	12-Feb-2021	1	0	0	4	3	54	0	5	2	2	1	1	1	1	0	0	2	1	0	0	1	1	1	1	1	0	1	0	0	0	0	0	0	1	0	0	0	0	0	0	0	0	0	0	0	0	0	0	0	0	0	0	0
0	0	11-Feb-2021	12-Feb-2021	1	1	1	10	6	56	1	6	7	8	8	8	0	1	1	0	2	1	0	1	1	1	1	1	1	0	1	0	0	0	0	0	1	0	0	0	0	0	0	0	0	0	0	1	0	0	0	0	0	0	0	0	0
1	0	9-Feb-2021	12-Feb-2021	3	0	0	9	6	38	1	6	5	2	1	1	0	0	0	0	0	1	0	1	1	1	1	1	1	0	0	0	0	0	0	0	1	0	0	0	0	0	0	0	0	0	0	0	0	0	0	0	0	0	0	0	0
1	0	10-Feb-2021	12-Feb-2021	2	0	0	5	3	44	1	6	2	1	1	1	1	0	0	0	1	1	0	1	1	1	1	1	1	1	0	0	0	0	0	0	1	0	0	0	0	0	0	0	0	0	0	1	0	0	0	0	0	0	0	0	0
1	0	6-Feb-2021	12-Feb-2021	6	0	0	17	11	66	1	6	5	4	2	1	1	1	1	0	3	1	0	1	1	1	1	1	1	1	0	0	0	0	0	0	1	0	0	0	0	0	0	0	0	0	0	0	0	0	0	0	0	0	0	0	0
1	0	10-Feb-2021	12-Feb-2021	2	0	0	7	5	53	1	6	5	2	1	1	0	0	1	0	1	1	0	0	1	1	1	1	1	0	1	0	0	0	0	0	0	1	0	0	0	0	0	0	0	0	0	0	0	0	0	0	0	0	0	0	0
1	0	8-Feb-2021	12-Feb-2021	4	0	0	9	5	32	1	6	3	2	1	1	0	0	0	0	0	1	0	0	1	1	1	1	1	0	1	0	0	0	0	0	0	1	0	0	0	0	0	0	0	0	0	0	0	0	0	0	0	0	0	0	0
1	0	7-Feb-2021	12-Feb-2021	5	0	0	10	5	35	0	6	4	2	1	1	0	0	0	0	0	1	0	0	1	1	1	1	1	0	1	0	0	0	0	0	0	1	0	0	0	0	0	0	0	0	0	0	0	0	0	0	0	0	0	0	0
1	0	3-Feb-2021	12-Feb-2021	9	0	0	18	9	53	0	6	6	3	2	1	0	0	0	0	0	1	0	0	1	1	1	1	1	0	1	0	0	0	0	0	0	1	0	0	0	0	0	0	0	0	0	0	0	0	0	0	0	0	0	0	0
1	0	9-Feb-2021	12-Feb-2021	3	0	0	4	1	43	0	5	1	1	1	1	0	0	0	0	0	1	0	0	1	1	1	1	1	0	1	0	0	0	0	0	0	1	0	0	0	0	0	0	0	0	0	0	0	0	0	0	0	0	0	0	0
1	0	8-Feb-2021	12-Feb-2021	4	1	1	17	13	62	1	5	5	2	8	8	0	0	0	0	0	1	0	0	1	1	1	1	1	0	1	0	0	0	0	0	0	1	0	0	0	0	0	0	0	0	0	0	0	0	0	0	0	0	0	0	0
0	0	18-Jan-2021	13-Feb-2021	26	0	0	28	5	47	1	6	4	2	2	1	1	1	0	0	2	1	0	1	1	1	1	1	1	1	0	0	0	0	0	0	1	0	0	0	0	0	0	0	1	0	0	0	0	0	0	0	0	0	0	0	0
0	0	31-Jan-2021	13-Feb-2021	13	0	1	28	15	62	0	6	6	7	7	8	1	1	0	0	2	1	0	1	1	1	1	1	1	0	1	0	0	0	0	0	1	0	0	0	0	0	0	0	0	1	0	0	0	0	0	0	0	0	0	0	0
0	0	3-Feb-2021	13-Feb-2021	10	0	1	22	21	45	0	5	6	6	7	8	0	0	0	0	0	1	0	0	1	1	1	1	1	0	1	0	0	0	0	0	0	1	0	0	0	0	0	0	0	1	0	0	0	0	0	0	0	0	0	0	0
0	0	4-Feb-2021	13-Feb-2021	9	0	0	22	17	54	0	5	6	6	4	1	0	0	0	0	0	1	0	0	1	1	1	1	1	0	1	0	0	0	0	0	0	1	0	0	0	0	0	0	0	0	0	0	0	0	0	0	0	0	0	0	0
0	0	6-Feb-2021	13-Feb-2021	7	0	1	22	21	64	1	5	6	6	7	8	1	1	1	0	3	1	0	1	1	1	1	1	1	1	0	0	0	0	0	0	1	0	0	0	0	0	0	0	0	0	0	0	0	0	0	0	0	0	0	0	0
0	0	9-Feb-2021	13-Feb-2021	4	0	0	9	4	43	0	6	5	2	2	1	0	1	0	0	1	1	0	1	1	1	1	1	1	1	0	0	0	0	0	0	1	0	0	0	0	0	0	0	0	0	0	0	0	0	0	0	0	0	0	0	0
0	0	11-Feb-2021	13-Feb-2021	2	0	0	28	28	52	0	5	6	6	5	2	0	0	0	0	0	1	0	1	1	1	1	1	1	1	0	0	0	0	0	0	0	1	0	0	0	0	0	0	0	0	0	0	0	0	0	0	0	0	0	0	0
0	0	11-Feb-2021	13-Feb-2021	2	0	0	19	15	31	1	5	4	5	4	1	0	0	0	0	0	1	0	1	1	1	1	1	1	0	1	0	0	0	0	0	1	0	0	0	0	0	0	0	0	0	0	0	0	0	0	0	0	0	0	0	0
0	0	12-Feb-2021	13-Feb-2021	1	0	0	7	5	29	0	5	4	2	1	1	0	1	0	0	1	1	0	1	1	1	1	1	1	0	1	0	0	0	0	0	0	1	0	0	0	0	0	0	0	0	0	0	0	0	0	0	0	0	0	0	0
0	0	12-Feb-2021	13-Feb-2021	1	0	1	22	20	56	0	6	5	5	7	8	0	0	0	0	0	1	0	1	1	1	1	1	1	0	1	0	0	0	0	0	1	0	0	0	0	0	0	0	0	0	0	0	0	0	0	0	0	0	0	0	0
0	0	12-Feb-2021	13-Feb-2021	1	0	0	16	14	45	1	5	5	4	3	2	0	1	0	0	1	1	0	1	1	1	1	1	1	1	0	0	0	0	0	0	1	0	0	0	0	0	0	0	0	0	0	0	0	0	0	0	0	0	0	0	0
1	0	5-Feb-2021	13-Feb-2021	8	0	0	11	3	71	1	6	2	1	1	1	1	1	0	0	2	1	0	0	1	1	1	1	1	0	1	0	0	0	0	0	0	1	0	0	0	0	0	0	0	0	0	0	0	0	0	0	0	0	0	0	0
1	0	5-Feb-2021	13-Feb-2021	8	0	0	15	7	66	0	6	5	2	1	1	0	1	0	0	1	1	0	0	1	1	1	1	1	0	1	0	0	0	0	0	0	1	0	0	0	0	0	0	0	0	0	0	0	0	0	0	0	0	0	0	0
1	0	10-Feb-2021	13-Feb-2021	3	0	0	6	3	36	1	5	1	1	1	1	0	0	0	0	0	1	0	0	1	1	1	1	1	0	1	0	0	0	0	0	0	1	0	0	0	0	0	0	0	0	0	0	0	0	0	0	0	0	0	0	0
1	0	8-Feb-2021	13-Feb-2021	5	0	0	9	4	41	1	5	3	2	1	1	0	0	0	0	0	1	0	0	1	1	1	1	1	0	1	0	0	0	0	0	0	1	0	0	0	0	0	0	0	0	0	0	0	0	0	0	0	0	0	0	0
1	0	9-Feb-2021	13-Feb-2021	4	0	0	34	30	48	1	6	6	5	4	4	0	0	0	0	0	1	0	0	1	1	1	1	1	0	1	0	0	0	0	0	0	1	0	0	0	0	0	0	0	0	0	0	0	0	0	0	0	0	0	0	0
1	0	1-Feb-2021	13-Feb-2021	12	0	0	15	3	59	1	5	2	1	1	1	0	1	0	0	1	1	0	0	1	1	1	1	1	0	1	0	0	0	0	0	0	1	0	0	0	0	0	0	0	0	0	0	0	0	0	0	0	0	0	0	0
1	0	12-Feb-2021	13-Feb-2021	1	0	0	4	3	36	1	6	2	2	2	1	0	0	0	0	0	1	0	0	1	1	1	1	1	0	1	0	0	0	0	0	0	1	0	0	0	0	0	0	0	0	0	0	0	0	0	0	0	0	0	0	0
1	0	11-Feb-2021	13-Feb-2021	2	0	0	8	6	36	0	6	5	2	1	1	0	0	0	0	0	1	0	0	1	1	1	1	1	0	1	0	0	0	0	0	0	1	0	0	0	0	0	0	0	0	0	0	0	0	0	0	0	0	0	0	0
1	0	7-Feb-2021	13-Feb-2021	6	0	0	13	7	60	1	6	6	2	1	1	1	1	0	0	2	1	0	0	1	1	1	1	1	0	1	0	0	0	0	0	0	1	0	0	0	0	0	0	0	0	0	0	0	0	0	0	0	0	0	0	0
1	0	11-Feb-2021	13-Feb-2021	2	0	0	7	5	45	1	6	4	2	1	1	0	0	0	0	0	1	0	0	1	1	1	1	1	0	1	0	0	0	0	0	0	1	0	0	0	0	0	0	0	0	0	0	0	0	0	0	0	0	0	0	0
1	0	24-Jan-2021	13-Feb-2021	20	0	0	24	4	53	0	6	4	2	2	1	0	0	0	0	0	1	0	0	1	1	1	1	1	0	1	0	0	0	0	0	0	1	0	0	0	0	0	0	0	0	0	0	0	0	0	0	0	0	0	0	0
1	0	11-Feb-2021	13-Feb-2021	2	0	0	9	7	67	0	6	5	2	1	1	1	1	0	0	2	1	0	0	1	1	1	1	1	0	1	0	0	0	0	0	0	1	0	0	0	0	0	0	0	0	0	0	0	0	0	0	0	0	0	0	0
1	0	10-Feb-2021	13-Feb-2021	3	0	0	6	3	55	1	6	2	1	1	1	0	0	0	0	0	1	0	0	1	1	1	1	1	0	1	0	0	0	0	0	0	1	0	0	0	0	0	0	0	0	0	0	0	0	0	0	0	0	0	0	0
1	0	7-Feb-2021	13-Feb-2021	6	0	0	35	29	63	0	6	6	6	4	4	1	1	0	0	2	1	0	1	1	1	1	1	1	0	1	0	0	0	0	0	1	0	0	0	0	0	0	0	0	0	0	0	0	0	0	0	0	0	0	0	0
1	0	11-Feb-2021	13-Feb-2021	2	0	0	4	2	44	0	5	2	1	1	1	0	0	0	0	0	1	0	1	1	1	1	1	1	0	1	0	0	0	0	0	1	0	0	0	0	0	0	0	0	0	0	1	0	0	0	0	0	0	0	0	0
1	0	11-Feb-2021	13-Feb-2021	2	0	0	4	2	43	1	5	2	1	1	1	0	0	0	0	0	1	0	1	1	1	1	1	1	0	1	0	0	0	0	0	1	0	0	0	0	0	0	0	0	0	0	0	0	0	0	0	0	0	0	0	0
1	0	8-Feb-2021	13-Feb-2021	5	0	0	7	2	25	0	5	2	2	2	1	0	0	0	0	0	1	0	1	1	1	1	1	1	0	1	0	0	0	0	0	0	1	0	0	0	0	0	0	0	0	0	1	0	0	0	0	0	0	0	0	0
1	0	12-Feb-2021	13-Feb-2021	1	0	0	5	4	49	0	5	4	2	1	1	0	0	0	0	0	1	0	1	1	1	1	1	1	0	1	0	0	0	0	0	1	0	0	0	0	0	0	0	0	0	0	0	0	0	0	0	0	0	0	0	0
1	0	8-Feb-2021	13-Feb-2021	5	0	0	24	19	63	1	6	6	6	5	2	0	1	0	0	1	1	0	1	1	1	1	1	1	0	1	0	0	0	0	0	1	1	0	0	0	0	0	0	0	0	0	0	0	0	0	0	0	0	0	0	0
1	0	10-Feb-2021	13-Feb-2021	3	0	0	7	4	43	1	5	3	2	1	1	0	0	0	0	0	1	0	1	1	1	1	1	1	0	1	0	0	0	0	0	1	0	0	0	0	0	0	0	0	0	0	0	0	0	0	0	0	0	0	0	0
1	0	13-Feb-2021	13-Feb-2021	0	0	0	4	4	41	0	6	3	1	1	1	0	0	0	0	0	1	0	1	1	1	1	1	1	0	1	0	0	0	0	0	1	0	0	0	0	0	0	0	0	0	0	0	0	0	0	0	0	0	0	0	0
1	0	5-Feb-2021	13-Feb-2021	8	0	0	13	5	57	0	5	3	2	1	1	0	0	0	0	0	1	0	1	1	1	1	1	1	1	0	0	0	0	0	0	1	0	0	0	0	0	0	0	0	0	0	0	0	0	0	0	0	0	0	0	0
1	0	12-Feb-2021	13-Feb-2021	1	0	0	4	3	52	0	4	1	1	1	1	0	0	0	0	0	1	0	1	1	1	1	1	1	1	0	0	0	0	0	0	1	0	0	0	0	0	0	0	0	0	0	0	0	0	0	0	0	0	0	0	0
1	0	13-Feb-2021	13-Feb-2021	0	1	1	10	10	55	0	6	7	7	8	8	0	0	0	0	0	1	0	1	1	1	1	1	0	0	0	0	0	0	0	0	0	1	0	0	0	0	0	0	1	0	0	1	0	0	0	0	0	0	0	0	0
1	0	8-Feb-2021	13-Feb-2021	5	1	1	15	10	37	1	6	4	4	8	8	0	0	0	0	0	1	0	0	1	1	1	1	1	0	1	0	0	0	0	0	1	1	0	0	0	0	0	0	0	0	0	0	0	0	0	0	0	0	0	0	0
0	0	27-Jan-2021	14-Feb-2021	18	0	0	30	12	47	0	5	6	6	2	2	0	1	1	0	2	1	0	1	1	1	1	1	1	1	0	0	0	0	0	0	1	0	0	0	0	0	0	0	0	0	0	0	0	0	0	0	0	0	0	0	0
0	0	2-Feb-2021	14-Feb-2021	12	0	0	24	17	34	0	6	6	5	4	2	0	1	0	0	1	1	0	1	1	1	1	1	1	0	1	0	0	0	0	0	1	0	0	0	0	0	0	0	1	0	0	0	0	0	0	0	0	0	0	0	0
0	0	7-Feb-2021	14-Feb-2021	7	1	1	5	2	74	1	6	8	8	8	8	0	0	0	0	0	1	0	1	1	1	1	1	1	1	0	0	0	0	0	0	1	0	0	0	0	0	0	0	0	0	0	0	0	0	0	0	0	0	0	0	0
0	0	9-Feb-2021	14-Feb-2021	5	0	0	12	10	48	0	5	4	4	2	1	0	0	0	0	0	1	0	1	1	1	1	1	1	1	0	0	0	0	0	0	0	1	0	0	0	0	0	0	0	0	0	0	0	0	0	0	0	0	0	0	0
0	0	12-Feb-2021	14-Feb-2021	2	0	0	9	6	49	0	5	4	2	2	1	0	0	0	0	0	1	0	0	1	1	1	1	1	0	1	0	0	0	0	0	0	1	0	0	0	0	0	0	0	0	0	0	0	0	0	0	0	0	0	0	0
0	0	12-Feb-2021	14-Feb-2021	2	0	1	17	16	56	0	6	6	7	7	8	0	1	0	0	1	1	0	1	1	1	1	1	1	0	1	0	0	0	0	0	1	0	0	0	0	0	0	0	0	0	0	0	0	0	0	0	0	0	0	0	0
0	0	12-Feb-2021	14-Feb-2021	2	1	1	21	13	63	0	6	6	7	8	8	0	0	0	0	0	1	0	1	1	1	1	1	1	0	1	0	0	0	0	0	1	0	0	0	0	0	0	0	0	0	0	0	0	0	0	0	0	0	0	0	0
0	0	12-Feb-2021	14-Feb-2021	2	0	0	5	0	19	1	4	1	1	1	1	0	0	0	0	0	1	0	1	1	1	1	1	1	0	1	0	0	0	0	0	1	0	0	0	0	0	0	0	0	0	0	0	0	0	0	0	0	0	0	0	0
0	0	12-Feb-2021	14-Feb-2021	2	0	0	15	13	46	1	5	4	4	2	1	0	0	0	0	0	1	0	1	1	1	1	1	1	1	0	0	0	0	0	0	1	0	0	0	0	0	0	0	0	0	0	0	0	0	0	0	0	0	0	0	0
0	0	12-Feb-2021	14-Feb-2021	2	1	1	19	8	61	1	6	6	6	8	8	0	0	0	0	0	1	0	1	1	1	1	1	1	1	0	0	0	0	0	0	1	0	0	0	0	0	0	0	0	0	0	0	0	0	0	0	0	0	0	0	0
0	0	13-Feb-2021	14-Feb-2021	1	0	0	10	7	35	0	6	5	2	2	2	0	0	0	0	0	1	0	1	1	1	1	1	1	0	1	0	0	0	0	0	1	0	0	0	0	0	0	0	0	0	0	0	0	0	0	0	0	0	0	0	0
0	0	13-Feb-2021	14-Feb-2021	1	1	1	12	9	57	1	6	6	7	8	8	0	0	0	0	0	1	0	1	1	1	1	1	1	0	1	0	0	0	0	0	1	0	0	0	0	0	0	0	0	0	0	0	0	0	0	0	0	0	0	0	0
0	0	13-Feb-2021	14-Feb-2021	1	0	0	13	12	43	1	6	5	4	2	2	0	0	0	0	0	1	0	1	1	1	1	1	1	1	0	0	0	0	0	0	1	0	0	0	0	0	0	0	0	0	0	0	0	0	0	0	0	0	0	0	0
1	0	6-Feb-2021	14-Feb-2021	8	0	0	13	5	41	1	6	4	1	1	1	0	0	0	0	0	1	0	1	1	1	1	1	1	1	0	0	0	0	0	0	1	0	0	0	0	0	0	0	0	0	0	0	0	0	0	0	0	0	0	0	0
1	0	11-Feb-2021	14-Feb-2021	3	0	0	8	5	52	1	5	4	2	1	1	0	0	0	0	0	1	0	1	1	1	1	1	1	1	0	0	0	0	0	0	1	0	0	0	0	0	0	0	0	0	0	0	0	0	0	0	0	0	0	0	0
1	0	11-Feb-2021	14-Feb-2021	3	0	0	6	3	35	0	6	1	1	1	1	0	0	0	0	0	1	0	0	1	1	1	1	1	0	1	0	0	0	0	0	0	1	0	0	0	0	0	0	0	0	0	0	0	0	0	0	0	0	0	0	0
1	0	13-Feb-2021	14-Feb-2021	1	0	0	4	3	44	0	5	2	1	1	1	0	0	1	0	1	1	0	0	1	1	1	1	1	0	1	0	0	0	0	0	0	1	0	0	0	0	0	0	0	0	0	0	0	0	0	0	0	0	0	0	0
1	0	10-Feb-2021	14-Feb-2021	4	0	0	6	2	45	1	6	2	1	1	1	0	0	0	0	0	1	0	0	1	1	1	1	1	0	1	0	0	0	0	0	1	0	0	0	0	0	0	0	0	0	0	0	0	0	0	0	0	0	0	0	0
1	0	11-Feb-2021	14-Feb-2021	3	0	0	8	5	67	0	6	5	2	1	1	0	1	0	0	1	1	0	0	1	1	1	1	1	0	1	0	0	0	0	0	1	0	0	0	0	0	0	0	0	0	0	0	0	0	0	0	0	0	0	0	0
1	0	12-Feb-2021	14-Feb-2021	2	0	0	4	2	71	0	6	2	2	2	1	0	0	0	0	0	1	0	0	1	1	1	1	1	0	1	0	0	0	0	0	1	0	0	0	0	0	0	0	0	0	0	0	0	0	0	0	0	0	0	0	0
1	0	10-Feb-2021	14-Feb-2021	4	1	1	13	9	74	1	6	6	6	8	8	0	0	0	0	0	1	0	0	1	1	1	1	1	1	0	0	0	0	0	0	0	1	0	0	0	0	0	0	0	0	0	0	0	0	0	0	0	0	0	0	0
1	0	14-Feb-2021	14-Feb-2021	0	1	1	8	8	68	0	6	5	5	8	8	0	0	0	0	0	1	0	0	1	1	1	1	1	0	1	0	0	0	0	0	1	0	0	0	0	0	0	0	0	0	0	0	0	0	0	0	0	0	0	0	0
0	0	26-Jan-2021	15-Feb-2021	20	0	0	32	12	81	0	6	6	6	2	2	0	0	0	0	0	1	0	0	1	1	1	1	1	0	1	0	0	0	0	0	0	1	0	0	0	0	0	0	0	0	0	0	0	0	0	0	0	0	0	0	0
0	0	5-Feb-2021	15-Feb-2021	10	0	0	10	6	30	0	5	4	2	2	1	0	0	0	0	0	1	0	0	1	1	1	1	1	0	1	0	0	0	0	0	0	1	0	0	0	0	0	0	0	0	0	0	0	0	0	0	0	0	0	0	0
0	0	8-Feb-2021	15-Feb-2021	7	1	1	15	12	28	0	5	6	7	8	8	1	1	0	0	2	1	0	0	1	1	1	1	1	0	1	0	0	0	0	0	0	1	0	0	0	0	0	0	0	0	0	0	0	0	0	0	0	0	0	0	0
0	0	8-Feb-2021	15-Feb-2021	7	0	0	18	17	58	0	6	6	6	4	2	1	0	0	0	1	1	0	1	1	1	1	1	1	1	1	0	0	0	0	0	0	1	0	0	0	0	0	0	0	0	0	0	0	0	0	0	0	0	0	0	0
0	0	9-Feb-2021	15-Feb-2021	6	0	0	17	11	56	0	5	5	5	2	2	0	0	0	0	0	1	0	1	1	1	1	1	1	0	1	0	0	0	0	0	1	0	0	0	0	0	0	0	0	0	0	0	0	0	0	0	0	0	0	0	0
0	0	10-Feb-2021	15-Feb-2021	5	0	0	17	12	70	0	6	6	6	2	2	0	1	0	0	1	1	0	0	1	1	1	1	1	1	0	0	0	0	0	0	0	1	0	0	0	0	0	0	0	0	0	0	0	0	0	0	0	0	0	0	0
0	0	10-Feb-2021	15-Feb-2021	5	1	1	11	10	64	1	6	6	7	8	8	0	0	0	0	0	1	0	1	1	1	1	1	1	0	1	0	0	0	0	0	1	0	0	0	0	0	0	0	0	0	0	0	0	0	0	0	0	0	0	0	0
0	0	11-Feb-2021	15-Feb-2021	4	0	0	13	5	24	1	5	5	2	2	2	1	1	1	0	3	1	0	1	1	1	1	1	1	1	0	0	0	0	0	0	1	0	0	0	0	0	0	0	0	0	0	0	0	0	0	0	0	0	0	0	0
0	0	11-Feb-2021	15-Feb-2021	4	0	0	10	8	43	1	6	6	4	2	2	0	1	0	0	1	1	0	1	1	1	1	1	1	1	0	0	0	0	0	0	1	0	0	0	0	0	0	0	0	0	0	0	0	0	0	0	0	0	0	0	0
0	0	12-Feb-2021	15-Feb-2021	3	0	0	25	21	48	0	5	6	6	5	2	0	0	0	0	0	1	0	0	1	1	1	1	1	0	1	0	0	0	0	0	0	1	0	0	0	0	0	0	0	0	0	0	0	0	0	0	0	0	0	0	0
0	0	12-Feb-2021	15-Feb-2021	3	1	1	11	6	56	1	6	6	8	8	8	0	0	0	0	0	1	0	1	1	1	1	1	1	0	1	0	0	0	0	0	0	1	0	0	0	0	0	0	0	0	0	0	0	0	0	0	0	0	0	0	0
0	0	13-Feb-2021	15-Feb-2021	2	1	1	10	9	64	1	6	6	7	8	8	0	0	0	0	0	1	0	1	1	1	1	1	1	1	0	0	0	0	0	0	1	0	0	0	0	0	0	0	0	0	0	0	0	0	0	0	0	0	0	0	0
0	0	14-Feb-2021	15-Feb-2021	1	1	1	4	3	65	0	6	8	8	8	8	0	0	0	0	0	1	0	1	1	1	1	1	1	0	1	0	0	0	0	0	0	1	0	0	0	0	0	0	0	0	0	0	0	0	0	0	0	0	0	0	0
0	0	14-Feb-2021	15-Feb-2021	1	1	1	9	4	53	0	6	5	8	8	8	0	0	0	0	0	1	0	1	1	1	1	1	1	1	0	0	0	0	0	0	1	0	0	0	0	0	0	0	0	0	0	0	0	0	0	0	0	0	0	0	0
0	0	14-Feb-2021	15-Feb-2021	1	0	0	15	13	24	1	5	6	5	2	1	0	0	0	0	0	1	0	1	1	1	1	1	1	1	0	0	0	0	0	0	1	0	0	0	0	0	0	0	0	0	0	0	0	0	0	0	0	0	0	0	0
0	0	15-Feb-2021	15-Feb-2021	0	1	1	10	7	51	1	6	6	8	8	8	0	0	1	0	1	1	0	1	1	1	1	1	1	1	0	0	0	0	0	0	1	0	0	0	0	0	0	0	0	0	0	1	0	0	0	0	0	0	0	0	0
0	0	15-Feb-2021	15-Feb-2021	0	0	1	20	18	35	1	6	6	7	7	8	0	0	0	0	0	1	0	1	1	1	1	1	1	1	0	0	0	0	0	0	1	0	0	0	0	0	0	0	0	0	0	0	0	0	0	0	0	0	0	0	0
1	0	14-Feb-2021	15-Feb-2021	1	0	0	10	9	46	0	5	4	3	1	1	0	0	0	0	0	1	0	1	1	1	1	1	1	1	0	0	0	0	0	0	1	0	0	0	0	0	0	0	0	0	0	0	0	0	0	0	0	0	0	0	0
1	0	13-Feb-2021	15-Feb-2021	2	0	0	3	1	25	1	6	1	1	1	1	0	1	0	0	1	1	0	1	1	1	1	1	1	1	1	0	0	0	0	0	1	0	0	0	0	0	0	0	0	0	0	0	0	0	0	0	0	0	0	0	0
1	0	14-Feb-2021	15-Feb-2021	1	0	0	4	3	57	0	4	2	2	1	1	0	1	0	0	1	1	0	1	1	1	1	1	1	1	1	0	0	0	0	0	1	0	0	0	0	0	0	0	0	0	0	1	0	0	0	0	0	0	0	0	0
1	0	13-Feb-2021	15-Feb-2021	2	0	0	4	2	57	0	6	2	1	1	1	0	0	0	0	0	1	0	1	1	1	1	1	1	1	0	0	0	0	0	0	1	0	0	0	0	0	0	0	0	0	0	0	0	0	0	0	0	0	0	0	0
1	0	12-Feb-2021	15-Feb-2021	3	0	0	6	3	42	1	6	2	1	1	1	0	0	0	0	0	1	0	0	1	1	1	1	1	0	1	0	0	0	0	0	0	1	0	0	0	0	0	0	0	0	0	0	0	0	0	0	0	0	0	0	0
1	0	9-Feb-2021	15-Feb-2021	6	0	0	10	4	52	1	6	5	2	1	1	0	1	0	0	1	1	0	0	1	1	1	1	1	0	1	0	0	0	0	0	0	1	0	0	0	0	0	0	0	0	0	0	0	0	0	0	0	0	0	0	0
1	0	5-Feb-2021	15-Feb-2021	10	0	0	13	3	32	0	6	2	1	1	1	0	0	0	0	0	1	0	0	1	1	1	1	1	0	1	0	0	0	0	0	0	1	0	0	0	0	0	0	0	0	0	0	0	0	0	0	0	0	0	0	0
1	0	10-Feb-2021	15-Feb-2021	5	0	0	14	9	29	1	6	5	3	1	1	0	0	0	0	0	1	0	0	1	1	1	1	1	0	1	0	0	0	0	0	0	1	0	0	0	0	0	0	0	0	0	0	0	0	0	0	0	0	0	0	0
1	0	13-Feb-2021	15-Feb-2021	2	0	0	4	2	47	0	5	3	1	1	1	0	0	0	0	0	1	0	1	1	1	1	1	1	0	1	0	0	0	0	0	1	0	0	0	0	0	0	0	0	0	0	1	0	0	0	0	0	0	0	0	0
1	0	12-Feb-2021	15-Feb-2021	3	0	0	5	2	48	0	6	2	2	1	1	0	1	0	0	1	1	0	1	1	1	1	1	1	0	1	0	0	0	0	0	0	1	0	0	0	0	0	0	0	0	0	0	0	0	0	0	0	0	0	0	0
1	0	13-Feb-2021	15-Feb-2021	2	0	0	6	4	36	0	6	3	1	1	1	0	0	0	0	0	1	0	1	1	1	1	1	1	0	1	0	0	0	0	0	1	0	0	0	0	0	0	0	0	0	0	0	0	0	0	0	0	0	0	0	0
1	0	12-Feb-2021	15-Feb-2021	3	0	0	5	2	29	1	5	2	1	1	1	0	0	0	0	0	1	0	1	1	1	1	1	1	0	1	0	0	0	0	0	1	0	0	0	0	0	0	0	0	0	0	1	0	0	0	0	0	0	0	0	0
1	0	10-Feb-2021	15-Feb-2021	5	0	0	9	4	47	1	5	3	1	1	1	0	0	0	0	0	1	0	1	1	1	1	1	1	0	1	0	0	0	0	0	1	0	0	0	0	0	0	0	0	0	0	0	0	0	0	0	0	0	0	0	0
1	0	10-Feb-2021	15-Feb-2021	5	0	0	9	4	70	1	6	4	2	1	1	0	1	0	0	0	1	0	1	1	1	1	1	1	0	1	0	0	0	0	0	1	0	0	0	0	0	0	0	0	0	0	0	0	0	0	0	0	0	0	0	0
1	0	9-Feb-2021	15-Feb-2021	6	0	0	7	1	48	1	5	1	1	1	1	0	0	0	0	0	1	0	1	1	1	1	1	1	0	1	0	0	0	0	0	1	0	0	0	0	0	0	0	0	0	0	1	0	0	0	0	0	0	0	0	0
1	0	10-Feb-2021	15-Feb-2021	5	0	0	6	1	65	0	6	1	1	1	1	0	1	0	0	1	1	0	1	1	1	1	1	1	0	1	0	0	0	0	0	1	0	0	0	0	0	0	0	0	0	0	0	0	0	0	0	0	0	0	0	0
1	0	12-Feb-2021	15-Feb-2021	3	0	0	9	6	44	0	5	4	1	1	1	0	0	0	0	0	1	0	1	1	1	1	1	1	0	1	0	0	0	0	0	1	0	0	0	0	0	0	0	0	0	0	0	0	0	0	0	0	0	0	0	0
1	0	5-Feb-2021	15-Feb-2021	10	0	0	11	1	83	1	6	2	1	1	1	1	1	0	1	3	1	0	1	1	1	1	1	1	0	1	0	0	0	0	0	1	0	0	0	0	0	0	0	0	0	0	1	0	0	0	0	0	0	0	0	0
1	0	13-Feb-2021	15-Feb-2021	2	0	0	12	10	33	1	5	5	4	2	1	0	0	0	0	0	1	0	1	1	1	1	1	1	0	1	0	0	0	0	0	1	1	0	0	0	0	0	0	0	0	0	0	0	0	0	0	0	0	0	0	0
1	0	15-Feb-2021	15-Feb-2021	0	0	0	5	5	41	0	5	3	2	1	1	0	0	0	0	0	1	0	1	1	1	1	1	1	0	1	0	0	0	0	0	1	0	0	0	0	0	0	0	0	0	0	1	0	0	0	0	0	0	0	0	0
1	0	10-Feb-2021	15-Feb-2021	5	0	0	8	3	42	1	6	2	2	1	1	0	0	0	0	0	1	0	1	1	1	1	1	1	0	1	0	0	0	0	0	1	0	0	0	0	0	0	0	0	0	0	0	0	0	0	0	0	0	0	0	0
1	0	13-Feb-2021	15-Feb-2021	2	0	1	19	17	70	1	5	2	2	2	8	1	1	0	0	2	1	0	1	1	1	1	1	1	0	1	0	0	0	0	0	1	0	0	0	0	0	0	0	0	0	0	0	0	0	0	0	0	0	0	0	0
1	0	11-Feb-2021	15-Feb-2021	4	1	1	12	8	45	1	6	7	7	8	8	0	0	0	0	0	1	0	1	1	1	1	1	1	1	0	0	0	0	0	0	1	0	0	0	0	0	0	0	0	1	0	0	0	0	0	0	0	0	0	0	0
1	0	7-Feb-2021	15-Feb-2021	8	1	1	16	8	47	1	6	7	7	8	8	0	0	0	0	0	1	0	0	1	1	1	1	1	0	1	0	0	0	0	0	1	0	0	0	0	0	0	0	0	0	0	0	0	0	0	0	0	0	0	0	0
1	0	13-Feb-2021	15-Feb-2021	2	0	0	32	30	40	1	6	7	7	6	5	1	0	0	0	1	1	0	1	1	1	1	1	1	0	1	0	0	0	0	0	1	1	0	0	0	0	0	0	0	0	0	0	0	0	0	0	0	0	0	0	0
1	0	11-Feb-2021	15-Feb-2021	4	0	0	4	0	36	1	5	1	1	1	1	0	0	0	0	0	1	0	1	1	1	1	1	1	1	0	0	0	0	0	0	1	0	0	0	0	0	0	0	0	0	0	0	0	0	0	0	0	0	0	0	0
0	0	2-Feb-2021	16-Feb-2021	14	0	0	30	16	62	0	5	5	5	5	2	0	1	0	1	1	1	0	0	1	1	1	1	1	0	1	0	0	0	0	0	0	1	0	0	0	0	0	0	0	0	0	0	0	0	0	0	0	0	0	0	0
0	0	4-Feb-2021	16-Feb-2021	12	0	1	20	19	49	1	6	6	7	7	8	0	0	0	0	0	1	0	1	1	1	1	1	1	0	1	0	0	0	0	0	1	0	0	0	0	0	0	0	0	0	0	0	0	0	0	0	0	0	0	0	0
0	0	11-Feb-2021	16-Feb-2021	5	0	0	5	4	49	0	5	4	2	2	1	0	0	1	0	1	1	0	0	1	1	1	1	1	0	1	0	0	0	0	0	0	1	0	0	0	0	0	0	0	0	0	0	0	0	0	0	0	0	0	0	0
0	0	12-Feb-2021	16-Feb-2021	4	0	0	9	7	58	0	5	5	2	2	1	0	0	0	0	0	1	0	1	1	1	1	1	1	0	1	0	0	0	0	0	1	0	0	0	0	0	0	0	0	0	0	1	0	0	0	0	0	0	0	0	0
0	0	13-Feb-2021	16-Feb-2021	3	1	1	17	13	39	0	5	6	7	8	8	0	0	0	0	0	1	0	1	1	1	1	1	1	0	1	0	0	0	0	0	0	1	0	0	0	0	0	0	0	0	0	0	0	0	0	0	0	0	0	0	0
0	0	13-Feb-2021	16-Feb-2021	3	0	0	10	9	34	1	6	6	5	2	2	0	0	0	0	0	1	0	1	1	1	1	1	1	0	1	0	0	0	0	0	0	1	0	0	0	0	0	0	0	0	0	0	0	0	0	0	0	0	0	0	0
0	0	14-Feb-2021	16-Feb-2021	2	1	1	11	9	36	0	6	7	7	8	8	0	0	0	0	0	1	0	1	1	1	1	1	1	1	0	0	0	0	0	0	1	0	0	0	0	0	0	0	1	1	0	0	0	0	0	0	0	0	0	0	0
0	0	14-Feb-2021	16-Feb-2021	2	1	1	11	10	63	1	6	6	7	8	8	0	0	0	0	0	1	0	1	1	1	1	1	1	0	1	0	0	0	0	0	1	0	0	0	0	0	0	0	0	0	0	0	0	0	0	0	0	0	0	0	0
0	0	15-Feb-2021	16-Feb-2021	1	0	1	19	18	64	0	6	7	7	7	8	0	0	0	0	0	1	0	1	1	1	1	1	1	1	0	0	0	0	0	0	1	1	0	0	0	0	0	0	0	0	0	0	0	0	0	0	0	0	0	0	0
0	0	15-Feb-2021	16-Feb-2021	1	1	1	29	26	40	0	6	7	7	8	8	0	1	0	0	1	1	0	1	1	1	1	1	1	0	1	0	0	0	0	0	1	0	0	0	0	0	0	0	1	1	0	0	0	0	0	0	0	0	0	0	0
0	0	15-Feb-2021	16-Feb-2021	1	0	1	20	18	63	0	6	6	7	7	8	0	0	0	0	0	1	0	1	1	1	1	1	1	0	1	0	0	0	0	0	1	0	0	0	0	0	0	0	0	0	0	1	0	0	0	0	0	0	0	0	0
0	0	15-Feb-2021	16-Feb-2021	1	0	0	19	18	44	0	6	6	6	5	2	0	1	0	0	1	1	0	1	1	1	1	1	1	0	1	0	0	0	0	0	1	0	0	0	0	0	0	0	0	0	0	0	0	0	0	0	0	0	0	0	0
0	0	15-Feb-2021	16-Feb-2021	1	1	1	13	11	80	0	6	7	7	8	8	0	0	0	0	0	1	0	1	1	1	1	1	1	0	1	0	0	0	0	0	0	1	0	0	0	0	0	0	0	0	0	0	0	0	0	0	0	0	0	0	0
0	0	15-Feb-2021	16-Feb-2021	1	1	1	11	10	59	1	5	5	4	8	8	0	1	0	0	1	1	0	1	1	1	1	1	1	0	1	0	0	0	0	0	0	1	0	0	0	0	0	0	0	0	0	0	0	0	0	0	0	0	0	0	0
0	0	15-Feb-2021	16-Feb-2021	1	0	0	3	3	18	1	5	2	1	1	1	0	0	0	0	0	1	0	1	1	1	1	1	1	1	0	0	0	0	0	0	1	0	0	0	0	0	0	0	0	0	0	0	0	0	0	0	0	0	0	0	0
0	0	15-Feb-2021	16-Feb-2021	1	0	0	10	9	27	1	6	6	5	2	2	0	0	0	0	0	1	0	1	1	1	1	1	1	0	1	0	0	0	0	0	0	1	0	0	0	0	0	0	0	1	0	0	0	0	0	0	0	0	0	0	0
1	0	15-Feb-2021	16-Feb-2021	1	0	0	30	29	75	0	5	6	6	5	4	1	1	0	0	2	1	0	0	1	1	1	1	1	0	1	0	0	0	0	0	0	1	0	0	0	0	0	0	0	0	0	0	0	0	0	0	0	0	0	0	0
1	0	14-Feb-2021	16-Feb-2021	2	0	0	4	2	51	1	6	2	2	1	1	0	0	0	0	0	1	0	0	1	1	1	1	1	0	1	0	0	0	0	0	0	1	0	0	0	0	0	0	0	0	0	0	0	0	0	0	0	0	0	0	0
1	0	14-Feb-2021	16-Feb-2021	2	0	0	9	7	64	1	5	4	2	2	1	1	0	1	0	2	1	0	0	1	1	1	1	1	0	1	0	0	0	0	0	0	1	0	0	0	0	0	0	0	0	0	0	0	0	0	0	0	0	0	0	0
1	0	15-Feb-2021	16-Feb-2021	1	0	0	6	5	40	1	6	4	2	1	1	0	0	0	0	0	1	0	0	1	1	1	1	1	0	1	0	0	0	0	0	0	1	0	0	0	0	0	0	0	0	0	0	0	0	0	0	0	0	0	0	0
1	0	14-Feb-2021	16-Feb-2021	2	0	0	8	6	42	1	6	4	1	1	1	0	0	0	0	0	1	0	0	1	1	1	1	1	0	1	0	0	0	0	0	0	1	0	0	0	0	0	0	0	0	0	0	0	0	0	0	0	0	0	0	0
1	0	14-Feb-2021	16-Feb-2021	2	0	0	7	5	53	1	6	4	1	1	1	0	0	0	0	0	1	0	1	1	1	1	1	1	0	1	0	0	0	0	0	1	0	0	0	0	0	0	0	0	0	0	0	0	0	0	0	0	0	0	0	0
1	0	14-Feb-2021	16-Feb-2021	2	0	0	3	1	57	0	4	1	1	1	1	0	0	0	0	0	1	0	1	1	1	1	1	1	0	1	0	0	0	0	0	1	0	0	0	0	0	0	0	0	0	0	0	0	0	0	0	0	0	0	0	0
1	0	16-Feb-2021	16-Feb-2021	0	0	0	7	7	69	0	5	5	2	1	1	1	1	0	0	2	1	0	0	1	1	1	1	1	1	0	0	0	0	0	0	0	1	0	0	0	0	0	0	0	0	0	0	0	0	0	0	0	0	0	0	0
1	0	6-Feb-2021	16-Feb-2021	10	1	1	25	15	50	0	6	4	3	8	8	0	0	0	0	0	1	0	0	1	1	1	1	1	0	1	0	0	0	0	0	0	1	0	0	0	0	0	0	0	0	0	0	0	0	0	0	0	0	0	0	0
0	0	7-Feb-2021	17-Feb-2021	10	0	0	13	3	36	1	5	2	1	1	1	0	0	0	0	0	1	0	1	1	1	1	1	1	0	1	0	0	0	0	0	1	0	0	0	0	0	0	0	0	0	0	0	0	0	0	0	0	0	0	0	0
0	0	9-Feb-2021	17-Feb-2021	8	0	0	14	6	59	0	6	5	2	2	1	1	1	0	0	2	1	0	1	1	1	1	1	1	0	1	0	0	0	0	0	0	1	0	0	0	0	0	0	0	0	0	0	0	0	0	0	0	0	0	0	0
0	0	12-Feb-2021	17-Feb-2021	5	1	1	8	6	43	1	6	7	8	8	8	0	0	0	0	0	1	0	1	1	1	1	1	1	0	1	0	0	0	0	0	0	1	0	0	0	0	0	0	0	0	0	0	0	0	0	0	0	0	0	0	0
0	0	12-Feb-2021	17-Feb-2021	5	0	0	15	14	44	1	6	4	4	2	2	0	0	0	0	0	1	0	1	1	1	1	1	1	0	1	0	0	0	0	0	1	0	0	0	0	0	0	0	0	0	0	0	0	0	0	0	0	0	0	0	0
0	0	13-Feb-2021	17-Feb-2021	4	0	0	10	8	46	1	6	5	4	2	1	0	0	0	0	0	1	0	1	1	1	1	1	1	0	1	0	0	0	0	0	1	0	0	0	0	0	0	0	0	0	0	1	0	0	0	0	0	0	0	0	0
0	0	15-Feb-2021	17-Feb-2021	2	1	1	11	9	39	0	5	6	6	8	8	0	0	0	0	0	1	0	1	1	1	1	1	1	1	0	0	0	0	0	0	1	0	0	0	0	0	0	0	0	0	0	0	0	0	0	0	0	0	0	0	0
0	0	15-Feb-2021	17-Feb-2021	2	1	0	13	12	44	0	6	6	3	2	1	0	0	0	0	0	1	0	1	1	1	1	1	1	1	0	0	0	0	0	0	1	0	0	0	0	0	0	0	0	0	0	0	0	0	0	0	0	0	0	0	0
0	0	15-Feb-2021	17-Feb-2021	2	0	0	7	5	34	1	5	4	2	2	1	0	0	0	0	0	1	0	1	1	1	1	1	1	1	0	0	0	0	0	0	0	1	0	0	0	0	0	0	0	0	0	0	0	0	0	0	0	0	0	0	0
0	0	15-Feb-2021	17-Feb-2021	2	0	0	18	17	35	1	6	6	6	5	2	0	0	0	0	0	1	0	1	1	1	1	1	1	0	1	0	0	0	0	0	1	0	0	0	0	0	0	0	0	0	0	0	0	0	0	0	0	0	0	0	0
0	0	15-Feb-2021	17-Feb-2021	2	0	1	20	19	39	1	6	6	7	7	8	0	0	0	0	0	1	0	1	1	1	1	1	1	0	1	0	0	0	0	0	1	0	0	0	0	0	0	0	0	0	0	0	0	0	0	0	0	0	0	0	0
0	0	16-Feb-2021	17-Feb-2021	1	0	0	14	13	43	0	5	5	4	1	1	0	0	0	0	0	1	0	1	1	1	1	1	1	0	1	0	0	0	0	0	1	0	0	0	0	0	0	0	0	0	0	0	0	0	0	0	0	0	0	0	0
0	0	16-Feb-2021	17-Feb-2021	1	0	0	8	7	31	0	6	5	2	1	1	0	0	0	0	0	1	0	1	1	1	1	1	1	1	0	0	0	0	0	0	0	1	0	0	0	0	0	0	0	0	0	0	0	0	0	0	0	0	0	0	0
0	0	16-Feb-2021	17-Feb-2021	1	1	1	8	7	69	0	6	7	8	8	8	0	0	0	0	0	1	0	1	1	1	1	1	1	0	1	0	0	0	0	0	1	0	0	0	0	0	0	0	0	0	0	0	0	0	0	0	0	0	0	0	0
0	0	16-Feb-2021	17-Feb-2021	1	0	0	19	18	38	0	6	5	5	4	1	0	1	0	0	1	1	0	1	1	1	1	1	1	1	0	0	0	0	0	0	1	0	0	0	0	0	0	0	0	0	0	0	0	0	0	0	0	0	0	0	0
0	0	16-Feb-2021	17-Feb-2021	1	1	1	22	12	52	1	4	6	6	8	8	0	0	0	0	0	1	0	1	1	1	1	1	1	0	1	0	0	0	0	0	1	0	0	0	0	0	0	0	0	0	0	0	0	0	0	0	0	0	0	0	0
0	0	16-Feb-2021	17-Feb-2021	1	0	0	12	10	33	1	5	6	5	1	1	0	1	1	0	2	1	0	1	1	1	1	1	1	1	0	0	0	0	0	0	1	0	0	0	0	0	0	0	0	0	0	0	0	0	0	0	0	0	0	0	0
0	0	16-Feb-2021	17-Feb-2021	1	1	1	5	4	40	1	6	7	8	8	8	0	0	0	0	0	1	0	1	1	1	1	1	1	1	0	0	0	0	0	0	1	0	0	0	0	0	0	0	0	0	0	0	0	0	0	0	0	0	0	0	0
0	0	16-Feb-2021	17-Feb-2021	1	1	1	13	8	72	1	6	6	7	8	8	0	0	0	0	0	1	0	1	1	1	1	1	1	0	1	0	0	0	0	0	1	0	0	0	0	0	0	0	0	0	0	0	0	0	0	0	0	0	0	0	0
1	0	13-Feb-2021	17-Feb-2021	4	0	0	24	20	43	1	6	5	5	4	1	0	0	0	0	0	1	0	0	1	1	1	1	1	0	1	0	0	0	0	0	0	1	0	0	0	0	0	0	0	0	0	0	0	0	0	0	0	0	0	0	0
1	0	12-Feb-2021	17-Feb-2021	5	0	0	13	8	47	0	6	4	3	1	1	0	1	0	0	1	1	0	0	1	1	1	1	1	0	1	0	0	0	0	0	0	1	0	0	0	0	0	0	0	0	0	0	0	0	0	0	0	0	0	0	0
1	0	13-Feb-2021	17-Feb-2021	4	0	0	7	3	33	1	5	1	1	1	1	0	0	1	0	1	1	0	0	1	1	1	1	1	0	1	0	0	0	0	0	0	1	0	0	0	0	0	0	0	0	0	0	0	0	0	0	0	0	0	0	0
1	0	13-Feb-2021	17-Feb-2021	4	0	0	7	3	52	0	6	2	1	1	1	0	0	0	0	0	1	0	0	1	1	1	1	1	0	1	0	0	0	0	0	0	1	0	0	0	0	0	0	0	0	0	0	0	0	0	0	0	0	0	0	0
1	0	11-Feb-2021	17-Feb-2021	6	1	1	11	5	48	0	6	7	8	8	8	0	0	0	0	0	1	0	0	1	1	1	1	1	0	1	0	0	0	0	0	0	1	0	0	0	0	0	0	0	0	0	0	0	0	0	0	0	0	0	0	0
1	0	13-Feb-2021	17-Feb-2021	4	0	0	7	3	72	1	6	1	1	1	1	0	1	0	0	1	1	0	0	1	1	1	1	1	0	1	0	0	0	0	0	0	1	0	0	0	0	0	0	0	0	0	0	0	0	0	0	0	0	0	0	0
1	0	16-Feb-2021	17-Feb-2021	1	0	0	6	5	50	1	6	3	2	1	1	0	1	0	0	1	1	0	0	1	1	1	1	1	0	1	0	0	0	0	0	0	1	0	0	0	0	0	0	0	0	0	0	0	0	0	0	0	0	0	0	0
1	0	16-Feb-2021	17-Feb-2021	1	0	0	4	3	26	0	5	1	1	1	1	0	0	0	0	0	1	0	0	1	1	1	1	1	0	1	0	0	0	0	0	0	1	0	0	0	0	0	0	0	0	0	0	0	0	0	0	0	0	0	0	0
1	0	12-Feb-2021	17-Feb-2021	5	0	0	12	7	72	0	6	4	2	1	1	0	1	0	0	1	1	0	0	1	1	1	1	1	0	1	0	0	0	0	0	0	1	0	0	0	0	0	0	0	0	0	0	0	0	0	0	0	0	0	0	0
1	0	11-Feb-2021	17-Feb-2021	6	0	0	9	3	71	1	5	2	1	1	1	1	1	0	0	2	1	0	0	1	1	1	1	1	0	1	0	0	0	0	0	0	1	0	0	0	0	0	0	0	0	0	0	0	0	0	0	0	0	0	0	0
1	0	9-Feb-2021	17-Feb-2021	8	0	0	13	5	37	1	6	4	2	2	2	0	0	0	0	0	1	0	0	1	1	1	1	1	0	1	0	0	0	0	0	0	1	0	0	0	0	0	0	0	0	0	0	0	0	0	0	0	0	0	0	0
1	0	7-Feb-2021	17-Feb-2021	10	0	0	13	3	47	1	6	2	1	1	1	0	0	0	0	0	1	0	0	1	1	1	1	1	0	1	0	0	0	0	0	0	1	0	0	0	0	0	0	0	0	0	0	0	0	0	0	0	0	0	0	0
1	0	17-Feb-2021	17-Feb-2021	0	0	0	4	4	21	1	6	3	1	1	1	0	0	0	0	0	1	0	1	1	1	1	1	1	0	1	0	0	0	0	0	1	0	0	0	0	0	0	0	0	0	0	1	0	0	0	0	0	0	0	0	0
1	0	10-Feb-2021	17-Feb-2021	7	0	0	14	7	75	0	6	4	2	2	1	0	0	0	0	0	1	0	0	1	1	1	1	1	1	0	0	0	0	0	0	0	1	0	0	0	0	0	0	0	0	0	0	0	0	0	0	0	0	0	0	0
1	0	16-Feb-2021	17-Feb-2021	1	0	0	9	8	63	1	6	4	3	1	1	0	0	1	0	1	1	0	0	1	1	1	1	1	0	1	0	0	0	0	0	1	0	0	0	0	0	0	0	0	0	0	0	0	0	0	0	0	0	0	0	0
1	0	13-Feb-2021	17-Feb-2021	4	0	0	16	12	82	1	6	4	4	1	1	0	0	0	0	0	1	0	0	1	1	1	1	1	0	1	0	0	0	0	0	1	0	0	0	0	0	0	0	0	0	0	0	0	0	0	0	0	0	0	0	0
1	0	11-Feb-2021	17-Feb-2021	6	0	0	16	10	77	1	6	5	4	2	1	1	1	0	0	2	1	0	0	1	1	1	1	1	0	1	0	0	0	0	0	0	1	0	0	0	0	0	0	0	0	0	0	0	0	0	0	0	0	0	0	0
1	0	17-Feb-2021	17-Feb-2021	0	1	1	13	13	86	0	6	4	4	8	8	0	0	0	0	0	1	0	0	1	1	1	1	1	0	1	0	0	0	0	0	0	1	0	0	0	0	0	0	0	0	0	0	0	0	0	0	0	0	0	0	0
1	0	9-Feb-2021	17-Feb-2021	8	1	1	17	9	67	1	6	5	6	8	8	0	0	0	0	0	1	0	0	1	1	1	1	1	0	1	0	0	0	0	0	1	0	0	0	0	0	0	0	0	0	0	0	0	0	0	0	0	0	0	0	0
1	0	5-Feb-2021	17-Feb-2021	12	1	1	20	8	44	1	6	6	6	8	8	0	0	0	0	0	1	0	0	1	1	1	1	1	0	1	0	0	0	0	0	1	0	0	0	0	0	0	0	1	0	0	0	0	0	0	0	0	0	0	0	0
0	0	14-Feb-2021	18-Feb-2021	4	1	1	20	11	51	0	6	6	7	8	8	0	0	0	0	0	1	0	1	1	1	1	1	1	1	0	0	0	0	0	0	1	0	0	0	0	0	0	0	0	0	0	0	0	0	0	0	0	0	0	0	0
0	0	14-Feb-2021	18-Feb-2021	4	1	1	14	8	63	1	6	6	7	8	8	0	1	0	0	1	1	0	1	1	1	1	1	1	0	1	0	0	0	0	0	1	0	0	0	0	0	0	0	0	0	0	0	0	0	0	0	0	0	0	0	0
0	0	14-Feb-2021	18-Feb-2021	4	0	0	14	10	56	1	6	6	5	2	2	0	0	0	0	0	1	0	1	1	1	1	1	1	1	0	0	0	0	0	0	1	0	0	0	0	0	0	0	0	0	0	0	0	0	0	0	0	0	0	0	0
0	0	15-Feb-2021	18-Feb-2021	3	0	0	7	7	58	0	6	6	2	2	2	0	0	0	0	0	1	0	1	1	1	1	1	1	1	0	0	0	0	0	0	1	0	0	0	0	0	0	0	0	0	0	0	0	0	0	0	0	0	0	0	0
0	0	15-Feb-2021	18-Feb-2021	3	0	0	11	10	33	1	5	4	5	1	1	0	0	0	0	0	1	0	1	1	1	1	1	1	1	0	0	0	0	0	0	0	1	0	0	0	0	0	0	1	0	0	0	0	0	0	0	0	0	0	0	0
0	0	16-Feb-2021	18-Feb-2021	2	0	0	11	10	48	1	5	5	4	2	2	0	0	0	0	0	1	0	1	1	1	1	1	1	0	1	0	0	0	0	0	0	1	0	0	0	0	0	0	0	0	0	0	0	0	0	0	0	0	0	0	0
0	0	17-Feb-2021	18-Feb-2021	1	0	0	20	15	27	0	5	6	6	3	2	0	0	0	0	0	1	0	1	1	1	1	1	1	1	0	0	0	0	0	0	1	0	0	0	0	0	0	0	0	0	0	0	0	0	0	0	0	0	0	0	0
0	0	17-Feb-2021	18-Feb-2021	1	1	1	10	4	34	0	6	7	8	8	8	0	0	0	0	0	1	0	1	1	1	1	1	1	1	0	0	0	0	0	0	0	1	0	0	0	0	0	0	0	0	0	0	0	0	0	0	0	0	0	0	0
0	0	17-Feb-2021	18-Feb-2021	1	0	0	11	8	42	0	6	7	7	6	5	0	0	0	0	0	1	0	1	1	1	1	1	1	1	0	0	0	0	0	0	0	1	0	0	0	0	0	0	0	0	0	0	0	0	0	0	0	0	0	0	0
0	0	17-Feb-2021	18-Feb-2021	1	0	0	3	2	33	1	4	2	1	1	1	0	0	0	0	0	1	0	1	1	1	1	1	1	0	1	0	0	0	0	0	0	1	0	0	0	0	0	0	0	0	0	0	0	0	0	0	0	0	0	0	0
0	0	17-Feb-2021	18-Feb-2021	1	0	1	15	14	57	1	5	6	7	7	8	0	1	0	0	1	1	0	1	1	1	1	1	1	0	1	0	0	0	0	0	1	0	0	0	0	0	0	0	0	0	0	0	0	0	0	0	0	0	0	0	0
0	0	17-Feb-2021	18-Feb-2021	1	0	0	9	9	63	1	5	6	5	2	1	0	0	0	0	0	1	0	1	1	1	1	1	1	0	1	0	0	0	0	0	0	1	0	0	0	0	0	0	0	0	0	0	0	0	0	0	0	0	0	0	0
0	0	18-Feb-2021	18-Feb-2021	0	1	1	8	4	66	1	6	7	8	8	8	0	0	0	0	0	1	0	1	1	1	1	1	1	0	1	0	0	0	0	0	0	1	0	0	0	0	0	0	0	0	0	0	0	0	0	0	0	0	0	0	0
1	0	16-Feb-2021	18-Feb-2021	2	0	0	8	6	31	1	6	5	2	1	1	0	0	0	0	0	1	0	0	1	1	1	1	1	0	1	0	0	0	0	0	0	1	0	0	0	0	0	0	0	0	0	0	0	0	0	0	0	0	0	0	0
1	0	16-Feb-2021	18-Feb-2021	2	0	0	5	3	38	1	6	2	2	1	1	0	0	1	0	1	1	0	0	1	1	1	1	1	0	1	0	0	0	0	0	0	1	0	0	0	0	0	0	0	0	0	0	0	0	0	0	0	0	0	0	0
1	0	9-Feb-2021	18-Feb-2021	9	0	0	12	3	62	1	6	2	1	1	1	0	1	0	0	1	1	0	0	1	1	1	1	1	0	1	0	0	0	0	0	0	1	0	0	0	0	0	0	0	0	0	0	0	0	0	0	0	0	0	0	0
1	0	9-Feb-2021	18-Feb-2021	9	0	0	19	10	55	1	6	5	5	2	2	0	0	0	0	0	1	0	0	1	1	1	1	1	0	1	0	0	0	0	0	0	1	0	0	0	0	0	0	0	0	0	0	0	0	0	0	0	0	0	0	0
1	0	15-Feb-2021	18-Feb-2021	3	0	0	10	7	55	1	6	6	2	2	2	0	1	0	0	1	1	0	0	1	1	1	1	1	0	1	0	0	0	0	0	0	1	0	0	0	0	0	0	0	0	0	0	0	0	0	0	0	0	0	0	0
1	0	11-Feb-2021	18-Feb-2021	7	0	0	26	19	43	0	5	5	5	4	1	0	0	0	0	0	1	0	0	1	1	1	1	1	0	1	0	0	0	0	0	0	1	0	0	0	0	0	0	0	0	0	0	0	0	0	0	0	0	0	0	0
1	0	13-Feb-2021	18-Feb-2021	5	0	0	8	3	54	1	6	2	2	1	1	1	1	0	0	2	1	0	0	1	1	1	1	1	0	1	0	0	0	0	0	0	1	0	0	0	0	0	0	0	0	0	0	0	0	0	0	0	0	0	0	0
1	0	15-Feb-2021	18-Feb-2021	3	0	0	8	5	33	0	6	4	1	1	1	0	0	0	0	0	1	0	0	1	1	1	1	1	0	1	0	0	0	0	0	0	1	0	0	0	0	0	0	0	0	0	0	0	0	0	0	0	0	0	0	0
1	0	14-Feb-2021	18-Feb-2021	4	0	0	7	3	29	0	5	4	2	1	1	0	0	0	0	0	1	0	0	1	1	1	1	1	0	1	0	0	0	0	0	0	1	0	0	0	0	0	0	0	0	0	0	0	0	0	0	0	0	0	0	0
1	0	18-Feb-2021	18-Feb-2021	0	0	0	5	5	62	1	6	4	2	1	1	0	1	0	0	1	1	0	0	1	1	1	1	1	0	1	0	0	0	0	0	0	1	0	0	0	0	0	0	0	0	0	0	0	0	0	0	0	0	0	0	0
1	0	16-Feb-2021	18-Feb-2021	2	0	0	4	2	45	0	6	1	1	1	1	0	0	0	0	0	1	0	0	1	1	1	1	1	0	1	0	0	0	0	0	0	1	0	0	0	0	0	0	0	0	0	0	0	0	0	0	0	0	0	0	0
1	0	17-Feb-2021	18-Feb-2021	1	0	0	5	4	51	0	6	3	1	1	1	0	0	0	0	0	1	0	0	1	1	1	1	1	0	1	0	0	0	0	0	0	1	0	0	0	0	0	0	0	0	0	0	0	0	0	0	0	0	0	0	0
1	0	15-Feb-2021	18-Feb-2021	3	0	0	8	5	56	1	6	5	2	1	1	0	1	0	0	1	1	0	0	1	1	1	1	1	0	1	0	0	0	0	0	0	1	0	0	0	0	0	0	0	0	0	0	0	0	0	0	0	0	0	0	0
1	0	15-Feb-2021	18-Feb-2021	3	0	0	11	8	69	1	6	5	4	2	2	1	1	0	0	2	1	0	0	1	1	1	1	1	0	1	0	0	0	0	0	0	1	0	0	0	0	0	0	0	0	0	0	0	0	0	0	0	0	0	0	0
1	0	16-Feb-2021	18-Feb-2021	2	0	0	5	3	78	1	6	2	2	1	1	0	1	1	0	2	1	0	0	1	1	1	1	1	0	1	0	0	0	0	0	0	1	0	0	0	0	0	0	0	0	0	0	0	0	0	0	0	0	0	0	0
1	0	16-Feb-2021	18-Feb-2021	2	0	0	6	4	47	1	6	3	1	1	1	0	0	0	0	0	1	0	0	1	1	1	1	1	0	1	0	0	0	0	0	0	1	0	0	0	0	0	0	0	0	0	0	0	0	0	0	0	0	0	0	0
1	0	13-Feb-2021	18-Feb-2021	5	0	0	19	14	61	1	6	6	6	2	2	1	0	0	0	1	1	0	0	1	1	1	1	1	0	1	0	0	0	0	0	0	1	0	0	0	0	0	0	0	0	0	0	0	0	0	0	0	0	0	0	0
1	0	4-Feb-2021	18-Feb-2021	14	0	0	18	4	78	0	5	3	2	1	1	0	1	0	0	1	1	0	0	1	1	1	1	1	0	1	0	0	0	0	0	1	0	0	0	0	0	0	0	0	0	0	0	0	0	0	0	0	0	0	0	0
1	0	15-Feb-2021	18-Feb-2021	3	1	1	17	14	58	0	6	4	3	8	8	0	0	1	0	1	1	0	0	1	1	1	1	1	0	1	0	0	0	0	0	0	1	0	0	0	0	0	0	0	0	0	0	0	0	0	0	0	0	0	0	0
1	0	16-Feb-2021	18-Feb-2021	2	1	1	13	11	73	1	6	4	2	8	8	0	1	1	0	2	1	0	0	1	1	1	1	1	0	1	0	0	0	0	0	0	1	0	0	0	0	0	0	0	0	0	0	0	0	0	0	0	0	0	0	0
0	0	11-Feb-2021	19-Feb-2021	8	1	1	11	6	55	0	6	7	8	8	8	0	0	0	0	0	1	0	1	1	1	1	1	1	0	1	0	0	0	0	0	1	0	0	0	0	0	0	0	0	0	0	0	0	0	0	0	0	0	0	0	0
0	0	11-Feb-2021	19-Feb-2021	8	1	1	18	9	65	1	6	6	6	8	8	0	0	0	0	0	1	0	1	1	1	1	1	1	0	1	0	0	0	0	0	1	0	0	0	0	0	0	0	1	0	0	0	0	0	0	0	0	0	0	0	0
0	0	13-Feb-2021	19-Feb-2021	6	0	1	21	15	38	0	6	6	5	6	8	0	0	0	0	0	1	0	1	1	1	1	1	1	1	0	0	0	0	0	0	1	0	0	0	0	0	0	0	0	0	0	0	0	0	0	0	0	0	0	0	0
0	0	14-Feb-2021	19-Feb-2021	5	0	0	20	16	37	0	6	5	5	3	2	0	0	0	0	0	1	0	1	1	1	1	1	1	1	0	0	0	0	0	0	1	0	0	0	0	0	0	0	0	0	0	0	0	0	0	0	0	0	0	0	0
0	0	17-Feb-2021	19-Feb-2021	2	0	0	6	4	72	0	5	4	2	2	2	1	1	1	0	3	1	0	1	1	1	1	1	1	1	0	0	0	0	0	0	1	0	0	0	0	0	0	0	0	0	0	0	0	0	0	0	0	0	0	0	0
0	0	17-Feb-2021	19-Feb-2021	2	1	1	4	3	72	0	6	8	8	8	8	0	0	0	0	0	1	0	1	1	1	1	1	1	0	1	0	0	0	0	0	1	0	0	0	0	0	0	0	0	0	0	0	0	0	0	0	0	0	0	0	0
0	0	17-Feb-2021	19-Feb-2021	2	1	1	10	9	62	1	5	6	7	8	8	0	0	0	0	0	1	0	1	1	1	1	1	1	1	0	0	0	0	0	0	1	0	0	0	0	0	0	0	0	0	0	0	0	0	0	0	0	0	0	0	0
0	0	17-Feb-2021	19-Feb-2021	2	1	1	15	12	59	1	5	7	7	8	8	0	0	0	0	0	1	0	1	1	1	1	1	1	1	0	0	0	0	0	0	0	1	0	0	0	0	0	0	0	0	0	0	0	0	0	0	0	0	0	0	0
0	0	18-Feb-2021	19-Feb-2021	1	0	0	10	9	46	0	5	5	4	2	2	0	0	1	0	1	1	0	1	1	1	1	1	1	0	1	0	0	0	0	0	1	0	0	0	0	0	0	0	0	0	0	0	0	0	0	0	0	0	0	0	0
0	0	18-Feb-2021	19-Feb-2021	1	1	1	11	8	70	0	6	6	6	8	8	0	0	0	0	0	1	0	1	1	1	1	1	1	1	0	0	0	0	0	0	1	0	0	0	0	0	0	0	0	0	0	0	0	0	0	0	0	0	0	0	0
0	0	18-Feb-2021	19-Feb-2021	1	0	0	5	5	47	1	5	4	2	2	1	0	0	0	0	0	1	0	1	1	1	1	1	1	1	0	0	0	0	0	0	1	0	0	0	0	0	0	0	0	0	0	0	0	0	0	0	0	0	0	0	0
0	0	19-Feb-2021	19-Feb-2021	0	1	1	15	13	59	1	6	7	7	8	8	0	0	0	0	0	1	0	1	1	1	1	1	1	1	0	0	0	0	0	0	1	0	0	0	0	0	0	0	0	0	0	0	0	0	0	0	0	0	0	0	0
1	0	17-Feb-2021	19-Feb-2021	2	0	0	6	4	27	1	6	3	2	2	1	0	0	0	0	0	1	0	1	1	1	1	1	1	1	0	0	0	0	0	0	1	0	0	0	0	0	0	0	0	0	0	1	1	0	0	0	0	0	0	0	0
1	0	18-Feb-2021	19-Feb-2021	1	0	0	7	6	30	1	6	3	2	1	1	0	0	0	0	0	1	0	1	1	1	1	1	1	1	0	0	0	0	0	0	1	0	0	0	0	0	0	0	0	0	0	0	0	0	0	0	0	0	0	0	0
1	0	17-Feb-2021	19-Feb-2021	1	0	0	7	5	47	0	5	4	4	4	2	1	1	0	0	2	1	0	1	1	1	1	1	1	1	0	0	0	0	0	0	1	0	0	0	0	0	0	0	0	0	0	0	0	0	0	0	0	0	0	0	0
1	0	17-Feb-2021	19-Feb-2021	2	0	0	8	6	31	1	6	4	2	1	1	0	0	0	0	0	1	0	1	1	1	1	1	1	1	0	0	0	0	0	0	1	0	0	0	0	0	0	0	0	0	0	1	0	0	0	0	0	0	0	0	0
1	0	8-Feb-2021	19-Feb-2021	11	0	0	17	6	29	1	5	4	1	1	1	0	0	0	0	0	1	0	0	1	1	1	1	1	0	1	0	0	0	0	0	0	1	0	0	0	0	0	0	0	0	0	0	0	0	0	0	0	0	0	0	0
1	0	18-Feb-2021	19-Feb-2021	1	0	0	6	5	54	1	6	4	2	1	1	1	1	0	0	2	1	0	0	1	1	1	1	1	0	1	0	0	0	0	0	0	1	0	0	0	0	0	0	0	0	0	0	0	0	0	0	0	0	0	0	0
1	0	17-Feb-2021	19-Feb-2021	2	0	0	11	9	49	1	6	4	3	1	1	0	0	0	0	0	1	0	0	1	1	1	1	1	0	1	0	0	0	0	0	0	1	0	0	0	0	0	0	0	0	0	0	0	0	0	0	0	0	0	0	0
1	0	17-Feb-2021	19-Feb-2021	2	0	0	8	6	52	1	6	3	1	1	1	0	0	0	1	1	1	0	0	1	1	1	1	1	0	1	0	0	0	0	0	0	1	0	0	0	0	0	0	0	0	0	0	0	0	0	0	0	0	0	0	0
1	0	18-Feb-2021	19-Feb-2021	1	0	0	8	7	46	0	6	4	2	1	1	0	0	0	0	0	1	0	0	1	1	1	1	1	0	1	0	0	0	0	0	0	1	0	0	0	0	0	0	0	0	0	0	0	0	0	0	0	0	0	0	0
1	0	14-Feb-2021	19-Feb-2021	5	1	1	14	9	49	1	6	5	5	8	8	0	0	0	0	0	1	0	0	1	1	1	1	1	0	1	0	0	0	0	0	0	1	0	0	0	0	0	0	0	0	0	0	0	0	0	0	0	0	0	0	0
0	0	8-Feb-2021	20-Feb-2021	12	0	0	12	9	47	1	6	6	5	2	2	0	0	0	0	0	1	0	1	1	1	1	1	1	1	0	0	0	0	0	0	1	0	0	0	0	0	0	0	0	0	0	0	0	0	0	0	0	0	0	0	0
0	0	11-Feb-2021	20-Feb-2021	9	0	0	11	11	57	1	4	6	6	2	1	1	0	0	0	1	1	0	1	1	1	1	1	1	0	1	0	0	0	0	0	1	0	0	0	0	0	0	0	0	0	0	0	0	0	0	0	0	0	0	0	0
0	0	12-Feb-2021	20-Feb-2021	8	1	1	15	14	58	1	5	7	7	8	8	0	1	1	0	2	1	0	1	1	1	1	1	1	2	1	0	0	0	0	0	1	0	0	0	0	0	0	0	0	0	0	0	0	0	0	0	0	0	0	0	0
0	0	14-Feb-2021	20-Feb-2021	6	0	0	10	6	33	0	6	4	2	2	2	1	1	0	0	2	1	0	1	1	1	1	1	1	1	0	0	0	0	0	0	1	0	0	0	0	0	0	0	0	0	0	0	0	0	0	0	0	0	0	0	0
0	0	17-Feb-2021	20-Feb-2021	3	1	1	13	11	39	1	6	6	6	8	8	0	1	0	1	2	1	0	1	1	1	1	1	1	0	1	0	0	0	0	0	1	0	0	0	0	0	0	0	0	0	0	0	0	0	0	0	0	0	0	0	0
0	0	17-Feb-2021	20-Feb-2021	3	0	0	8	7	77	1	6	4	2	1	1	1	0	0	0	1	1	0	1	1	1	1	1	1	0	1	0	0	0	0	0	1	0	0	0	0	0	0	0	1	0	0	0	0	0	0	0	0	0	0	0	0
0	0	18-Feb-2021	20-Feb-2021	2	1	1	14	13	37	1	5	6	7	8	8	0	0	0	0	0	1	0	1	1	1	1	1	1	1	0	0	0	0	0	0	1	0	0	0	0	0	0	0	0	0	0	0	0	0	0	0	0	0	0	0	0
0	0	18-Feb-2021	20-Feb-2021	2	1	1	7	6	52	1	6	6	8	8	8	1	1	0	0	2	1	0	1	1	1	1	1	1	0	1	0	0	0	0	0	1	0	0	0	0	0	0	0	0	0	0	0	0	0	0	0	0	0	0	0	0
0	0	19-Feb-2021	20-Feb-2021	1	1	1	13	11	64	0	5	6	6	8	8	0	0	0	0	0	1	0	1	1	1	1	1	1	1	0	0	0	0	0	0	1	0	0	0	0	0	0	0	0	1	0	0	0	0	0	0	0	0	0	0	0
0	0	19-Feb-2021	20-Feb-2021	1	0	0	9	6	41	0	5	4	2	2	2	0	0	0	0	0	1	0	1	1	1	1	1	1	1	0	0	0	0	0	0	1	0	0	0	0	0	0	0	0	0	0	0	0	0	0	0	0	0	0	0	0
0	0	19-Feb-2021	20-Feb-2021	1	1	1	2	1	38	0	6	8	8	8	8	0	1	0	0	1	1	0	1	1	1	1	1	1	1	0	0	0	0	0	0	1	0	0	0	0	0	0	0	0	0	0	0	0	0	0	0	0	0	0	0	0
0	0	19-Feb-2021	20-Feb-2021	1	0	0	7	6	38	1	4	5	2	1	1	0	0	0	0	0	1	0	1	1	1	1	1	1	0	1	0	0	0	0	0	1	0	0	0	0	0	0	0	0	0	0	0	0	0	0	0	0	0	0	0	0
0	0	19-Feb-2021	20-Feb-2021	1	0	0	3	2	28	1	5	2	2	1	1	0	0	0	0	0	1	0	1	1	1	1	1	1	1	0	0	0	0	0	0	1	0	0	0	0	0	0	0	0	0	0	0	0	0	0	0	0	0	0	0	0
0	0	19-Feb-2021	20-Feb-2021	1	1	1	10	5	78	1	6	6	8	8	8	0	0	0	0	0	1	0	1	1	1	1	1	1	1	0	0	0	0	0	0	1	0	0	0	0	0	0	0	0	0	0	0	0	0	0	0	0	0	0	0	0
0	0	19-Feb-2021	20-Feb-2021	1	1	1	11	8	58	1	6	5	7	8	8	0	0	0	0	0	1	0	1	1	1	1	1	1	1	0	0	0	0	0	0	1	0	0	0	0	0	0	0	0	0	0	0	0	0	0	0	0	0	0	0	0
0	0	19-Feb-2021	20-Feb-2021	1	1	1	8	7	54	1	6	7	8	8	8	0	0	0	0	0	1	0	1	1	1	1	1	1	1	0	0	0	0	0	0	1	0	0	0	0	0	0	0	0	0	0	0	0	0	0	0	0	0	0	0	0
1	0	16-Feb-2021	20-Feb-2021	4	0	0	35	31	42	1	6	3	1	1	1	1	0	1	0	2	1	0	1	1	1	1	1	1	1	0	0	0	0	0	0	1	0	0	0	0	0	0	0	0	0	0	0	0	0	0	0	0	0	0	0	0
1	0	18-Feb-2021	20-Feb-2021	2	0	0	6	4	43	1	6	3	1	1	1	0	1	0	0	1	1	0	1	1	1	1	1	1	1	0	0	0	0	0	0	1	0	0	0	0	0	0	0	0	0	0	0	0	0	0	0	0	0	0	0	0
1	0	11-Feb-2021	20-Feb-2021	9	0	0	17	8	41	1	6	3	3	1	1	0	0	0	0	0	1	0	1	1	1	1	1	1	0	0	0	0	0	0	0	1	0	0	0	0	0	0	0	0	0	0	0	0	0	0	0	0	0	0	0	0
1	0	19-Feb-2021	20-Feb-2021	1	0	0	6	5	36	0	6	4	2	2	1	0	1	0	0	1	1	0	1	1	1	1	1	1	1	0	0	0	0	0	0	1	0	0	0	0	0	0	0	0	0	0	1	0	0	0	0	0	0	0	0	0
1	0	19-Feb-2021	20-Feb-2021	1	0	0	5	4	81	0	6	3	1	1	1	1	1	0	0	2	1	0	0	1	1	1	1	1	0	1	0	0	0	0	0	0	1	0	0	0	0	0	0	0	0	0	0	0	0	0	0	0	0	0	0	0
1	0	20-Feb-2021	20-Feb-2021	0	0	0	5	5	58	0	5	4	1	1	1	0	0	1	0	1	1	0	1	1	1	1	1	1	0	1	0	0	0	0	0	1	0	0	0	0	0	0	0	0	0	0	1	0	0	0	0	0	0	0	0	0
1	0	19-Feb-2021	20-Feb-2021	1	0	0	6	5	44	1	6	3	1	1	1	0	0	0	0	0	1	0	0	1	1	1	1	1	0	1	0	0	0	0	0	1	0	0	0	0	0	0	0	0	0	0	0	0	0	0	0	0	0	0	0	0
1	0	19-Feb-2021	20-Feb-2021	1	0	0	7	6	39	1	5	3	2	1	1	0	0	0	0	0	1	0	0	1	1	1	1	1	0	1	0	0	0	0	0	1	0	0	0	0	0	0	0	0	0	0	0	0	0	0	0	0	0	0	0	0
1	0	19-Feb-2021	20-Feb-2021	1	0	0	6	5	42	1	5	3	1	1	1	0	0	0	0	0	1	0	0	1	1	1	1	1	0	1	0	0	0	0	0	1	0	0	0	0	0	0	0	0	0	0	0	0	0	0	0	0	0	0	0	0
1	0	12-Feb-2021	20-Feb-2021	8	0	0	11	3	45	0	5	2	1	1	1	0	0	0	0	0	1	0	0	1	1	1	1	1	0	1	0	0	0	0	0	1	0	0	0	0	0	0	0	0	0	0	0	0	0	0	0	0	0	0	0	0
1	0	20-Feb-2021	20-Feb-2021	0	0	0	3	3	46	0	6	2	1	1	1	0	0	0	0	0	1	0	0	1	1	1	1	1	0	1	0	0	0	0	0	1	0	0	0	0	0	0	0	0	0	0	0	0	0	0	0	0	0	0	0	0
1	0	11-Feb-2021	20-Feb-2021	9	0	0	22	13	64	1	6	5	4	2	1	0	1	0	0	1	1	0	1	1	1	1	1	1	1	0	0	0	0	0	0	1	0	0	0	0	0	0	0	0	0	0	0	0	0	0	0	0	0	0	0	1
1	0	17-Feb-2021	20-Feb-2021	3	1	1	8	5	45	1	6	7	8	8	8	0	0	0	0	0	1	0	0	1	1	1	1	1	0	1	0	0	0	0	0	0	1	0	0	0	0	0	0	0	0	0	0	0	0	0	0	0	0	0	0	0
0	0	3-Feb-2021	21-Feb-2021	18	0	0	17	16	40	1	6	5	4	4	2	0	0	0	0	0	1	0	1	1	1	1	1	1	1	0	0	0	0	0	0	1	0	0	0	0	0	0	0	0	0	0	0	0	0	0	0	0	0	0	0	0
0	0	15-Feb-2021	21-Feb-2021	6	0	0	14	8	36	0	5	5	4	2	2	0	0	0	0	0	1	0	1	1	1	1	1	1	0	1	0	0	0	0	0	1	0	0	0	0	0	0	0	0	0	0	0	0	0	0	0	0	0	0	0	0
0	0	16-Feb-2021	21-Feb-2021	5	0	0	8	3	37	0	6	2	2	2	2	0	0	0	0	0	1	0	1	1	1	1	1	1	0	1	0	0	0	0	0	0	1	0	0	0	0	0	0	0	0	0	0	0	0	0	0	0	0	0	0	0
0	0	16-Feb-2021	21-Feb-2021	5	0	0	13	12	52	1	5	4	4	2	2	0	0	0	0	0	1	0	1	1	1	1	1	1	1	0	0	0	0	0	0	1	0	0	0	0	0	0	0	0	0	0	0	0	0	0	0	0	0	0	0	0
0	0	17-Feb-2021	21-Feb-2021	4	1	1	8	7	59	1	6	7	8	8	8	0	1	0	0	1	1	0	1	1	1	1	1	1	1	0	0	0	0	0	0	1	0	0	0	0	0	0	0	0	0	0	0	0	0	0	0	0	0	0	0	0
0	0	17-Feb-2021	21-Feb-2021	4	1	1	5	3	53	1	6	8	8	8	8	0	0	0	0	0	1	0	1	1	1	1	1	1	1	0	0	0	0	0	0	1	0	0	0	0	0	0	0	0	0	0	0	0	0	0	0	0	0	0	0	0
0	0	18-Feb-2021	21-Feb-2021	3	0	0	12	10	32	0	6	5	5	2	1	0	0	0	0	0	1	0	1	1	1	1	1	1	1	0	0	0	0	0	0	1	0	0	0	0	0	0	0	0	0	0	0	0	0	0	0	0	0	0	0	0
0	0	19-Feb-2021	21-Feb-2021	2	1	1	10	8	64	0	6	7	7	8	8	0	1	1	0	2	1	0	1	1	1	1	1	1	0	1	0	0	0	0	0	1	0	0	0	0	0	0	0	0	1	0	0	0	0	0	0	0	0	0	0	0
0	0	19-Feb-2021	21-Feb-2021	2	0	0	4	3	33	1	5	2	1	1	1	0	0	0	0	0	1	0	1	1	1	1	1	1	2	1	0	0	0	0	0	1	0	0	0	0	0	0	0	0	0	0	0	0	0	0	0	0	0	0	0	0
0	0	19-Feb-2021	21-Feb-2021	2	0	0	19	17	64	1	5	5	5	5	2	0	0	0	0	0	1	0	1	1	1	1	1	1	1	0	0	0	0	0	0	1	0	0	0	0	0	0	0	0	0	0	0	0	0	0	0	0	0	0	0	0
0	0	20-Feb-2021	21-Feb-2021	1	1	1	5	4	40	0	6	7	8	8	8	0	0	0	0	0	1	0	1	1	1	1	1	1	1	0	0	0	0	0	0	1	0	0	0	0	0	0	0	0	0	0	0	0	0	0	0	0	0	0	0	0
0	0	20-Feb-2021	21-Feb-2021	1	0	0	6	5	22	1	5	5	2	1	1	0	0	0	0	0	1	0	1	1	1	1	1	1	1	0	0	0	0	0	0	2	1	0	0	0	0	0	0	0	0	0	0	0	0	0	0	0	0	0	0	0
0	0	20-Feb-2021	21-Feb-2021	1	0	0	17	15	34	1	6	5	5	3	1	0	1	0	0	1	1	0	1	1	1	1	1	1	0	1	0	0	0	0	0	1	0	0	0	0	0	0	0	0	1	0	0	0	0	0	0	0	0	0	0	0
0	0	20-Feb-2021	21-Feb-2021	1	0	0	19	18	43	1	6	6	6	6	2	0	0	0	0	0	1	0	1	1	1	1	1	1	1	0	0	0	0	0	0	1	0	0	0	0	0	0	0	0	0	0	0	0	0	0	0	0	0	0	0	0
1	0	21-Feb-2021	21-Feb-2021	1	0	0	5	5	39	1	6	2	2	1	1	0	0	0	0	0	1	0	1	1	1	1	1	1	1	0	0	0	0	0	0	1	0	0	0	0	0	0	0	0	0	0	0	0	0	0	0	0	0	0	0	0
1	0	19-Feb-2021	21-Feb-2021	2	0	0	12	10	65	1	6	4	3	2	1	0	0	0	0	0	1	0	1	1	1	1	1	1	1	0	0	0	0	0	0	1	0	0	0	0	0	0	0	0	0	0	0	0	0	0	0	0	0	0	0	0
1	0	18-Feb-2021	21-Feb-2021	3	0	0	6	3	39	1	6	2	1	1	1	0	0	0	0	0	1	0	1	1	1	1	1	1	1	0	0	0	0	0	0	1	0	0	0	0	0	0	0	0	1	0	0	0	0	0	0	0	0	0	0	0
1	0	19-Feb-2021	21-Feb-2021	2	0	0	6	4	59	1	6	3	1	1	1	0	0	0	0	0	1	0	0	1	1	1	1	1	0	1	0	0	0	0	0	0	1	0	0	0	0	0	0	0	0	0	0	0	0	0	0	0	0	0	0	0
1	0	21-Feb-2021	21-Feb-2021	0	0	0	2	2	45	0	5	2	1	1	1	0	0	0	0	0	1	0	0	1	1	1	1	1	0	1	0	0	0	0	0	0	1	0	0	0	0	0	0	0	0	0	0	0	0	0	0	0	0	0	0	0
1	0	19-Feb-2021	21-Feb-2021	2	0	0	4	2	69	1	5	2	1	1	1	0	0	0	0	0	1	0	0	1	1	1	1	1	0	1	0	0	0	0	0	0	1	0	0	0	0	0	0	0	0	0	0	0	0	0	0	0	0	0	0	0
1	0	19-Feb-2021	21-Feb-2021	2	0	0	7	5	52	0	6	4	2	1	1	0	0	0	0	0	1	0	0	1	1	1	1	1	0	1	0	0	0	0	0	0	1	0	0	0	0	0	0	0	0	0	0	0	0	0	0	0	0	0	0	0
1	0	19-Feb-2021	21-Feb-2021	2	0	0	6	4	73	0	6	3	2	1	1	0	0	0	0	0	1	0	0	1	1	1	1	1	0	1	0	0	0	0	0	0	1	0	0	0	0	0	0	0	0	0	0	0	0	0	0	0	0	0	0	0
1	0	18-Feb-2021	21-Feb-2021	3	0	0	7	4	53	0	5	4	1	1	1	0	1	0	0	1	1	0	0	1	1	1	1	1	0	1	0	0	0	0	0	0	1	0	0	0	0	0	0	0	0	0	0	0	0	0	0	0	0	0	0	0
1	0	20-Feb-2021	21-Feb-2021	1	0	0	5	4	39	1	6	3	2	2	1	0	0	0	0	0	1	0	0	1	1	1	1	1	0	1	0	0	0	0	0	0	1	0	0	0	0	0	0	0	0	0	0	0	0	0	0	0	0	0	0	0
1	0	21-Feb-2021	21-Feb-2021	0	0	0	2	2	61	1	5	2	1	1	1	0	1	0	0	1	1	0	0	1	1	1	1	1	0	1	0	0	0	0	0	0	1	0	0	0	0	0	0	0	0	0	0	0	0	0	0	0	0	0	0	0
1	0	10-Feb-2021	21-Feb-2021	11	0	0	14	3	27	0	4	2	1	1	1	0	0	1	0	1	1	0	0	1	1	1	1	1	0	1	0	0	0	0	0	1	0	0	0	0	0	0	0	0	0	0	0	0	0	0	0	0	0	0	0	0
1	0	18-Feb-2021	21-Feb-2021	3	0	0	12	9	64	0	6	6	6	2	1	1	1	0	0	2	1	0	0	1	1	1	1	1	0	1	0	0	0	0	0	1	0	0	0	0	0	0	0	0	0	0	0	0	0	0	0	0	0	0	0	0
1	0	18-Feb-2021	21-Feb-2021	3	0	0	10	7	75	1	5	4	1	1	1	0	1	0	0	1	1	0	0	1	1	1	1	1	0	1	0	0	0	0	0	1	0	0	0	0	0	0	0	0	0	0	0	0	0	0	0	0	0	0	0	0
1	0	18-Feb-2021	21-Feb-2021	3	0	0	4	1	52	0	6	1	1	1	1	0	0	1	0	1	1	0	0	1	1	1	1	1	0	1	0	0	0	0	0	1	0	0	0	0	0	0	0	0	0	0	0	0	0	0	0	0	0	0	0	0
1	0	7-Feb-2021	21-Feb-2021	14	0	0	19	5	47	0	6	5	2	1	1	0	0	0	0	0	1	0	0	1	1	1	1	1	0	1	0	0	0	0	0	1	0	0	0	0	0	0	0	0	0	0	0	0	0	0	0	0	0	0	0	0
0	0	14-Feb-2021	22-Feb-2021	8	0	0	12	4	44	1	5	4	2	1	1	0	0	0	0	0	1	0	1	1	1	1	1	1	0	1	0	0	0	0	0	0	1	0	0	0	0	0	0	0	0	0	0	0	0	0	0	0	0	0	0	0
0	0	17-Feb-2021	22-Feb-2021	5	0	0	6	4	31	1	6	4	2	2	1	0	0	0	0	0	1	0	1	1	1	1	1	1	1	0	0	0	0	0	0	1	0	0	0	0	0	0	0	0	0	0	0	0	0	0	0	0	0	0	0	0
0	0	19-Feb-2021	22-Feb-2021	3	1	1	8	5	60	0	6	7	8	8	8	0	0	0	0	0	1	0	1	1	1	1	1	1	1	0	0	0	0	0	0	1	0	0	0	0	0	0	0	0	0	0	0	0	0	0	0	0	0	0	0	0
0	0	19-Feb-2021	22-Feb-2021	3	0	0	9	8	50	1	5	5	4	2	2	1	1	0	0	2	1	0	1	1	1	1	1	1	1	0	0	0	0	0	0	1	0	0	0	0	0	0	0	0	0	0	0	0	0	0	0	0	0	0	0	0
0	0	19-Feb-2021	22-Feb-2021	3	1	1	4	3	65	1	6	8	8	8	8	0	0	0	0	0	1	0	1	1	1	1	1	1	1	0	0	0	0	0	0	0	1	0	0	0	0	0	0	0	0	0	0	0	0	0	0	0	0	0	0	0
0	0	20-Feb-2021	22-Feb-2021	2	0	0	12	9	36	1	5	5	5	1	1	0	0	0	0	0	1	0	1	1	1	1	1	1	1	0	0	0	0	0	0	1	0	0	0	0	0	0	0	0	0	0	0	0	0	0	0	0	0	0	0	0
0	0	20-Feb-2021	22-Feb-2021	2	1	1	12	11	43	1	6	7	7	8	8	0	0	0	0	0	1	0	1	1	1	1	1	1	1	0	0	0	0	0	0	1	0	0	0	0	0	0	0	1	0	0	1	1	0	0	0	0	0	0	0	0
0	0	21-Feb-2021	22-Feb-2021	1	1	1	16	14	47	0	6	6	7	8	8	0	0	0	0	0	1	0	0	1	1	1	1	1	1	0	0	0	0	0	0	0	1	0	0	0	0	0	0	0	0	0	0	0	0	0	0	0	0	0	0	0
0	0	22-Feb-2021	22-Feb-2021	0	1	1	11	11	68	1	6	6	6	8	8	0	1	0	0	1	1	0	1	1	1	1	1	1	0	1	0	0	0	0	0	0	1	0	0	0	0	0	0	0	0	0	0	0	0	0	0	0	0	0	0	0
0	0	22-Feb-2021	22-Feb-2021	0	0	1	23	23	69	1	6	6	6	7	8	0	0	0	0	0	1	0	1	1	1	1	1	1	0	1	0	0	0	0	0	1	0	0	0	0	0	0	0	0	0	0	0	0	0	0	0	0	0	0	0	0
1	0	21-Feb-2021	22-Feb-2021	1	0	0	4	3	48	1	6	2	2	1	1	0	0	0	0	0	1	0	1	1	1	1	1	1	0	1	0	0	0	0	0	1	0	0	0	0	0	0	0	0	0	0	0	0	0	0	0	0	0	0	0	0
1	0	20-Feb-2021	22-Feb-2021	2	0	0	6	4	36	1	6	3	1	1	1	0	0	0	0	0	1	0	1	1	1	1	1	1	0	1	0	0	0	0	0	0	1	0	0	0	0	0	0	0	0	0	0	0	0	0	0	0	0	0	0	0
1	0	19-Feb-2021	22-Feb-2021	3	0	0	9	6	71	1	6	5	2	2	1	0	1	0	0	1	1	0	1	1	1	1	1	1	0	1	0	0	0	0	0	1	0	0	0	0	0	0	0	0	0	0	0	0	0	0	0	0	0	0	0	0
1	0	19-Feb-2021	22-Feb-2021	3	0	0	13	10	46	1	5	4	3	2	1	0	0	0	0	0	1	0	1	1	1	1	1	1	0	1	0	0	0	0	0	1	0	0	0	0	0	0	0	0	0	0	0	0	0	0	0	0	0	0	0	0
1	0	15-Feb-2021	22-Feb-2021	7	0	0	21	14	65	1	6	5	5	2	1	0	0	0	0	0	1	0	0	1	1	1	1	1	0	1	0	0	0	0	0	1	0	0	0	0	0	0	0	0	0	0	0	0	0	0	0	0	0	0	0	0
1	0	22-Feb-2021	22-Feb-2021	0	1	1	6	6	75	0	5	3	8	8	8	1	1	0	0	2	1	0	1	1	1	1	1	1	0	1	0	0	0	0	0	1	0	0	0	0	0	0	0	0	0	1	1	0	0	0	0	0	0	0	0	0
0	0	7-Feb-2021	23-Feb-2021	16	1	1	2	1	52	1	5	8	8	8	8	1	1	0	0	2	1	0	1	1	1	1	1	1	1	0	0	0	0	0	0	1	0	0	0	0	0	0	0	0	0	0	0	0	0	0	0	0	0	0	0	0
0	0	12-Feb-2021	23-Feb-2021	11	0	0	7	4	36	0	5	3	2	1	1	0	0	0	0	0	1	0	1	1	1	1	1	1	0	1	0	0	0	0	0	1	0	0	0	0	0	0	0	0	0	0	0	0	0	0	0	0	0	0	0	0
0	0	14-Feb-2021	23-Feb-2021	9	1	0	3	2	32	1	6	2	2	2	1	0	0	0	0	0	1	0	1	1	1	1	1	1	1	0	0	0	0	0	0	1	0	0	0	0	0	0	0	0	0	0	0	0	0	0	0	0	0	0	0	0
0	0	17-Feb-2021	23-Feb-2021	6	1	1	11	7	48	1	5	6	8	8	8	0	0	0	0	0	1	0	1	1	1	1	1	1	0	1	0	0	0	0	0	1	0	0	0	0	0	0	0	0	0	0	0	0	0	0	0	0	0	0	0	0
0	0	20-Feb-2021	23-Feb-2021	3	0	0	14	11	36	0	5	5	5	2	2	0	0	0	0	0	1	0	1	1	1	1	1	1	0	1	0	0	0	0	0	1	0	0	0	0	0	0	0	0	0	0	0	0	0	0	0	0	0	0	0	0
0	0	20-Feb-2021	23-Feb-2021	3	0	1	17	15	49	1	6	7	7	7	8	0	0	0	0	0	1	0	1	1	1	1	1	1	2	1	0	0	0	0	0	0	1	0	0	0	0	0	0	0	0	0	0	0	0	0	0	0	0	0	0	0
0	0	21-Feb-2021	23-Feb-2021	2	1	1	5	4	54	0	6	6	8	8	8	0	1	0	0	1	1	0	1	1	1	1	1	1	0	1	0	0	0	0	0	1	0	0	0	0	0	0	0	0	0	0	0	0	0	0	0	0	0	0	0	0
0	0	21-Feb-2021	23-Feb-2021	2	0	0	12	6	67	1	4	4	2	2	2	0	0	0	0	0	1	0	1	1	1	1	1	1	1	0	0	0	0	0	0	1	0	0	0	0	0	0	0	0	0	0	0	0	0	0	0	0	0	0	0	0
0	0	21-Feb-2021	23-Feb-2021	2	0	0	3	1	32	1	5	2	2	1	1	0	0	0	0	0	1	0	1	1	1	1	1	1	1	0	0	0	0	0	0	1	0	0	0	0	0	0	0	0	0	0	0	0	0	0	0	0	0	0	0	0
0	0	21-Feb-2021	23-Feb-2021	2	1	1	12	10	61	1	6	6	7	8	8	0	0	0	0	0	1	0	1	1	1	1	1	1	1	0	0	0	0	0	0	1	0	0	0	0	0	0	0	0	0	0	0	0	0	0	0	0	0	0	0	0
0	0	22-Feb-2021	23-Feb-2021	1	1	1	7	6	53	0	6	7	8	8	8	1	1	0	0	2	1	0	1	1	1	1	1	1	0	1	0	0	0	0	0	0	1	0	0	0	0	0	0	0	0	0	0	0	0	0	0	0	0	0	0	0
1	0	21-Feb-2021	23-Feb-2021	2	0	0	7	5	59	1	5	4	2	1	1	1	1	0	0	2	1	0	1	1	1	1	1	1	0	1	0	0	0	0	0	1	0	0	0	0	0	0	0	0	0	0	1	0	0	0	0	0	0	0	0	0
1	0	21-Feb-2021	23-Feb-2021	2	0	0	5	3	54	0	5	2	1	1	1	0	0	0	0	0	1	0	1	1	1	1	1	1	0	1	0	0	0	0	0	1	0	0	0	0	0	0	0	0	0	0	0	0	0	0	0	0	0	0	0	0
1	0	21-Feb-2021	23-Feb-2021	2	0	0	5	3	59	1	6	2	1	1	1	0	0	0	0	0	1	0	1	1	1	1	1	1	0	1	0	0	0	0	0	1	0	0	0	0	0	0	0	0	0	0	1	0	0	0	0	0	0	0	0	0
1	0	21-Feb-2021	23-Feb-2021	2	0	0	5	3	57	0	6	2	1	1	1	0	0	0	0	0	1	0	1	1	1	1	1	1	0	1	0	0	0	0	0	1	0	0	0	0	0	0	0	0	0	0	0	0	0	0	0	0	0	0	0	0
1	0	20-Feb-2021	23-Feb-2021	3	0	0	11	8	33	0	5	4	2	1	1	0	0	0	0	0	1	0	0	1	1	1	1	1	0	1	0	0	0	0	0	1	0	0	0	0	0	0	0	0	0	0	0	0	0	0	0	0	0	0	0	0
1	0	22-Feb-2021	23-Feb-2021	1	0	0	5	4	66	1	5	3	1	1	1	0	1	0	0	1	1	0	0	1	1	1	1	1	0	1	0	0	0	0	0	1	0	0	0	0	0	0	0	0	0	0	0	0	0	0	0	0	0	0	0	0
1	0	22-Feb-2021	23-Feb-2021	1	0	0	6	5	60	0	6	3	1	1	1	0	0	0	0	0	1	0	0	1	1	1	1	1	0	1	0	0	0	0	0	1	0	0	0	0	0	0	0	0	0	0	0	0	0	0	0	0	0	0	0	0
1	0	21-Feb-2021	23-Feb-2021	2	0	0	7	5	61	1	6	5	2	1	1	0	1	0	0	1	1	0	0	1	1	1	1	1	0	1	0	0	0	0	0	1	0	0	0	0	0	0	0	0	0	0	0	0	0	0	0	0	0	0	0	0
0	0	19-Feb-2021	24-Feb-2021	5	0	0	9	8	45	0	6	5	5	3	2	0	0	0	0	0	1	0	1	1	1	1	1	0	0	0	0	0	0	0	0	1	0	0	0	0	0	0	0	0	0	0	0	0	0	0	0	0	0	0	0	0
0	0	22-Feb-2021	24-Feb-2021	2	1	1	8	6	60	1	6	7	8	8	8	0	0	0	0	0	1	0	1	1	1	1	1	1	0	1	0	0	0	0	0	1	0	0	0	0	0	0	0	0	0	0	0	0	0	0	0	0	0	0	0	0
0	0	23-Feb-2021	24-Feb-2021	1	1	1	3	2	67	0	6	8	8	8	8	0	1	0	0	1	1	0	1	1	1	1	1	1	1	0	0	0	0	0	0	1	0	0	0	0	0	0	0	0	0	0	0	0	0	0	0	0	0	0	0	0
0	0	23-Feb-2021	24-Feb-2021	1	1	1	6	5	52	1	6	7	8	8	8	0	0	0	0	0	1	0	1	1	1	1	1	1	0	1	0	0	0	0	0	1	0	0	0	0	0	0	0	0	0	0	0	0	0	0	0	0	0	0	0	0
0	0	23-Feb-2021	24-Feb-2021	1	1	1	8	7	67	1	6	7	8	8	8	0	0	0	0	0	1	0	1	1	1	1	1	1	1	0	0	0	0	0	0	1	0	0	0	0	0	0	0	0	0	0	0	0	0	0	0	0	0	0	0	0
1	0	23-Feb-2021	24-Feb-2021	1	0	0	6	5	57	0	6	3	1	1	1	0	0	0	0	0	1	0	1	1	1	1	1	1	1	0	0	0	0	0	0	1	0	0	0	0	0	0	0	0	0	0	0	0	0	0	0	0	0	0	0	0
1	0	22-Feb-2021	24-Feb-2021	2	0	0	4	2	39	0	6	1	1	1	1	0	0	0	0	0	1	0	1	1	1	1	1	1	1	0	0	0	0	0	0	1	0	0	0	0	0	0	0	0	0	0	0	0	0	0	0	0	0	0	0	0
1	0	22-Feb-2021	24-Feb-2021	2	0	0	3	1	31	1	6	2	1	1	1	0	0	0	0	0	1	0	1	1	1	1	1	1	0	1	0	0	0	0	0	1	0	0	0	0	0	0	0	0	0	0	0	0	0	0	0	0	0	0	0	0
1	0	22-Feb-2021	24-Feb-2021	2	0	0	14	12	71	1	6	6	5	5	4	0	1	0	0	1	1	0	1	1	1	1	1	1	0	1	0	0	0	0	0	1	0	0	0	0	0	0	0	0	0	0	0	0	0	0	0	0	0	0	0	0
1	0	23-Feb-2021	24-Feb-2021	1	0	0	3	2	60	1	6	1	1	1	1	0	1	0	0	0	1	0	0	1	1	1	1	1	0	1	0	0	0	0	0	1	0	0	0	0	0	0	0	0	0	0	0	0	0	0	0	0	0	0	0	0
1	0	20-Feb-2021	24-Feb-2021	4	0	0	9	5	51	0	6	4	1	1	1	0	0	0	0	0	1	0	0	1	1	1	1	1	0	1	0	0	0	0	0	1	0	0	0	0	0	0	0	0	0	0	0	0	0	0	0	0	0	0	0	0
1	0	18-Feb-2021	24-Feb-2021	6	1	1	6	0	58	0	6	8	8	8	8	0	0	0	0	0	1	0	1	1	1	1	1	0	0	0	0	0	0	0	0	1	0	0	0	0	0	0	0	0	0	0	0	0	0	0	0	0	0	0	0	0
0	0	20-Feb-2021	25-Feb-2021	5	1	1	19	14	33	1	5	6	6	8	8	0	0	0	0	0	1	0	1	1	1	1	1	1	1	0	0	0	0	0	0	1	0	0	0	0	0	0	0	0	0	0	0	0	0	0	0	0	0	0	0	0
0	0	22-Feb-2021	25-Feb-2021	1	1	1	15	12	60	1	6	6	7	8	8	0	0	0	0	0	1	0	1	1	1	1	1	1	1	0	0	0	0	0	0	1	0	0	0	0	0	0	0	0	0	0	0	0	0	0	0	0	0	0	0	0
0	0	24-Feb-2021	25-Feb-2021	1	1	1	8	7	50	0	6	7	8	8	8	0	0	0	0	0	1	0	1	1	1	1	1	1	1	0	0	0	0	0	0	1	0	0	0	0	0	0	0	0	0	0	0	0	0	0	0	0	0	0	0	0
0	0	24-Feb-2021	25-Feb-2021	1	1	1	8	6	47	0	6	7	8	8	8	0	0	0	0	0	1	0	1	1	1	1	1	1	1	0	0	0	0	0	0	1	0	0	0	0	0	0	0	0	0	0	0	0	0	0	0	0	0	0	0	0
0	0	24-Feb-2021	25-Feb-2021	1	1	1	5	3	51	1	5	7	8	8	8	0	0	0	0	0	1	0	1	1	1	1	1	1	1	0	0	0	0	0	0	1	0	0	0	0	0	0	0	0	0	0	0	0	0	0	0	0	0	0	0	0
0	0	24-Feb-2021	25-Feb-2021	1	1	1	12	11	56	1	5	6	7	8	8	0	0	0	0	0	1	0	1	1	1	1	1	1	1	0	0	0	0	0	0	1	0	0	0	0	0	0	0	0	0	0	0	0	0	0	0	0	0	0	0	0
1	0	22-Feb-2021	25-Feb-2021	3	0	0	7	4	36	0	5	3	1	1	1	0	0	0	0	0	1	0	1	1	1	1	1	1	0	1	0	0	0	0	0	1	0	0	0	0	0	0	0	0	0	0	0	0	0	0	0	0	0	0	0	0
1	0	24-Feb-2021	25-Feb-2021	0	0	0	7	6	53	1	6	3	1	1	1	0	1	0	0	1	1	0	1	1	1	1	1	1	1	0	0	0	0	0	0	1	0	0	0	0	0	0	0	0	0	0	0	0	0	0	0	0	0	0	0	0
1	0	23-Feb-2021	25-Feb-2021	5	0	0	22	20	36	1	5	1	1	1	1	0	0	0	0	0	1	0	1	1	1	1	1	1	1	0	0	0	0	0	0	1	0	0	0	0	0	0	0	0	1	0	1	0	0	0	0	0	0	0	0	0
1	0	21-Feb-2021	25-Feb-2021	4	0	0	6	2	42	0	6	2	1	1	1	0	0	0	0	0	1	0	1	1	1	1	1	1	1	0	0	0	0	0	0	1	0	0	0	0	0	0	0	0	0	0	0	0	0	0	0	0	0	0	0	0
1	0	21-Feb-2021	25-Feb-2021	4	0	0	7	3	42	0	6	2	1	1	1	0	0	0	0	0	1	0	1	1	1	1	1	1	1	0	0	0	0	0	0	1	0	0	0	0	0	0	0	0	0	0	0	0	0	0	0	0	0	0	0	0
1	0	24-Feb-2021	25-Feb-2021	3	0	1	18	15	62	1	6	7	7	7	8	0	0	0	0	0	1	0	1	1	1	1	1	1	1	0	0	0	0	0	0	1	0	0	0	0	0	0	0	0	0	0	1	0	0	0	0	0	0	0	0	0
1	0	22-Feb-2021	25-Feb-2021	3	0	0	11	8	65	1	6	3	2	1	1	1	1	1	0	3	1	0	1	1	1	1	1	1	0	0	0	0	0	0	0	1	0	0	0	0	0	0	0	0	0	0	0	0	0	0	0	0	0	0	0	0
1	0	22-Feb-2021	25-Feb-2021	3	0	0	13	10	48	0	6	4	3	2	1	0	0	0	0	0	1	0	1	1	1	1	1	1	0	1	0	0	0	0	0	1	0	0	0	0	0	0	0	0	0	0	0	0	0	0	0	0	0	0	0	0
0	0	16-Feb-2021	26-Feb-2021	10	0	0	11	10	27	1	5	5	5	2	1	1	1	0	0	2	1	0	1	1	1	1	1	1	0	1	0	0	0	0	0	0	1	0	0	0	0	0	0	0	0	0	0	0	0	0	0	0	0	0	0	0
0	0	24-Feb-2021	26-Feb-2021	2	1	1	12	11	71	0	6	6	7	8	8	0	0	0	0	0	1	0	1	1	1	1	1	1	1	0	0	0	0	0	0	1	0	0	0	0	0	0	0	0	0	0	0	0	0	0	0	0	0	0	0	0
0	0	24-Feb-2021	26-Feb-2021	2	0	0	5	4	36	1	6	5	2	2	2	0	0	0	0	0	1	0	1	1	1	1	1	1	1	0	0	0	0	0	0	1	0	0	0	0	0	0	0	0	0	0	0	0	0	0	0	0	0	0	0	0
0	0	25-Feb-2021	26-Feb-2021	1	0	0	5	4	56	0	6	4	2	1	1	0	0	0	0	0	1	0	1	1	1	1	1	1	1	0	0	0	0	0	0	1	0	0	0	0	0	0	0	0	0	0	0	0	0	0	0	0	0	0	0	0
0	0	25-Feb-2021	26-Feb-2021	1	1	1	3	2	80	0	6	8	8	8	8	0	0	0	0	0	1	0	1	1	1	1	1	1	1	0	0	0	0	0	0	1	0	0	0	0	0	0	0	0	0	0	0	0	0	0	0	0	0	0	0	0
0	0	25-Feb-2021	26-Feb-2021	1	0	0	7	6	37	1	6	4	2	2	1	0	0	0	0	0	1	0	1	1	1	1	1	1	1	0	0	0	0	0	0	1	0	0	0	0	0	0	0	0	0	0	0	0	0	0	0	0	0	0	0	0
1	0	22-Feb-2021	26-Feb-2021	4	0	0	5	1	74	0	5	1	1	1	1	0	0	0	0	0	1	0	1	1	1	1	1	1	0	1	0	0	0	0	0	1	0	0	0	0	0	0	0	0	0	0	0	0	0	0	0	0	0	0	0	0
1	0	20-Feb-2021	26-Feb-2021	6	0	0	15	9	77	0	6	5	4	2	1	1	1	0	0	2	1	0	1	1	1	1	1	1	0	1	0	0	0	0	0	0	1	0	0	0	0	0	0	0	0	0	0	0	0	0	0	0	0	0	0	0
1	0	25-Feb-2021	26-Feb-2021	1	0	0	11	10	78	0	6	4	3	1	1	0	0	0	0	0	1	0	1	1	1	1	1	1	0	1	0	0	0	0	0	1	0	0	0	0	0	0	0	0	0	0	1	0	0	0	0	0	0	0	0	0
1	0	25-Feb-2021	26-Feb-2021	1	0	0	9	8	57	1	6	4	3	1	1	0	1	1	0	2	1	0	1	1	1	1	1	1	1	0	0	0	0	0	0	1	0	0	0	0	0	0	0	0	0	0	1	0	0	0	0	0	0	0	0	1
0	0	21-Feb-2021	27-Feb-2021	6	0	0	15	13	75	0	6	6	6	2	2	0	0	0	0	0	1	0	1	1	1	1	1	1	0	1	0	0	0	0	0	1	0	0	0	0	0	0	0	0	0	0	0	0	0	0	0	0	0	0	0	0
0	0	25-Feb-2021	27-Feb-2021	2	1	1	11	9	72	1	6	6	7	8	8	0	0	0	0	0	1	0	1	1	1	1	1	1	1	0	0	0	0	0	0	1	0	0	0	0	0	0	0	0	1	0	0	0	0	0	0	0	0	0	0	0
0	0	26-Feb-2021	27-Feb-2021	1	0	0	11	10	31	0	6	4	3	2	2	0	1	0	0	1	1	0	1	1	1	1	1	1	1	0	0	0	0	0	0	1	0	0	0	0	0	0	0	0	0	0	0	0	0	0	0	0	0	0	0	0
0	0	26-Feb-2021	27-Feb-2021	1	1	1	6	5	53	1	6	7	8	8	8	0	0	0	0	0	1	0	1	1	1	1	1	1	1	0	0	0	0	0	0	1	0	0	0	0	0	0	0	0	0	0	0	0	0	0	0	0	0	0	0	0
0	0	23-Feb-2021	28-Feb-2021	5	0	0	20	15	64	0	6	5	5	4	1	1	1	0	0	2	1	0	1	1	1	1	1	1	0	1	0	0	0	0	0	1	0	0	0	0	0	0	0	0	0	0	0	0	0	0	0	0	0	0	0	0
0	0	25-Feb-2021	28-Feb-2021	3	1	1	11	8	66	0	6	7	7	8	8	0	0	0	0	0	1	0	1	1	1	1	1	1	1	0	0	0	0	0	0	1	0	0	0	0	0	0	0	0	0	0	0	0	0	0	0	0	0	0	0	0
0	0	27-Feb-2021	28-Feb-2021	1	0	0	4	3	29	0	5	2	2	2	2	0	0	0	0	0	1	0	1	1	1	1	1	1	1	0	0	0	0	0	0	1	0	0	0	0	0	0	0	0	0	0	0	0	0	0	0	0	0	0	0	0
0	0	28-Feb-2021	28-Feb-2021	0	0	0	4	4	38	1	6	4	2	2	1	0	0	0	0	0	1	0	1	1	1	1	1	1	1	0	0	0	0	0	0	1	0	0	0	0	0	0	0	0	0	0	0	0	0	0	0	0	0	0	0	0
1	0	27-Feb-2021	1-Mar-2021	2	0	0	5	3	32	0	6	1	1	1	1	0	0	0	0	0	1	0	1	1	1	1	1	1	1	0	0	0	0	0	0	1	0	0	0	0	0	0	0	0	0	0	0	0	0	0	0	0	0	0	0	0
1	0	26-Feb-2021	1-Mar-2021	3	0	0	6	3	46	0	6	2	1	1	1	0	1	0	0	1	1	0	1	1	1	1	1	1	1	0	0	0	0	0	0	1	0	0	0	0	0	0	0	0	0	0	0	0	0	0	0	0	0	0	0	0
1	0	27-Feb-2021	1-Mar-2021	2	0	0	4	2	32	1	5	1	1	1	1	0	0	1	0	1	1	0	1	1	1	1	1	1	1	0	0	0	0	0	0	1	0	0	0	0	0	0	0	0	0	0	0	0	0	0	0	0	0	0	0	0
1	0	27-Feb-2021	1-Mar-2021	2	0	0	23	21	61	1	6	3	1	1	1	0	0	0	0	0	1	0	1	1	1	1	1	1	1	0	0	0	0	0	0	1	0	0	0	0	0	0	0	0	0	0	1	0	0	0	0	1	0	0	0	1
1	0	27-Feb-2021	1-Mar-2021	2	0	0	10	8	38	0	6	4	3	1	1	0	0	0	0	0	1	0	1	1	1	1	1	1	0	1	0	0	0	0	0	1	0	0	0	0	0	0	0	0	0	0	0	0	0	0	0	0	0	0	0	0
1	0	27-Feb-2021	1-Mar-2021	2	0	1	18	16	65	1	6	6	5	5	8	0	0	0	0	0	1	0	1	1	1	1	1	1	0	1	0	0	0	0	0	1	0	0	0	0	0	0	0	0	0	0	1	0	0	0	0	0	0	0	0	0
0	0	1-Mar-2021	2-Mar-2021	1	1	1	9	8	40	1	6	7	7	8	8	0	0	0	0	0	1	0	1	1	1	1	1	1	1	0	0	0	0	0	0	1	0	0	0	0	0	0	0	0	0	0	0	0	0	0	0	0	0	0	0	0
1	0	28-Feb-2021	2-Mar-2021	2	1	1	9	7	40	0	6	7	8	8	8	0	0	0	0	0	1	0	1	1	1	1	1	1	1	0	0	0	0	0	0	1	0	0	0	0	0	0	0	0	0	0	0	0	0	0	0	0	0	0	0	0
1	0	24-Feb-2021	2-Mar-2021	6	0	0	14	8	63	0	6	5	4	2	2	0	0	0	0	0	1	0	1	1	1	1	1	1	0	1	0	0	0	0	0	1	0	0	0	0	0	0	0	0	0	0	0	0	0	0	0	0	0	0	0	0
1	0	27-Feb-2021	2-Mar-2021	3	0	0	11	8	59	1	5	5	5	2	1	0	1	0	0	1	1	0	1	1	1	1	1	1	0	1	0	0	0	0	0	1	0	0	0	0	0	0	0	0	0	0	1	0	0	0	0	0	0	0	0	0
0	1	03/03/21	03/03/21	0	1	1	14	14	55	1	6	6	7	8	8	0	0	0	0	0	1	0	0	0	0	0	1	0	0	0	1	0	0	0	0	0	1	0	0	0	0	0	0	1	0	0	0	0	0	0	0	0	0	0	1	0
0	1	23/02/21	03/03/21	8	0	0	20	12	54	0	5	2	2	2	2	0	1	0	0	1	1	0	1	0	0	0	1	0	1	0	0	0	1	0	0	1	0	0	1	1	1	1	1	0	0	0	0	0	0	0	0	0	0	0	0	0
0	1	01/03/21	03/03/21	2	0	0	14	12	55	1	6	6	6	2	2	1	0	0	0	1	1	0	1	0	0	0	1	0	1	0	0	0	1	0	0	1	0	0	1	1	1	1	1	0	0	0	0	0	0	0	0	0	0	0	1	0
1	1	26/02/21	03/03/21	5	0	0	11	6	40	1	5	4	1	1	1	0	0	0	0	0	1	0	1	0	0	0	1	0	0	0	1	0	0	0	0	1	0	0	1	1	0	0	0	0	0	0	1	0	0	0	0	0	0	0	0	0
1	1	27/02/21	03/03/21	4	0	0	10	6	55	1	6	3	1	1	1	0	0	0	0	0	1	0	0	0	0	0	1	0	0	0	1	0	0	0	0	0	1	0	0	0	0	0	0	0	0	0	0	0	0	0	0	0	0	0	0	0
1	1	24/02/21	03/03/21	7	0	0	12	5	45	1	5	3	2	1	1	0	0	1	0	1	1	0	0	0	0	0	1	0	0	0	1	0	0	0	0	0	1	0	1	0	0	0	0	0	0	0	0	0	0	0	0	1	0	0	0	0
1	1	01/03/21	03/03/21	2	0	0	10	8	54	1	5	4	3	2	1	0	0	0	0	0	0	1	0	0	0	0	1	0	0	0	1	0	0	0	0	0	1	0	1	0	0	0	0	0	0	0	1	1	0	0	0	0	0	0	0	0
1	1	03/03/21	03/03/21	0	0	0	19	19	48	1	6	5	5	3	1	0	1	0	0	1	1	0	0	0	0	0	1	0	0	0	1	0	0	0	0	0	1	0	1	0	0	0	0	0	0	0	0	0	0	0	0	0	0	0	0	0
1	1	27/02/21	03/03/21	4	0	0	10	6	57	1	6	6	2	1	1	1	1	0	0	3	1	0	1	0	0	0	1	0	1	0	0	0	1	0	0	1	0	0	1	1	1	1	1	0	0	0	0	0	0	0	0	0	0	0	0	0
1	1	28/02/21	03/03/21	3	0	0	15	12	60	1	6	6	6	2	2	1	1	0	0	3	1	0	1	0	0	0	1	0	1	0	0	0	1	0	0	1	0	0	1	1	1	1	1	0	0	0	0	0	0	0	0	0	0	0	1	0
1	1	02/03/21	03/03/21	1	0	0	4	3	62	1	6	2	1	1	1	0	0	0	0	0	1	0	1	0	0	0	1	0	1	0	0	0	1	0	0	1	0	0	1	1	1	1	1	0	0	0	1	0	0	0	0	0	0	0	0	0
1	1	01/03/21	03/03/21	2	0	0	6	4	75	0	5	4	2	2	1	0	0	0	0	0	1	0	1	0	0	0	1	0	1	0	0	0	1	0	0	1	0	0	1	1	1	1	1	0	0	0	0	0	0	0	0	0	0	0	0	0
1	1	01/03/21	03/03/21	2	0	0	8	6	69	0	6	5	2	2	1	1	0	0	0	1	1	0	1	0	0	0	1	0	1	0	0	0	1	0	0	1	0	0	1	1	1	1	1	0	0	0	0	0	0	0	0	0	0	0	0	0
1	1	02/03/21	03/03/21	1	0	0	2	1	48	1	6	2	1	2	1	0	0	0	0	0	1	0	1	0	0	0	1	0	1	0	0	0	1	0	0	1	0	0	1	1	1	1	1	0	0	0	0	0	0	0	0	0	0	0	0	0
1	1	02/03/21	03/03/21	1	0	0	7	6	61	1	6	2	2	1	2	0	0	0	0	0	1	0	1	0	0	0	1	0	1	0	0	0	1	0	0	1	0	0	1	1	1	1	1	0	0	0	0	0	0	0	0	0	0	0	0	0
1	1	01/03/21	03/03/21	2	1	1	3	1	77	1	6	8	8	8	8	0	0	0	0	0	1	0	1	0	0	0	1	0	1	0	0	0	1	0	0	1	0	0	1	1	1	1	1	0	0	0	0	0	0	0	0	0	0	0	1	0
1	1	02/03/21	03/03/21	1	0	0	3	2	38	0	5	2	2	1	1	0	0	0	0	0	1	0	1	0	0	0	1	0	1	0	0	0	1	0	0	1	0	0	1	1	1	1	1	0	0	0	1	0	0	0	0	0	0	0	0	0
1	1	02/03/21	03/03/21	1	0	0	2	1	74	0	5	2	1	1	1	0	0	0	0	0	1	0	1	0	0	0	1	0	1	0	0	0	1	0	0	1	0	0	1	1	1	1	1	0	0	0	0	0	0	0	0	0	0	0	0	0
1	1	02/03/21	03/03/21	1	0	0	2	1	56	0	5	2	1	1	1	1	1	0	0	3	1	0	1	0	0	0	1	0	1	0	0	0	1	0	0	1	0	0	1	1	1	1	1	0	0	0	1	0	0	0	0	0	0	0	0	0
0	1	01/03/21	04/03/21	3	0	0	14	11	39	1	5	5	2	2	1	0	0	0	0	0	1	0	1	0	0	0	1	0	1	0	0	0	1	0	0	1	0	0	1	1	1	1	1	0	0	0	0	0	0	0	0	0	0	0	0	0
0	0	22-Feb-2021	4-Mar-2021	10	1	1	21	11	72	1	6	6	6	8	8	0	0	0	0	0	1	0	1	1	1	1	1	1	1	0	0	0	0	0	0	1	0	0	0	0	0	0	0	0	0	0	0	0	0	0	0	0	0	0	0	0
0	0	3-Mar-2021	4-Mar-2021	1	0	0	9	8	51	0	6	4	4	2	2	0	0	0	0	0	1	0	1	1	1	1	1	1	1	0	0	0	0	0	0	1	0	0	0	0	0	0	0	0	0	0	0	0	0	0	0	0	0	0	0	0
0	0	3-Mar-2021	4-Mar-2021	1	0	0	7	6	41	1	6	4	2	1	1	0	0	1	0	1	1	0	1	1	1	1	1	1	1	0	0	0	0	0	0	1	0	0	0	0	0	0	0	0	0	0	0	0	0	0	0	0	0	0	0	0
1	1	03/03/21	04/03/21	1	0	0	3	2	46	1	5	2	1	1	1	0	1	0	0	1	0	0	0	0	0	0	1	0	0	0	1	0	0	0	0	1	0	0	1	0	1	0	0	0	0	0	0	0	0	0	0	0	0	0	0	0
1	1	04/03/21	04/03/21	0	0	0	8	8	41	1	6	4	2	1	1	0	0	0	0	0	1	0	0	0	0	0	1	0	0	0	0	0	0	0	0	0	1	0	0	0	0	0	0	0	0	0	0	0	0	0	0	0	0	0	0	0
1	1	25/02/21	04/03/21	7	0	0	57	50	73	0	6	6	6	6	5	1	1	0	0	2	1	1	0	0	0	0	1	0	0	0	1	1	0	0	0	1	0	0	0	0	0	0	0	0	0	0	0	0	0	0	0	0	0	0	0	0
1	1	03/03/21	04/03/21	1	0	0	6	5	56	1	5	5	2	2	1	0	1	0	0	1	1	0	1	0	0	0	1	0	1	0	0	0	1	0	0	1	0	0	1	1	1	1	1	0	0	0	0	0	0	0	0	0	0	0	0	1
1	1	01/03/21	04/03/21	3	0	1	17	14	83	0	6	7	7	7	8	0	0	0	0	0	1	0	1	0	0	0	1	0	1	0	0	0	1	0	0	1	0	0	1	1	1	1	1	0	0	0	0	0	0	0	0	0	0	0	1	0
1	0	2-Mar-2021	4-Mar-2021	2	1	1	8	6	56	0	6	7	8	8	8	0	1	0	0	1	1	0	1	1	1	1	1	1	0	1	0	0	0	0	0	1	0	0	0	0	0	0	0	0	0	0	0	0	0	0	0	0	0	0	0	0
1	0	2-Mar-2021	4-Mar-2021	2	0	0	7	5	43	1	6	5	2	1	1	0	0	0	0	0	1	0	1	1	1	1	1	1	0	1	0	0	0	0	0	1	0	0	0	0	0	0	0	0	0	0	0	0	0	0	0	0	0	0	0	0
0	1	02/03/21	05/03/21	3	0	1	31	28	67	1	6	6	7	7	8	0	0	0	0	0	1	0	0	0	0	0	1	0	0	0	1	0	0	0	0	0	1	0	1	0	0	0	0	0	0	0	0	0	0	0	0	0	0	0	1	0
0	1	01/03/21	05/03/21	4	0	0	7	3	30	1	5	3	2	2	1	0	1	0	0	1	1	0	1	0	0	0	1	0	0	0	0	1	0	0	0	0	1	0	1	0	0	0	0	0	0	0	0	0	0	0	0	0	0	0	0	0
0	1	04/03/21	05/03/21	1	0	0	18	17	47	1	6	5	3	7	7	0	0	0	0	0	0	0	0	0	0	0	1	0	0	0	1	0	0	0	0	0	1	0	1	0	0	0	0	0	0	0	1	1	0	0	0	0	0	0	1	0
0	1	05/03/21	05/03/21	0	0	0	15	15	74	0	4	4	2	1	1	0	0	0	0	1	1	0	0	0	0	0	1	0	0	0	1	0	0	0	0	0	1	0	0	0	0	0	0	0	0	0	0	0	0	0	0	0	0	0	1	0
0	1	05/03/21	05/03/21	0	0	0	14	14	58	1	5	4	1	1	1	0	0	1	0	1	1	0	1	0	0	0	1	1	0	0	0	0	0	0	0	0	1	0	0	0	0	0	0	0	1	0	0	0	0	0	0	0	0	0	0	0
1	1	24/02/21	05/03/21	9	0	0	23	14	54	1	6	4	2	1	1	1	1	0	0	2	1	0	0	0	0	0	1	0	0	0	0	0	0	0	1	0	1	0	0	0	0	0	0	0	0	0	0	0	0	0	0	0	0	0	0	0
1	1	02/03/21	05/03/21	3	0	0	27	24	75	0	5	5	5	4	1	0	1	0	0	2	1	0	0	0	0	0	1	0	0	0	0	0	0	1	1	0	1	0	0	0	0	0	0	0	0	0	0	0	0	0	0	0	0	0	0	0
1	1	25/02/21	05/03/21	8	0	0	20	12	56	1	6	4	4	1	1	0	0	0	0	0	0	1	0	0	0	0	1	0	0	0	1	0	0	0	0	0	1	0	1	0	0	0	0	0	0	0	1	0	0	0	0	0	0	0	0	0
1	1	27/02/21	05/03/21	6	0	0	13	7	53	1	6	3	1	1	1	0	1	1	0	2	1	0	1	0	0	0	1	0	0	0	1	0	0	0	0	1	0	0	1	0	0	0	0	0	0	0	0	0	0	0	0	0	0	0	0	0
1	1	01/03/21	05/03/21	4	0	0	6	2	48	1	5	2	1	1	1	0	0	0	0	0	1	0	0	0	0	0	1	1	0	0	0	0	0	0	0	0	1	0	0	0	0	0	0	0	0	0	0	0	0	0	0	0	0	0	0	0
1	1	04/03/21	05/03/21	1	0	0	12	11	51	1	6	4	3	1	1	0	0	0	0	0	0	1	0	0	0	0	1	0	0	0	1	0	0	0	0	0	1	0	0	0	0	0	0	0	0	0	1	0	0	0	0	0	0	0	0	0
1	1	04/03/21	05/03/21	1	0	0	6	5	70	1	5	4	2	1	1	0	0	0	0	0	1	0	1	0	0	0	1	0	0	0	1	0	0	0	0	0	1	0	0	0	0	0	0	0	0	0	0	0	0	0	0	0	0	0	0	0
1	1	05/03/21	05/03/21	0	0	0	8	8	38	0	6	5	2	2	1	0	0	0	0	0	1	0	0	0	0	0	1	0	0	0	1	0	0	0	0	0	1	0	0	0	0	0	0	0	0	0	1	0	0	0	0	0	0	0	0	0
1	1	03/03/21	05/03/21	2	0	0	4	2	35	1	5	2	1	1	1	0	0	0	0	0	0	0	0	0	0	0	1	0	0	0	1	0	0	0	0	0	1	0	1	0	0	0	0	0	0	0	0	0	0	0	0	0	0	0	0	0
1	1	03/03/21	05/03/21	2	0	0	8	6	59	1	5	3	2	1	1	0	0	0	0	0	0	1	0	0	0	0	1	1	0	0	0	0	0	0	0	0	1	0	0	0	0	0	0	0	0	0	0	0	0	0	0	0	0	0	0	0
1	1	28/02/21	05/03/21	5	0	0	8	3	50	0	6	2	1	1	1	0	0	0	0	0	1	0	1	0	0	0	1	0	0	0	0	0	1	0	0	0	1	0	1	0	0	0	0	0	0	0	0	0	0	0	0	0	0	0	0	0
1	1	05/03/21	05/03/21	0	0	0	2	2	58	0	6	6	6	4	3	0	1	0	0	1	1	0	1	0	0	0	1	0	0	0	0	0	1	0	0	0	1	0	1	0	0	0	0	0	0	0	1	0	0	0	0	0	0	0	0	0
1	1	04/03/21	05/03/21	1	0	0	1	0	57	0	6	2	1	1	1	0	0	0	0	0	1	0	1	0	0	0	1	0	1	0	0	0	1	0	0	1	0	0	1	1	1	1	1	0	0	0	0	0	0	0	0	0	0	0	0	0
1	1	04/03/21	05/03/21	1	0	0	5	4	55	0	6	2	1	1	1	0	0	0	0	0	1	0	1	0	0	0	1	0	1	0	0	0	1	0	0	1	0	0	1	1	1	1	1	0	0	0	0	0	0	0	0	0	0	0	0	0
1	1	04/03/21	05/03/21	1	0	0	4	3	55	1	6	2	2	1	1	0	0	0	0	0	1	0	1	0	0	0	1	0	1	0	0	0	1	0	0	1	0	0	1	1	1	1	1	0	1	0	1	0	0	0	0	0	0	0	1	0
1	0	3-Mar-2021	5-Mar-2021	2	0	0	5	3	59	0	5	2	1	1	1	0	0	0	0	0	1	0	1	1	1	1	1	1	0	1	0	0	0	0	0	1	0	0	0	0	0	0	0	0	0	0	0	0	0	0	0	0	0	0	0	0
1	0	3-Mar-2021	5-Mar-2021	2	0	0	7	5	43	0	5	3	1	1	1	0	0	0	0	0	1	0	1	1	1	1	1	1	1	0	0	0	0	0	0	1	0	0	0	0	0	0	0	0	0	0	0	0	0	0	0	0	0	0	0	0
0	1	06/03/21	06/03/21	0	1	1	14	14	48	0	5	6	7	8	8	0	0	0	0	0	1	0	0	0	0	0	1	0	0	0	1	0	0	0	0	0	1	0	0	0	0	0	0	0	0	0	0	0	0	0	0	0	0	0	1	0
0	1	04/03/21	06/03/21	2	1	1	21	19	65	1	6	6	6	8	8	0	0	0	0	0	1	0	1	0	0	0	1	0	1	0	0	0	1	0	0	1	0	0	1	1	1	1	1	0	0	0	0	0	0	0	0	0	0	0	1	0
0	0	6-Mar-2021	6-Mar-2021	2	1	1	7	7	60	1	6	6	7	8	8	0	1	0	1	2	1	0	1	1	1	1	1	1	1	0	0	0	0	0	0	1	0	0	0	0	0	0	0	0	0	0	0	0	0	0	0	0	0	0	0	0
1	1	06/03/21	06/03/21	0	0	0	22	22	52	1	6	7	7	6	2	0	0	0	0	0	1	0	0	0	0	0	1	0	0	0	0	1	0	0	0	0	1	0	1	0	0	0	0	0	0	0	1	0	0	0	0	0	0	0	0	0
1	1	06/03/21	06/03/21	0	0	0	5	5	56	1	6	4	2	1	1	0	0	0	0	0	1	0	0	0	0	0	1	0	0	0	1	0	0	0	0	0	1	0	1	0	0	0	0	0	0	0	1	0	0	0	0	1	0	0	0	1
1	1	06/03/21	06/03/21	0	1	1	12	12	74	1	6	6	7	8	8	0	0	0	0	0	1	0	0	0	0	0	1	0	0	0	1	0	0	0	0	0	1	0	0	0	0	0	0	0	0	0	1	1	0	1	0	0	0	0	1	0
1	1	05/03/21	06/03/21	1	0	0	10	9	58	1	5	5	5	2	1	0	0	0	0	0	1	0	1	0	0	0	1	0	1	0	0	0	1	0	0	1	0	0	1	1	1	1	1	0	0	0	0	0	0	0	0	0	0	0	0	0
1	1	04/03/21	06/03/21	2	0	0	6	4	40	1	5	2	2	2	1	0	0	0	0	0	1	0	1	0	0	0	1	0	1	0	0	0	1	0	0	1	0	0	1	1	1	1	1	0	0	0	0	0	0	0	0	0	0	0	0	0
1	0	27-Feb-2021	6-Mar-2021	4	0	0	16	12	36	0	6	4	2	1	1	0	0	0	0	0	1	0	1	1	1	1	1	1	1	0	0	0	0	0	0	1	0	0	0	0	0	0	0	0	0	0	1	0	0	0	0	0	0	0	0	0
0	1	05/03/21	07/03/21	2	0	0	4	2	41	1	5	2	2	1	1	0	1	0	0	2	1	0	1	0	0	0	1	0	1	0	0	0	1	0	0	1	0	0	1	1	1	1	1	0	0	0	0	0	0	0	0	0	0	0	0	0
0	1	07/03/21	07/03/21	0	1	1	11	11	66	0	6	6	7	8	8	0	1	1	0	3	1	0	1	0	0	0	1	0	1	0	0	0	1	0	0	1	0	0	1	1	1	1	1	0	0	0	0	0	0	0	0	0	0	0	1	0
1	1	07/03/21	07/03/21	0	0	1	19	19	51	1	6	7	7	7	8	0	0	0	0	0	1	0	0	0	0	0	1	0	0	0	1	0	0	0	0	0	1	0	1	0	0	0	0	0	0	0	0	0	0	0	0	0	0	0	1	0
1	1	07/03/21	07/03/21	0	0	0	8	8	48	1	5	4	2	1	1	0	0	0	0	0	1	0	0	0	0	0	1	0	0	0	1	0	0	0	0	0	1	0	0	0	0	0	0	0	0	0	0	0	0	0	0	0	0	0	0	0
1	1	07/03/21	07/03/21	0	0	0	5	5	53	0	5	3	2	1	1	0	0	0	0	0	1	0	1	0	0	0	1	0	1	0	0	0	1	0	0	1	0	0	1	1	1	1	1	0	0	0	0	0	0	0	0	0	0	0	0	0
1	1	06/03/21	07/03/21	1	0	0	6	5	30	1	6	4	2	1	1	0	0	0	0	2	1	0	1	0	0	0	1	0	1	0	0	0	1	0	0	1	0	0	1	1	1	1	1	0	0	0	1	0	0	0	0	0	0	0	0	0
1	1	02/03/21	07/03/21	5	0	0	8	3	67	1	5	2	2	2	2	0	1	0	0	1	1	0	1	0	0	0	1	0	1	0	0	0	1	0	0	1	0	0	1	1	1	1	1	0	0	0	0	0	0	0	0	0	0	0	0	0
1	1	07/03/21	07/03/21	0	0	0	9	9	47	1	6	5	4	2	2	0	0	0	0	0	1	0	1	0	0	0	1	0	1	0	0	0	1	0	0	1	0	0	1	1	1	1	1	0	0	0	0	0	0	0	0	0	0	0	0	0
1	1	04/03/21	07/03/21	3	0	0	5	2	73	1	5	2	1	1	1	0	0	0	0	0	1	0	1	0	0	0	1	0	1	0	0	0	1	0	0	1	0	0	1	1	1	1	1	0	0	0	1	0	0	0	0	0	0	0	0	0
1	1	03/03/21	07/03/21	4	0	0	8	4	33	0	5	1	1	1	1	0	0	0	0	0	1	0	1	0	0	0	1	0	1	0	0	0	1	0	0	1	0	0	1	1	1	1	1	0	1	0	0	0	0	0	0	0	0	0	0	0
1	1	04/03/21	07/03/21	3	0	0	7	4	86	1	5	2	2	1	1	0	0	0	0	0	1	0	1	0	0	0	1	0	1	0	0	0	1	0	0	1	0	0	1	1	1	1	1	0	0	0	0	0	0	0	0	0	0	0	0	0
0	1	08/03/21	08/03/21	0	0	0	31	31	34	1	5	6	6	5	4	0	0	0	0	0	1	0	0	0	0	0	1	0	0	0	0	0	0	1	1	0	1	0	0	0	0	0	0	0	0	0	0	0	0	0	0	0	0	0	0	0
0	1	08/03/21	08/03/21	0	0	0	9	9	54	1	5	5	4	2	2	0	1	0	0	1	1	0	0	0	0	0	1	1	0	0	0	0	0	0	0	0	1	0	1	0	0	0	0	0	0	0	0	0	0	0	0	0	0	0	0	0
1	1	07/03/21	08/03/21	1	0	0	8	7	51	1	6	5	2	2	1	0	1	0	0	1	1	1	0	0	0	0	1	1	0	0	0	0	0	0	0	0	1	0	0	0	1	0	0	0	0	0	0	0	0	0	0	0	0	0	0	0
1	1	07/03/21	08/03/21	1	1	1	9	8	55	1	6	6	7	8	8	0	0	0	0	0	1	0	0	0	0	0	1	0	0	0	1	0	0	0	0	0	1	0	0	0	0	0	0	0	0	0	0	0	0	0	0	0	0	0	1	0
1	1	07/03/21	08/03/21	1	0	0	16	15	72	0	6	5	4	2	1	0	0	0	0	0	1	0	0	0	0	0	1	0	0	1	0	0	0	0	0	1	0	0	0	0	0	0	0	0	0	0	0	0	0	0	0	0	0	0	0	0
1	1	08/03/21	08/03/21	0	0	0	5	5	53	1	6	3	1	1	1	0	0	0	0	0	1	0	0	0	0	0	1	1	0	0	0	0	0	0	0	0	1	0	1	0	1	0	0	0	1	0	0	0	0	0	0	0	0	0	0	0
1	1	08/03/21	08/03/21	0	1	1	7	7	84	1	6	7	7	8	8	1	1	1	0	4	1	0	0	0	0	0	1	1	0	0	1	0	0	0	0	1	1	0	1	0	1	0	0	0	0	0	0	0	0	0	0	0	0	0	1	0
1	1	02/03/21	08/03/21	6	0	0	9	3	74	1	5	2	1	1	1	0	0	0	0	0	1	0	1	0	0	0	1	0	0	0	1	0	0	0	0	0	1	0	0	0	0	0	0	0	0	0	1	0	0	0	0	0	0	0	0	0
1	1	07/03/21	08/03/21	1	0	0	6	5	42	1	5	5	2	2	1	0	1	0	0	1	1	0	1	0	0	0	1	0	1	0	0	0	1	0	0	1	0	0	1	1	1	1	1	0	0	0	0	0	0	0	0	0	0	0	0	0
1	1	05/03/21	08/03/21	3	0	0	3	0	80	1	5	2	2	1	1	0	1	0	0	1	1	0	1	0	0	0	1	0	1	0	0	0	1	0	0	1	0	0	1	1	1	1	1	0	0	0	0	0	0	0	0	0	0	0	0	0
1	1	05/03/21	08/03/21	3	1	1	8	5	25	1	5	6	8	8	8	0	0	0	0	1	1	0	1	0	0	0	1	0	1	0	0	0	1	0	0	1	0	0	1	1	1	1	1	0	0	0	0	0	0	0	0	0	0	0	1	0
1	1	07/03/21	08/03/21	1	0	0	4	3	78	1	5	2	2	2	1	0	1	0	0	1	1	0	1	0	0	0	1	0	1	0	0	0	1	0	0	1	0	0	1	1	1	1	1	0	0	0	0	0	0	0	0	0	0	0	0	0
1	1	06/03/21	08/03/21	2	0	0	4	2	43	1	6	2	1	1	1	0	0	0	0	0	1	0	1	0	0	0	1	0	1	0	0	0	1	0	0	1	0	0	1	1	1	1	1	0	0	0	0	0	0	1	0	1	0	0	1	0
1	0	5-Mar-2021	8-Mar-2021	3	1	1	7	4	50	1	5	5	8	8	8	0	0	0	0	0	1	0	1	1	1	1	1	1	0	1	0	0	0	0	0	1	0	0	0	0	0	0	0	0	0	0	0	0	0	0	0	0	0	0	0	0
1	0	5-Mar-2021	8-Mar-2021	3	0	0	7	4	49	0	5	3	2	1	1	0	0	0	0	0	1	0	1	1	1	1	1	1	0	1	0	0	0	0	0	1	0	0	0	0	0	0	0	0	0	0	0	0	0	0	0	0	0	0	0	0
1	0	7-Mar-2021	8-Mar-2021	1	0	0	3	2	50	1	6	2	1	1	1	0	0	0	0	0	1	0	1	1	1	1	1	1	0	1	0	0	0	0	0	0	1	0	0	0	0	0	0	0	0	0	1	0	0	0	0	0	0	0	0	0
1	0	7-Mar-2021	8-Mar-2021	1	0	0	5	4	50	0	5	3	2	1	1	0	0	0	0	0	1	0	1	1	1	1	1	1	0	1	0	0	0	0	0	1	0	0	0	0	0	0	0	0	0	0	0	0	0	0	0	0	0	0	0	0
0	1	07/03/21	09/03/21	2	0	0	14	12	51	1	5	2	2	2	1	0	0	0	0	0	1	0	1	0	0	0	1	0	1	0	0	0	1	0	0	1	0	0	1	1	1	1	1	0	1	0	0	0	0	0	0	0	0	0	0	0
1	1	08/03/21	09/03/21	1	0	0	6	5	57	1	5	4	1	1	1	1	1	0	0	2	1	0	0	0	0	0	1	0	0	0	1	0	0	0	1	0	1	0	1	0	1	0	0	0	0	0	1	0	0	0	0	0	0	0	0	0
1	1	03/03/21	09/03/21	6	1	1	13	7	67	1	6	7	8	8	8	1	1	0	0	3	1	0	0	0	0	0	1	0	0	0	0	0	0	1	0	0	1	0	0	0	0	0	0	1	0	0	0	0	0	0	0	0	0	0	1	0
1	1	08/03/21	09/03/21	1	0	0	3	2	43	1	6	2	2	2	1	0	0	0	0	0	1	0	1	0	0	0	1	0	1	0	0	0	1	0	0	1	0	0	1	1	1	1	1	0	0	0	1	0	0	0	0	0	0	0	0	0
1	0	9-Mar-2021	9-Mar-2021	4	0	0	12	12	57	1	6	5	4	2	1	0	0	0	0	0	1	0	1	1	1	1	1	1	1	0	0	0	0	0	0	1	0	0	0	0	0	0	0	0	0	0	0	0	0	0	0	0	0	0	0	0
1	0	9-Mar-2021	9-Mar-2021	0	0	0	4	4	53	1	6	2	2	2	2	0	1	0	0	1	1	0	1	1	1	1	1	1	0	1	0	0	0	0	0	0	1	0	0	0	0	0	0	0	0	0	0	0	0	0	0	0	0	0	0	0
0	1	08/03/21	10/03/21	2	0	0	11	9	61	1	6	5	4	7	7	0	0	0	0	0	1	0	0	0	0	0	1	1	0	0	1	0	0	0	0	0	1	0	1	0	1	0	0	0	0	0	0	0	0	1	0	0	0	0	1	0
1	1	10/03/21	10/03/21	0	0	0	5	5	50	1	4	3	1	1	1	0	0	0	0	0	1	0	0	0	0	0	1	0	0	0	1	0	0	0	0	0	1	0	0	0	0	0	0	0	0	0	0	0	0	0	0	0	0	0	0	0
1	1	09/03/21	10/03/21	1	0	0	8	7	55	1	6	4	2	1	1	0	0	0	0	0	1	0	0	0	0	0	1	0	0	0	1	0	0	0	0	0	1	0	1	0	1	0	0	0	0	0	0	0	0	0	0	0	0	0	0	0
1	1	09/03/21	10/03/21	1	0	0	6	5	44	1	5	3	1	1	1	0	0	0	0	0	1	0	0	0	0	0	1	0	0	0	1	0	0	0	0	0	1	0	0	0	0	0	0	0	0	0	0	0	0	0	0	0	0	0	0	0
1	1	08/03/21	10/03/21	2	0	0	3	1	62	1	6	1	1	1	1	0	1	0	0	1	1	0	1	0	0	0	1	0	1	0	0	0	1	0	0	1	0	0	1	1	1	1	1	0	0	0	0	0	0	0	0	0	0	0	1	0
1	1	09/03/21	10/03/21	1	0	0	5	4	51	1	6	4	2	1	1	0	0	0	0	0	1	0	1	0	0	0	1	0	1	0	0	0	1	0	0	1	0	0	1	1	1	1	1	0	0	0	0	0	0	0	0	0	0	0	0	0
0	1	11/03/21	11/03/21	0	1	1	16	16	63	1	5	7	7	8	8	0	1	0	0	1	1	0	0	0	0	0	1	0	0	0	1	0	0	1	1	0	1	0	1	0	0	0	0	0	1	0	0	0	0	0	0	0	0	0	1	0
0	1	10/03/21	11/03/21	1	0	0	13	12	63	1	5	5	5	2	1	1	0	0	0	1	1	0	0	0	0	0	1	0	0	0	1	0	1	0	0	0	1	0	1	0	1	0	0	0	0	0	0	0	0	0	0	0	0	0	0	0
1	1	11/03/21	11/03/21	0	0	0	28	28	43	1	6	7	7	4	1	0	0	0	0	0	1	0	0	0	0	0	1	1	0	0	0	0	0	0	0	0	1	0	1	0	0	0	0	0	0	0	0	0	0	0	0	0	0	0	1	0
1	1	11/03/21	11/03/21	0	0	0	5	5	45	0	6	3	1	1	1	0	0	0	0	0	1	0	0	0	0	0	1	1	0	0	0	0	0	0	0	0	1	0	0	0	0	0	0	0	0	0	0	0	0	0	0	0	0	0	0	0
1	1	11/03/21	11/03/21	0	0	1	15	15	72	0	6	6	7	7	8	1	0	0	0	2	1	0	1	0	0	0	1	0	1	0	0	0	1	0	0	1	0	0	1	1	1	1	1	0	0	0	1	0	0	0	0	0	0	0	1	0
1	1	11/03/21	11/03/21	0	1	1	3	3	55	1	6	8	8	8	8	0	1	0	0	2	1	0	1	0	0	0	1	0	1	0	0	0	1	0	0	1	0	0	1	1	1	1	1	0	0	0	0	0	0	0	0	0	0	0	1	0
1	1	08/03/21	11/03/21	3	0	0	9	6	45	0	6	5	2	2	1	0	0	1	0	2	1	0	0	0	1	1	1	1	0	1	0	0	0	0	0	1	0	1	0	1	1	0	0	0	0	0	0	0	0	0	0	0	0	0	0	0
1	0	3-Mar-2021	11-Mar-2021	8	0	0	40	32	54	1	5	6	6	5	5	1	0	0	0	1	1	0	1	1	1	1	1	1	0	1	0	0	0	0	0	1	0	0	0	0	0	0	0	0	0	0	0	0	0	0	0	0	0	0	0	0
1	0	11-Mar-2021	11-Mar-2021	0	1	1	1	1	44	1	5	8	8	8	8	0	0	0	0	0	1	0	1	1	1	1	1	1	0	1	0	0	0	0	0	1	0	0	0	0	0	0	0	0	0	0	0	0	0	0	0	0	0	0	0	0
1	0	9-Mar-2021	11-Mar-2021	2	1	1	13	11	55	1	6	5	4	8	8	0	0	0	0	0	1	0	1	1	1	1	1	1	0	1	0	0	0	0	0	1	0	0	0	0	0	0	0	0	0	0	0	0	0	0	0	0	0	0	0	0
0	1	04/03/21	12/03/21	8	0	0	12	4	60	1	5	4	2	2	1	0	0	0	1	2	1	0	0	0	0	0	1	0	0	0	0	0	1	0	0	0	1	0	1	0	0	0	0	0	0	0	0	0	0	0	0	0	0	0	0	0
0	1	11/03/21	12/03/21	1	1	1	8	7	77	1	6	7	8	8	8	1	1	1	0	3	0	1	0	0	0	0	1	0	0	0	1	0	0	0	1	0	1	0	0	0	0	0	0	1	0	0	0	0	0	0	0	0	0	0	1	0
0	1	09/03/21	12/03/21	3	0	0	18	15	38	1	6	5	4	4	2	0	0	0	0	0	1	0	1	0	0	0	1	0	1	0	0	0	1	0	0	1	0	0	1	1	1	1	1	0	0	0	0	0	0	0	0	0	0	0	0	0
1	1	09/03/21	12/03/21	3	0	0	13	10	52	1	6	5	4	2	1	1	0	0	0	1	0	1	0	0	0	0	1	0	0	0	1	0	0	0	0	0	1	0	0	0	0	0	0	0	0	0	0	0	0	0	0	0	0	0	0	0
1	1	12/03/21	12/03/21	0	0	0	9	9	54	1	5	4	3	1	1	0	0	0	0	0	1	0	0	0	0	0	1	0	0	0	1	0	0	0	0	0	1	0	1	0	0	0	0	0	0	0	1	0	0	0	0	0	0	0	0	0
1	1	12/03/21	13/03/21	1	0	0	7	6	62	1	6	4	2	1	1	0	1	0	0	1	1	0	0	0	0	0	1	1	0	0	0	0	0	0	0	0	1	0	0	0	1	0	0	0	0	0	0	0	0	0	0	0	0	0	0	0
1	1	12/03/21	13/03/21	1	0	0	7	6	54	1	4	4	2	1	1	0	0	0	0	0	1	0	0	0	0	0	1	1	0	0	0	0	0	0	0	0	1	0	1	0	0	0	0	0	0	0	0	0	0	0	0	0	0	0	0	0
1	1	13/03/21	13/03/21	0	0	0	8	8	48	1	5	4	2	1	1	0	1	0	0	1	1	0	0	0	0	0	1	1	0	0	0	0	0	0	0	0	1	0	0	0	1	0	0	0	0	0	0	0	0	0	0	0	0	0	0	0
1	1	12/03/21	13/03/21	1	0	0	4	3	45	0	5	2	1	1	1	0	0	0	0	1	1	0	1	0	1	1	1	0	0	0	0	0	0	0	0	0	1	0	0	0	0	0	0	0	0	0	1	0	0	0	0	0	0	0	0	0
1	0	13-Mar-2021	13-Mar-2021	0	0	0	3	3	71	1	6	2	1	1	1	0	1	0	0	1	1	0	1	1	1	1	1	1	0	1	0	0	0	0	0	1	0	0	0	0	0	0	0	0	0	0	1	0	0	0	0	0	0	0	0	0
1	0	13-Mar-2021	13-Mar-2021	0	0	0	3	3	64	0	5	2	2	1	1	0	1	0	0	1	1	0	1	1	1	1	1	1	0	1	0	0	0	0	0	1	0	0	0	0	0	0	0	0	0	0	0	0	0	0	0	0	0	0	0	0
1	1	13/03/21	14/03/21	1	0	0	27	26	45	1	6	7	7	5	1	1	1	1	0	3	1	1	0	0	0	0	1	0	0	0	1	0	0	0	0	0	1	0	1	0	1	0	0	0	0	0	0	0	0	0	0	0	0	0	1	0
1	1	13/03/21	14/03/21	1	0	0	6	5	73	1	6	6	6	3	2	1	1	0	0	2	0	0	1	0	0	0	1	0	0	0	1	0	0	0	0	0	1	0	1	0	0	0	0	0	0	0	0	0	0	0	1	1	0	0	0	0
1	1	13/03/21	14/03/21	1	0	0	8	7	59	1	5	4	2	1	1	0	0	0	0	0	1	0	0	0	0	0	1	0	0	0	1	0	0	0	0	1	0	0	0	0	0	0	0	0	0	0	0	0	0	0	0	0	0	0	0	0
1	1	13/03/21	14/03/21	1	0	0	25	24	44	1	6	6	6	5	2	0	0	0	0	0	1	0	0	0	0	0	1	0	0	0	1	0	0	0	0	0	1	0	0	0	0	0	0	0	0	0	0	0	0	0	0	0	0	0	0	0
1	1	13/03/21	14/03/21	1	0	0	8	7	59	1	6	4	2	1	1	0	0	0	0	0	1	0	0	0	0	0	1	1	0	0	0	0	0	0	0	0	1	0	0	0	0	0	0	0	0	0	1	0	0	0	0	0	0	0	0	0
1	1	12/03/21	14/03/21	2	0	0	9	7	53	0	5	4	2	2	1	0	1	0	0	1	0	1	0	0	0	0	1	1	0	0	0	0	0	0	0	0	1	0	1	1	0	0	0	0	0	0	1	0	0	0	0	0	0	0	0	0
1	1	13/03/21	14/03/21	1	0	0	5	4	68	0	6	2	1	1	1	0	0	0	0	0	1	0	0	0	0	0	1	0	0	0	0	0	0	0	0	0	1	0	0	0	0	0	0	0	0	0	0	0	0	0	0	0	0	0	0	0
1	1	11/03/21	14/03/21	3	0	0	16	13	45	0	5	5	5	1	1	0	0	1	0	1	1	0	1	0	1	1	1	1	0	1	0	0	0	0	1	1	0	0	1	1	1	0	0	0	0	0	0	0	0	0	0	0	0	0	0	0
1	0	10-Mar-2021	14-Mar-2021	4	0	0	11	7	29	0	5	4	2	1	1	0	0	0	0	0	1	0	1	1	1	1	1	1	0	1	0	0	0	0	0	1	0	0	0	0	0	0	0	0	0	0	0	0	0	0	0	0	0	0	0	0
1	0	13-Mar-2021	14-Mar-2021	1	0	0	8	7	36	1	5	3	2	2	1	0	0	0	0	0	1	0	1	1	1	1	1	1	0	1	0	0	0	0	0	1	0	0	0	0	0	0	0	0	0	0	1	0	0	0	0	0	0	0	0	0
1	0	8-Mar-2021	14-Mar-2021	6	0	0	26	20	46	0	5	6	5	4	1	0	0	0	0	0	1	0	1	1	1	1	1	1	0	1	0	0	0	0	0	1	0	0	0	0	0	0	0	0	0	0	0	0	0	0	0	0	0	0	0	0
0	1	11/03/21	15/03/21	4	1	1	13	9	77	1	6	6	7	8	8	0	1	0	0	2	1	0	1	0	0	0	1	0	1	0	0	0	1	0	0	1	0	0	1	1	1	1	1	0	0	0	0	0	0	0	0	0	0	0	1	0
0	1	13/03/21	15/03/21	2	0	0	12	10	59	1	6	5	1	2	2	1	0	0	0	1	1	0	1	0	0	0	1	0	1	0	0	0	1	0	0	1	0	0	1	1	1	1	1	0	0	0	0	0	0	0	0	0	0	0	0	0
0	1	10/03/21	15/03/21	5	1	1	13	8	68	1	6	7	8	8	8	0	0	0	0	0	1	0	1	0	0	0	1	0	1	0	0	0	1	0	0	1	0	0	1	1	1	1	1	0	0	0	0	0	0	0	0	0	0	0	1	0
1	1	14/03/21	15/03/21	1	0	0	9	8	55	1	6	5	2	1	1	0	0	0	0	1	1	0	0	0	0	0	1	1	0	0	0	0	0	0	0	0	1	0	1	0	0	0	0	0	0	0	0	0	0	0	0	0	0	0	0	0
1	1	14/03/21	15/03/21	1	0	0	8	7	61	0	6	4	2	1	1	0	0	0	0	0	1	0	0	0	0	0	1	1	0	0	0	0	0	0	0	0	1	0	1	0	0	0	0	0	0	0	0	0	0	0	0	0	0	0	0	0
1	1	10/03/21	15/03/21	5	0	0	7	2	41	0	6	2	1	1	1	0	0	0	0	0	1	0	1	0	0	0	1	0	1	0	0	0	1	0	0	1	0	0	1	1	1	1	1	0	0	0	0	0	0	0	0	0	0	0	0	0
1	1	14/03/21	15/03/21	1	0	0	3	2	30	1	5	2	1	1	1	0	0	0	0	0	1	0	1	0	0	0	1	0	1	0	0	0	1	0	0	1	0	0	1	1	1	1	1	0	0	0	0	0	0	0	0	0	0	0	0	0
1	1	12/03/21	15/03/21	3	0	0	6	3	48	1	6	2	1	1	1	0	1	0	0	1	1	0	1	0	0	0	1	0	1	0	0	0	1	0	0	1	0	0	1	1	1	1	1	0	0	0	0	0	0	0	0	0	0	0	0	0
1	1	15/03/21	15/03/21	0	0	0	5	5	32	1	6	5	1	1	1	0	0	0	0	0	1	0	1	0	0	0	1	0	1	0	0	0	1	0	0	1	0	0	1	1	1	1	1	0	0	0	1	0	0	0	0	0	0	0	0	0
1	1	11/03/21	15/03/21	4	0	0	7	3	80	1	5	2	1	1	1	0	0	0	0	1	1	0	1	0	0	0	1	0	1	0	0	0	1	0	0	1	0	0	1	1	1	1	1	0	0	0	0	0	0	0	0	1	0	0	0	1
1	1	14/03/21	15/03/21	1	0	0	4	3	52	1	5	2	1	1	1	0	0	0	0	0	1	0	1	0	0	0	1	0	1	0	0	0	1	0	0	1	0	0	1	1	1	1	1	0	0	0	0	0	0	0	0	0	0	0	0	0
0	1	16/03/21	16/03/21	0	0	0	16	16	65	1	6	6	6	2	2	0	0	0	0	0	1	0	1	0	0	0	1	0	1	0	0	0	1	0	0	1	0	0	1	1	1	1	1	0	0	0	0	0	0	0	0	0	0	0	0	0
0	1	15/03/21	16/03/21	1	0	0	82	81	55	0	5	6	6	6	6	0	1	1	0	3	1	0	1	1	1	1	1	1	0	0	0	1	0	1	1	1	1	0	1	1	1	0	0	0	0	0	0	0	0	0	0	0	0	0	1	0
1	1	15/03/21	16/03/21	1	0	0	7	6	72	1	5	3	1	1	1	0	1	1	0	2	1	0	0	0	0	0	1	0	0	0	0	1	0	0	0	0	1	0	1	0	0	0	0	0	0	0	1	0	0	0	0	0	0	0	0	0
1	1	15/03/21	16/03/21	1	0	0	8	7	38	1	5	4	2	1	1	0	0	0	0	0	1	0	0	0	0	0	1	0	0	0	0	0	0	0	0	0	1	0	1	0	0	0	0	0	1	0	0	0	0	0	0	0	0	0	0	0
1	1	15/03/21	16/03/21	1	0	0	3	2	34	0	6	5	1	1	1	0	0	0	1	1	1	0	1	0	0	0	1	0	1	0	0	0	1	0	0	1	0	0	1	1	1	1	1	0	0	0	0	0	0	0	0	0	0	0	0	0
1	1	15/03/21	16/03/21	1	0	0	3	2	54	0	5	2	1	1	1	0	0	0	0	0	1	0	1	0	0	0	1	0	1	0	0	0	1	0	0	1	0	0	1	1	1	1	1	0	0	0	0	0	0	0	0	0	0	0	0	0
1	1	16/03/21	16/03/21	0	0	0	4	4	58	1	5	2	1	1	1	0	0	0	0	0	1	0	1	0	0	0	1	0	1	0	0	0	1	0	0	1	0	0	1	1	1	1	1	0	0	0	1	0	0	0	0	0	0	0	0	0
1	1	11/03/21	16/03/21	5	0	0	9	4	46	1	6	2	1	1	1	0	0	0	0	0	1	0	1	0	0	0	1	0	1	0	0	0	1	0	0	1	0	0	1	1	1	1	1	0	0	0	0	0	0	0	0	0	0	0	0	0
1	1	14/03/21	16/03/21	2	0	0	9	7	51	0	5	4	2	1	1	0	1	1	0	2	1	0	0	0	1	1	1	0	0	0	0	1	0	0	0	1	0	0	0	1	1	0	0	0	0	0	0	0	0	0	0	0	0	0	0	0
1	1	15/03/21	16/03/21	1	0	0	12	11	75	0	4	4	4	4	2	0	1	1	0	3	1	0	0	0	1	1	1	0	0	0	0	1	0	0	0	1	0	1	0	1	0	0	0	0	0	0	0	0	0	0	0	0	0	0	0	0
1	1	15/03/21	16/03/21	1	0	0	23	22	67	0	4	6	6	6	4	1	1	1	0	4	1	0	0	0	1	1	1	1	0	1	0	0	0	1	1	1	0	0	0	0	0	0	0	0	0	0	0	0	0	0	0	0	0	0	1	0
1	1	15/03/21	17/03/21	2	0	0	8	6	43	1	4	4	1	1	1	0	0	1	0	0	1	0	0	0	1	1	1	0	0	0	0	0	0	0	0	1	0	0	0	0	1	0	0	0	0	0	0	0	0	0	0	0	0	0	0	0
1	0	15-Mar-2021	17-Mar-2021	2	0	0	22	20	47	0	6	6	5	5	2	0	0	0	0	0	1	0	1	1	1	1	1	1	0	1	0	0	0	0	0	1	1	0	0	0	0	0	0	0	0	0	0	0	0	0	0	0	0	0	0	0
